# Rationalisation of Patterns of Competing Reactivity by X-ray Structure Determination: Reaction of Isomeric (Benzyloxythienyl)oxazolines with a Base

**DOI:** 10.3390/molecules26247690

**Published:** 2021-12-20

**Authors:** R. Alan Aitken, Andrew D. Harper, Alexandra M. Z. Slawin

**Affiliations:** EaStCHEM School of Chemistry, University of St Andrews, North Haugh, St Andrews KY16 9ST, Fife, UK; andydharper@hotmail.com (A.D.H.); amzs@st-and.ac.uk (A.M.Z.S.)

**Keywords:** oxazoline, Wittig rearrangement, thiophene, thieno[2,3-*c*]pyrrolone, X-ray structure

## Abstract

Three isomeric (benzyloxythienyl)oxazolines **9**, **11** and **13** have been prepared and are found, upon treatment with a strong base, to undergo either Wittig rearrangement or intramolecular attack of the benzylic anion on the oxazoline function to give products derived from cleavage of the initially formed 3-aminothienofuran products. This pattern of reactivity is directly linked to the distance between the two reactive groups as determined by X-ray diffraction, with the greatest distance in **11** leading to exclusive Wittig rearrangement, the shortest distance in **13** giving exclusively cyclisation-derived products, and the intermediate distance in **9** leading to both processes being observed. The corresponding *N*-butyl amides were also obtained in two cases and one of these undergoes efficient Wittig rearrangement leading to a thieno[2,3-*c*]pyrrolone product.

## 1. Introduction

**Scheme 1:**
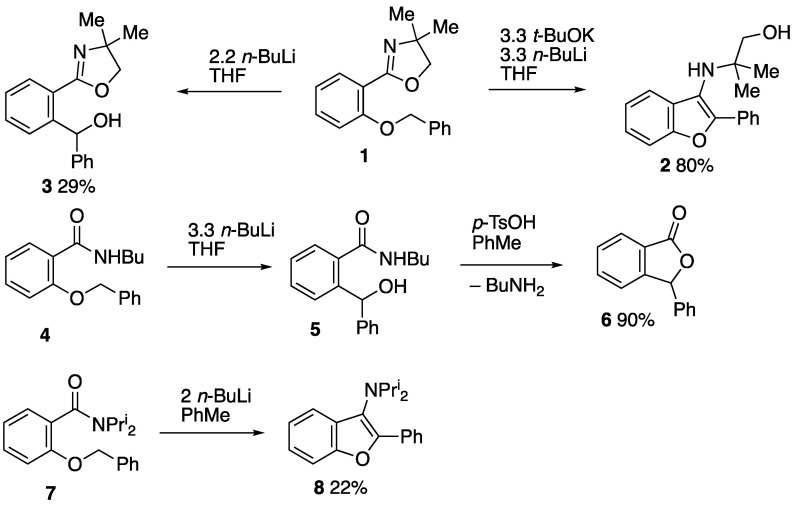
Competition between Wittig rearrangement and cyclisation in benzene-based systems.

Some time ago we described the reaction of 2-(2-benzyloxyphenyl)oxazoline **1** with a strong base to give either the 3-aminobenzofuran product **2** resulting from intramolecular nucleophilic ring-opening of the oxazoline by the benzyl anion, or the oxazoline **3** in which the benzyloxy group has undergone a Wittig rearrangement (Scheme 1) [1]. Heterocycle formation by cyclisation of an aryloxy carbanion onto an *ortho* functional group is rather uncommon but formation of 3-aminobenzofurans by the so-called Gewald reaction of benzonitriles provides one example [2]. Similarly, although the Wittig rearrangement has been known for almost a century [3], it is not commonly used in synthesis and a recent review shows rather limited developments over the last 20 years [4]. While the aminobenzofuran formation could be optimised by using 3.3 equiv. of Schlosser’s base (*n*-BuLi/*t*-BuOK) and applied to a number of substituted examples [1], the Wittig rearrangement process was not so favourable and, under optimal conditions of 2.2 equiv. butyllithium (*n*-BuLi) in THF, an isolated yield of just 29% was obtained. As will shortly be reported elsewhere, the *N*-butyl amide group is a more effective promoter of the Wittig rearrangement and treating compound **4** with 3.3 equiv. *n*-BuLi in THF gives almost entirely the rearranged product **5**, conveniently isolated as the phthalide **6** after acid-mediated cyclisation in 90% yield. However in the latter study, simply changing to the *N*,*N*-diisopropyl amide **7** and treating with 2.2 equiv. *n*-BuLi in toluene again resulted in cyclisation to give the 3-aminobenzofuran **8**. It is clear from these studies that there is a delicate balance between Wittig rearrangement of the benzyloxy group without affecting the adjacent activating group, and interaction of the two groups with the formation of a furan ring.

**Scheme 2:**
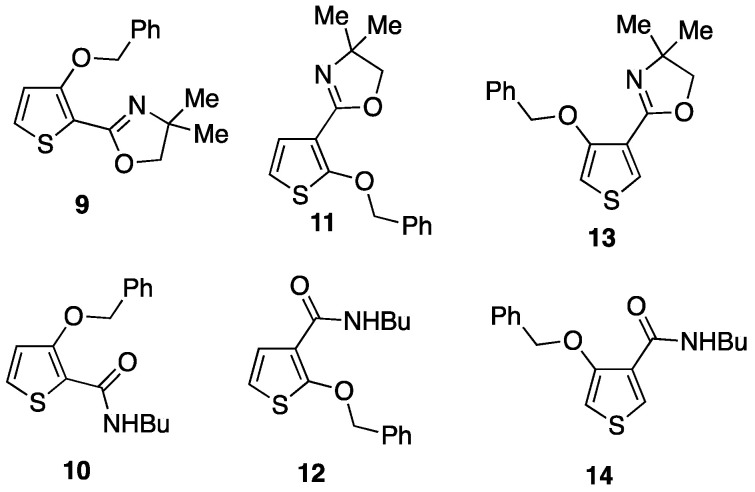
The six isomeric thiophene compounds targeted for reactivity studies.

In contrast to the symmetrical benzene ring, the different adjacent positions on a heterocycle such as thiophene are not equivalent and so a more interesting pattern of reactivity can be expected, which would also lead to some unusual and novel heterocyclic products. In this paper, we report the synthesis, characterisation and reactivity upon treatment with a strong base, of the three isomeric (benzyloxythienyl)oxazolines **9**, **11** and **13** (Scheme 2) as well as the corresponding (benzyloxythienyl)-*N*-butylcarboxamides **10**, **12** and **14**. As well as examining the pattern of reactivity we were interested to discover whether there was any correlation between this and the distance between the two adjacent groups as determined by X-ray diffraction.

## 2. Results

### 2.1. Synthesis of 3-Benzyloxy-2-thienyl Systems ***9*** and ***10***

**Scheme 3:**
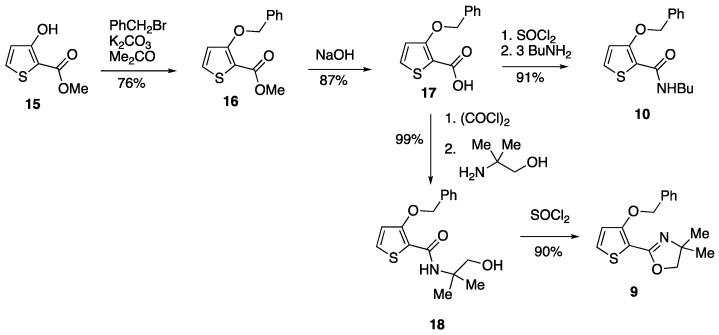
Synthesis of 3-benzyloxy-2-thienyl compounds **9** and **10**.

*O*-Benzylation of the commercially available methyl ester **15** followed by ester hydrolysis of **16** gave the carboxylic acid **17** (Scheme 3). This was readily converted into the corresponding acid chloride which was reacted immediately with 2-amino-2-methylpropan-1-ol to give the hydroxy amide **18**, which was cyclised using thionyl chloride to give the target oxazoline **9** in good overall yield. Alternatively, treating acid **17** with thionyl chloride followed by an excess of butylamine gave the target amide **10** also in high yield.

### 2.2. Reaction 3-Benzyloxy-2-thienyl Systems ***9*** and ***10*** with Base

**Scheme 4:**
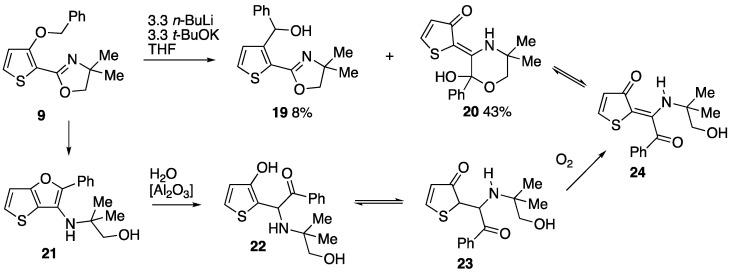
Reaction of oxazoline **9** with *n*-BuLi/*t*-BuOK.

When compound **9** was subjected to the same conditions used to convert **1** into **2**, a mixture of two products was formed which were separated by preparative thin-layer chromatography (TLC) on alumina and identified as the expected Wittig rearrangement product **19**, formed in 8% yield, and a second more major product (43%) which was initially thought to be the expected cyclisation product **21** (Scheme 4). However certain features of the spectra were not consistent with this, notably the non-equivalence of the two methyl groups and CH_2_O hydrogens which suggested the presence of a stereogenic centre. After further evidence from ^13^C and 2D NMR studies suggested the presence of a 2-alkylidenethiophen-3(2*H*)-one structure, and the HRMS result showed the presence of an extra oxygen atom as compared to **21**, the actual structure was finally confirmed as the unusual morpholine-containing thiophenone **20** by X-ray diffraction.

**Figure 1:**
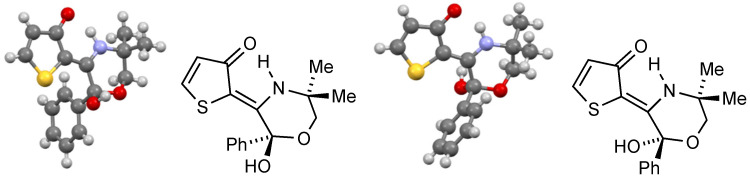
Conformations of the two enantiomeric molecules of **20** in the crystal.

The molecular structure of **20** features two independent molecules in the unit cell in addition to one molecule each of CH_2_Cl_2_ and acetone. The two molecules are actually enantiomers and they both take up half chair conformations with the ring oxygen out of the plane, the NH in the plane and one methyl axial and one equatorial (Figure 1). Where they differ is that one has phenyl axial and OH equatorial while for the other it is the opposite way round.

**Figure 2:**
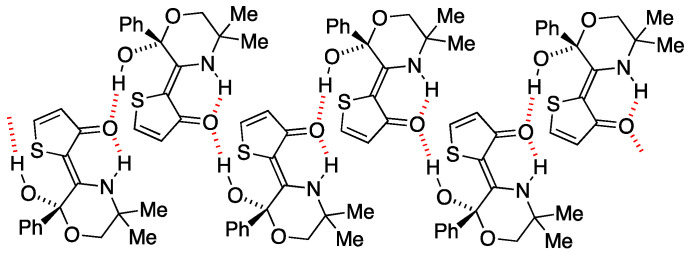
Schematic representation of the hydrogen bonding pattern for **20**.

In the crystal, there is hydrogen bonding both intramolecularly between the NH and C=O and intermolecularly between C=O and OH; in terms of the Etter–Bernstein graph-set descriptors [5] C^1^_1_(7) [S(6)]. The intermolecular interaction involves the two enantiomeric molecules alternating and the pattern is shown schematically in Figure 2 with parameters in Table 1.

Once the structure of **20** was clear, its formation could be rationalised as shown in Scheme 4 by initial cyclisation of **9** to give the thieno[3,2-*b*]furan product **21** and hydrolysis of this on the alumina with opening of the furan ring to give **22**, which in its thiophen-3-one tautomeric form can be oxidised by air to form the favourable fully conjugated ene-dione structure **24**, which then cyclises to form the cyclic hemiketal **20**. It should be noted that compound **20** was found to be quite unstable and although it was isolated in small amount with sufficient purity for identification, further attempts at purification resulted in decomposition. As described in a recent review [6], thiophene-based *o*-quinomethane analogues have a rich and varied chemistry, however, the formation of such a structure by hydrolysis then oxidation of a thieno[3,2-*b*]furan is unprecedented.

**Scheme 5:**
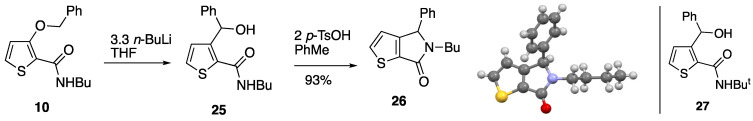
Reaction of **10** with *n*-BuLi to give **25** and **26** and structure of an analogous product **27**.

We now turned to the *N*-butyl amide **10** and found a much simpler pattern of reactivity. Treatment of **10** with *n*-BuLi under the standard conditions developed in the benzene series, resulted in exclusive Wittig rearrangement to give secondary alcohol **25** which could be characterised spectroscopically but was converted for isolation into the stable thieno[2,3-*c*]pyrrolone product **26** by treatment with *p*-toluenesulfonic acid in boiling toluene (Scheme 5) [7]. This method was also used to obtain stable cyclic products in the benzene-based systems, however, note that while **5** and analogues cyclise to lactones **6** with loss of butylamine, here we have a loss of water to form the lactam in excellent yield. The synthesis and chemistry of thieno[*c*]pyrrolones and their dihydro analogues has been recently reviewed [8].

Since such fused-ring heterocycles are rather uncommon we took the chance to determine the X-ray structure of compound **26** (Scheme 5). Only one previous X-ray structure of a compound with this ring system appears to have been published, that of the compound with butyl replaced by quinolin-8-yl [9], but the molecular dimensions are very similar. Interestingly the *tert*-butyl amide **27** isomeric with **25** has been prepared by *ortho*-directed metalation of *N-tert*-butylthiophene-2-carboxamide with *n*-BuLi followed by reaction with benzaldehyde [10].

### 2.3. Synthesis of 2-Benzyloxy-3-thienyl Systems ***11*** and ***12***

**Scheme 6:**
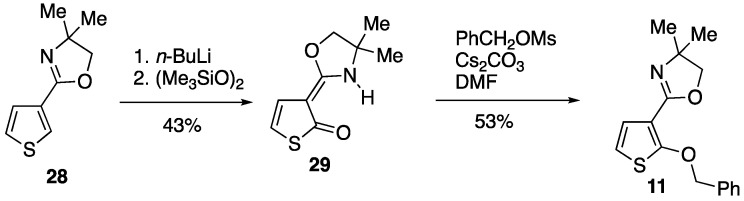
Synthesis of oxazoline **11**.

Entry to this system was gained by starting with the 3-thienyloxazoline **28** and introducing oxygen functionality at the 2-position by lithiation and treatment with bis(trimethylsilyl) peroxide (Scheme 6). As we have described in detail elsewhere [11], the resulting product had the 3-(oxazolidin-2-ylidene)thiophen-2-one structure **29** which exhibited an interesting and varied pattern of reactivity. However, for the present purpose, it could be cleanly *O*-benzylated in moderate yield to give the desired compound **11**.

As described below, attempted application of a similar method to the formation of the amide **12** failed since lithiation of the corresponding *N*-butyl amide **32** followed by treatment with bis(trimethylsilyl) peroxide instead gave the silyl compound **33**.

### 2.4. Reaction of 2-Benzyloxy-3-thienyl Systems ***11*** and ***12*** with Base

**Scheme 7:**
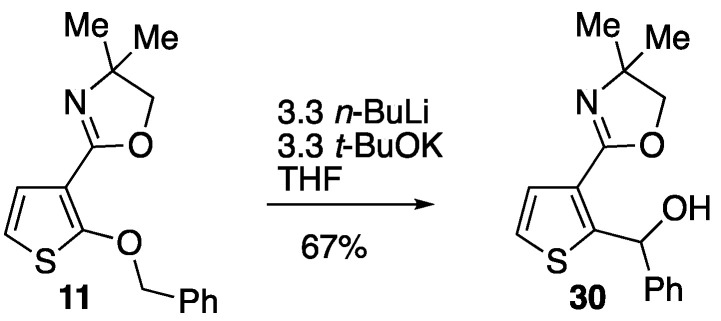
Wittig rearrangement of oxazoline **11**.

Treatment of oxazoline **11** with *n*-BuLi under the standard conditions developed for ring closure of **1** to give **2** gave exclusively the Wittig rearrangement product **30** in good yield (Scheme 7).

**Scheme 8:**
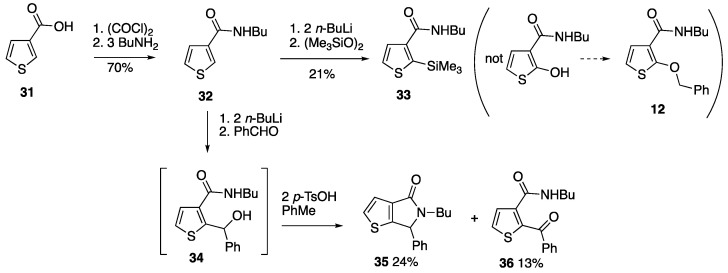
Formation and cyclisation of secondary alcohol **34**.

Although attempts to prepare the amide **12** by lithiation and bis(trimethylsilyl) peroxide treatment failed, instead giving the new silane **33** (Scheme 8), the expected Wittig rearrangement product from **12**, compound **34**, was prepared by lithiation and benzaldehyde treatment of **32**. It was not isolated, however, the reaction product was directly treated with *p*-toluenesulfonic acid giving the thieno[2,3-*c*]pyrrolone [8] product **35** isomeric with **26** together with a low yield of the oxidation product **36**. This last product showed extra signals in the ^13^C NMR spectrum due to amide rotamers (Supplementary Materials).

### 2.5. Synthesis of 4-Benzyloxy-3-thienyl Systems ***13*** and ***14***

**Scheme 9:**
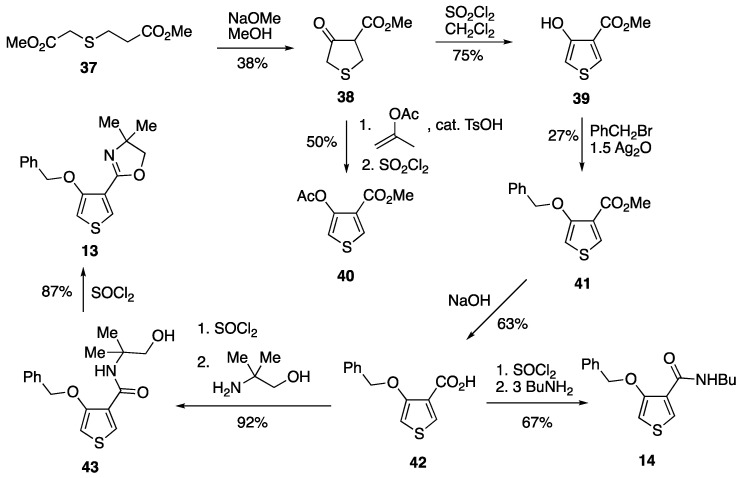
Synthesis of 4-benyloxy-3-thienyl compounds **13** and **14**.

Synthesis of the required compounds in this series was more challenging since suitably substituted thiophene starting materials are not commercially available. Instead, we had to resort to a ring-synthesis of a thiophene with the desired functionality in place. This started from the sulfide-containing diester **37** prepared by conjugate addition of methyl thioglycolate to methyl acrylate [12], which underwent base-induced ring closure [13] to give compound **38** in low yield (Scheme 9). Aromatisation of this was achieved using sulfuryl chloride [14] to give the thiophene ester **39**. Conversion of this into the required benzyl ether **41** proved to be more difficult than expected. Simple alkylation using benzyl bromide and either potassium carbonate or sodium hydride resulted in polymerisation and reaction with phenyldiazomethane [15] also failed.

Following a literature report that reaction of the 4-acetoxy compound **40** with ethanol and sulfuric acid gave the 4-ethoxy compound [15], this compound was prepared by reaction of **38** with isopropenyl acetate followed by sulfuryl chloride, but the treatment of this with benzyl alcohol and sulfuric acid again resulted in polymerisation. Access to **41** was finally achieved, albeit in low yield, by resorting to treatment with benzyl bromide in the presence of silver oxide in a process reminiscent of the Purdie–Irvine method for methylation of sugars developed in St Andrews over 100 years ago [16]. With the key intermediate **41** in hand, the remaining synthetic steps proceeded without incident: hydrolysis gave the acid **42** which was converted into its acid chloride and then reacted either with 2-amino-2-methylpropan-1-ol to give amide **43** which was cyclised with thionyl chloride to oxazoline **13**, or with butylamine to directly afford the amide **14**.

### 2.6. Reaction of 4-Benzyloxy-3-thienyl Systems ***13*** and ***14*** with Base

**Scheme 10:**
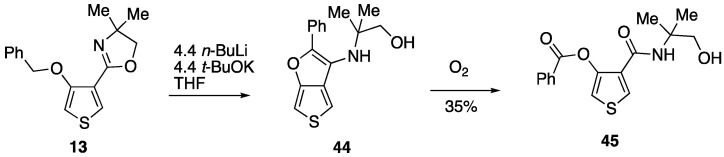
Base-induced cyclisation and oxidative ring opening of **13**.

Treatment of oxazoline **13** with 3.3 equiv. of Schlosser’s base gave largely unreacted starting material, however, increasing this to 4.4 equiv. did give a reaction and after chromatographic purification, the 4-benzoyloxy-3-thienyl amide **45** was isolated in moderate yield (Scheme 10). This is evidently formed by air oxidation of the expected cyclisation product, the 3-aminothieno[3,4-*b*]furan **44**. As shown in our previous work [1], such ring-fused 3-aminofuran products are susceptible to oxidative ring-cleavage.

**Scheme 11:**
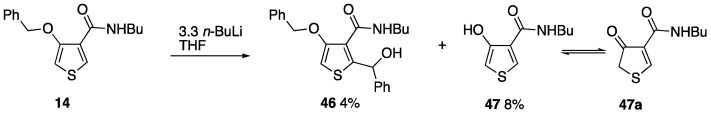
Reaction of amide **14** with strong base.

The reaction of the corresponding *N*-butyl amide **14** with *n*-BuLi under the conditions required for Wittig rearrangement gave largely unreacted starting material and the only new products isolated in low yield after chromatographic purification (Scheme 11) were the 2,3,4-trisubstituted thiophene **46** together with the debenzylated compound **47** which was found to exist in solution as a mixture with the thiophen-3(2*H*)-one tautomer **47a** (see Section 3). It seems likely that the products have resulted from the intermolecular reaction between two carbanions derived from **14** but in view of their very low yield this process was not investigated further. Products **45**, **46** and **47** which were isolated in low amounts following one or two stages of chromatography were found to decompose upon attempted further purification.

**Figure 3:**
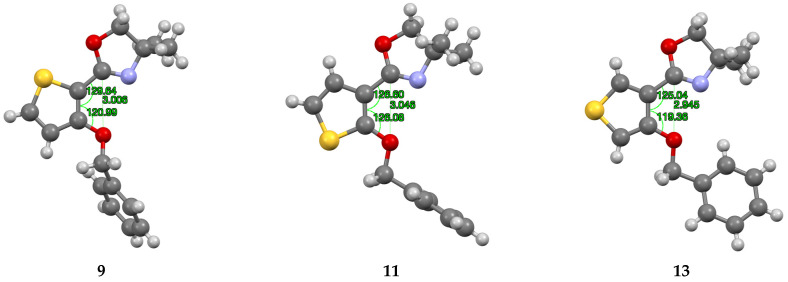
Molecular structures of **9**, **11** and **13** showing angles (°) and benzyloxy O to oxazoline C(2) distance (Å).

To summarise the reactivity of the isomeric systems, oxazoline **11** underwent exclusive Wittig rearrangement and oxazoline **13** gave products derived from cyclisation, while for **9** Wittig rearrangement was observed as a minor process with the major product derived from cyclisation. The *N*-butyl amides gave a less complete picture with **10** undergoing exclusive Wittig rearrangement in high yield, **12** not being available for investigation (although its expected Wittig rearrangement product was obtained by other means), and **14** remaining largely unreacted under the conditions. In the case of the three isomeric oxazolines, each compound was obtained as good quality crystals suitable for X-ray diffraction and so it was decided to determine their molecular structures to examine whether there might be a direct link between the distance between the benzyloxy and oxazoline groups and the observed reactivity. All three compounds gave structures with the monoclinic P2_1_/c space group and these are shown in a similar orientation in Figure 3.

For the intramolecular cyclisation to compete with Wittig rearrangement, the key distance is that between benzyloxy carbanionic carbon and C-2 of the oxazoline. Since the benzyloxy groups have rotated to place this carbon pointing away from the oxazoline in each case, the benzyloxy oxygen is taken as a reference point and it can be seen that the molecular geometry correlates well with the observed reactivity. Thus, for **11**, the benzyloxy group is too far away (3.046(1) Å) for cyclisation and we observe exclusively a Wittig rearrangement, for **13** the benzyloxy group is much closer (2.945(1) Å) and only products derived from cyclisation are observed, while in **9** we have an intermediate situation (3.006(3) Å) and mainly cyclisation-derived products are observed but with a little Wittig rearrangement.

## 3. Experimental

### 3.1. General Experimental Details

NMR spectra were recorded on solutions in CDCl_3_ unless otherwise stated using Bruker instruments and chemical shifts are given in ppm to high frequency from Me_4_Si with coupling constants *J* in Hz. IR spectra were recorded using the ATR technique on a Shimadzu IRAffinity 1S instrument. The ionisation method used for high-resolution mass spectra is noted in each case. Column chromatography was carried out using silica gel of 40–63 mm particle size and preparative TLC was carried out using 1.0 mm layers of Merck alumina 60G containing 0.5% Woelm fluorescent green indicator on glass plates. Melting points were recorded on a Gallenkamp 50W melting point apparatus or a Reichert hot-stage microscope.

### 3.2. Preparation and Reactions of 3-Benzyloxy-2-thienyl Systems

#### 3.2.1. Methyl 3-(Benzyloxy)thiophene-2-carboxylate **16**

A literature procedure [17] was modified as follows: benzyl bromide (11.9 cm^3^, 17.11 g, 0.100 mol) was added to a stirred mixture of methyl 3-hydroxythiophene-2-carboxylate **15** (15.85 g, 0.100 mol) and potassium carbonate (27.60 g, 0.200 mol) in acetone (50 cm^3^) and the reaction mixture was heated at reflux for 18 h. After cooling to rt, the inorganic salts were removed by filtration and the filtrate was concentrated *in vacuo*. The residue was dissolved in CH_2_Cl_2_ (150 cm^3^) and washed with water (100 cm^3^) before being dried and evaporated. The crude residue was recrystallised (aq. MeOH) to give **16** (18.94 g, 76%) as pale yellow crystals; mp 69–72 °C; (lit. [17] 66–67 °C); δ_H_ (500 MHz) 7.46–7.44 (2H, m, Ph), 7.39–7.35 (2H, m, Ph), 7.35 (1H, d, *J* 5.5, 5-H), 7.32–7.29 (1H, m, Ph), 6.82 (1H, d, *J* 5.5, 4-H), 5.24 (2H, s, CH_2_) and 3.85 (3H, s, CH_3_); δ_C_ (125 MHz) 162.1 (C), 160.8 (C), 136.4 (C), 130.4 (CH), 128.6 (2CH), 127.9 (CH), 126.8 (2CH), 117.5 (CH), 110.4 (C), 73.2 (CH_2_) and 51.6 (CH_3_). The ^1^H NMR spectral data were in accordance with those previously reported [17]. ^13^C NMR data are reported for the first time.

#### 3.2.2. 3-(Benzyloxy)thiophene-2-carboxylic Acid **17**

Following a literature procedure [17], a mixture of methyl 3-(benzyloxy)thiophene-2-carboxylate **16** (18.40 g, 74.1 mmol) and sodium hydroxide (5.99 g, 0.150 mol) in water (150 cm^3^) was heated at reflux for 2.5 h. After cooling to rt, the aqueous layer was washed with CH_2_Cl_2_ (2 × 50 cm^3^) before being acidified to pH 1 by the addition of 2 M HCl. The resultant suspension was extracted with CH_2_Cl_2_ (2 × 100 cm^3^) and the combined organic layers were dried and evaporated. The crude residue was recrystallised (aq. MeOH) to give **17** (15.07 g, 87%) as tan-coloured crystals; mp 122–125 °C; (lit. [17] 125–126 °C); δ_H_ (400 MHz, CD_3_SOCD_3_) 12.49 (1H, br s, CO_2_H), 7.74 (1H, d, *J* 5.6, 5-H), 7.48–7.45 (2H, m, Ph), 7.41–7.37 (2H, m, Ph), 7.35–7.30 (1H, m, Ph), 7.14 (1H, d, *J* 5.6, 4-H) and 5.25 (2H, s, CH_2_); δ_C_ (100 MHz, CD_3_SOCD_3_) 162.4 (C), 160.1 (C), 136.8 (C), 131.2 (CH), 128.4 (2CH), 127.9 (CH), 127.3 (2CH), 118.5 (Ar CH), 110.5 (C) and 72.4 (CH_2_). The ^1^H NMR spectral data were in accordance with those previously reported [17]. ^13^C NMR data are reported for the first time.

#### 3.2.3. 3-(Benzyloxy)-*N*-(1-hydroxy-2-methylpropan-2-yl)thiophene-2-carboxamide **18**

Oxalyl chloride (3.0 cm^3^, 4.50 g, 35.5 mmol) was added to a suspension of 3-(benzyloxy)thiophene-2-carboxylic acid **17** (4.00 g, 17.1 mmol) and in Et_2_O (25 cm^3^) and the mixture was stirred for 18 h. Evaporation gave 3-(benzyloxy)thiophene-2-carbonyl chloride as a brown oil which was used without further purification.

A solution of 3-(benzyloxy)thiophene-2-carbonyl chloride (assuming 17.1 mmol) in CH_2_Cl_2_ (40 cm^3^) was added dropwise to a solution of 2-amino-2-methylpropan-1-ol (3.10 g, 34.8 mmol) in CH_2_Cl_2_ (40 cm^3^) stirred at 0 °C. After the addition, the mixture was allowed to warm to rt and stirred for 18 h before being poured into water. The organic layer was separated and the aqueous layer was extracted with CH_2_Cl_2_ (2 × 20 cm^3^). The combined organic layers were washed successively with 2M HCl, 2M NaOH and water before being dried and evaporated to give **18** (5.14 g, 99%) as a pale yellow solid which was used without further purification; mp 112–115 °C; ν_max_/cm^–1^ 3358, 3065, 2968, 1626, 1531, 1425, 1240, 1057, 777, 704 and 600; δ_H_ (500 MHz) 7.43–7.40 (6H, m, NH and Ph), 7.41 (1H, d, *J* 5.5, 5-H), 6.93 (1H, d, *J* 5.5, 4-H), 5.18 (2H, s, OCH_2_Ph), 3.58 (2H, s, C***H***_2_OH) and 1.19 (6H, s, CH_3_); δ_C_ (125 MHz) 162.5 (C), 155.3 (C), 135.2 (C), 129.2 (CH), 129.0 (CH), 128.8 (2CH), 128.0 (2CH), 117.6 (C), 116.2 (CH), 74.1 (CH_2_), 70.9 (CH_2_), 56.3 (***C***Me_2_) and 24.8 (2CH_3_); HRMS (NSI^+^): found 306.1150. C_16_H_20_NO_3_S (M + H) requires 306.1158.

#### 3.2.4. 2-(3-(Benzyloxy)thiophen-2-yl)-4,4-dimethyl-4,5-dihydrooxazole **9**

Thionyl chloride (1.4 cm^3^, 2.28 g, 19.2 mmol) was added to a solution of 3-(benzyloxy)-*N*-(1-hydroxy-2-methylpropan-2-yl)thiophene-2-carboxamide **18** (4.71 g, 15.4 mmol) in CH_2_Cl_2_ (50 cm^3^) and the mixture was stirred at room temperature for 18 h. The mixture was washed with 2M NaOH and water before being dried and evaporated to give **9** (4.01 g, 90%) as a pale brown oil which solidified on standing as a tan-coloured solid; mp 65–68 °C; ν_max_/cm^–1^ 3080, 2965, 1632, 1545, 1260, 1231, 1200, 1069, 1026, 766 and 745; δ_H_ (500 MHz) 7.45–7.42 (2H, m, Ph), 7.37–7.33 (2H, m, Ph), 7.31–7.27 (1H, m, Ph), 7.24 (1H, d, *J* 5.5, 5-H), 6.78 (1H, d, *J* 5.5, 4-H), 5.24 (2H, s, OCH_2_Ph), 4.09 (2H, s, OCH_2_) and 1.38 (6H, s, CH_3_) δ_C_ (125 MHz) 157.6 (C), 157.3 (C), 136.8 (C), 128.4 (2CH), 127.8 (2CH), 126.9 (2CH), 118.1 (CH), 108.9 (C), 79.2 (CH_2_), 73.4 (CH_2_), 67.1 (***C***Me_2_) and 28.3 (2CH_3_); HRMS (ESI^+^): found 288.1047. C_16_H_18_NO_2_S (M + H) requires 288.1053.

#### 3.2.5. 3-(Benzyloxy)-*N*-butylthiophene-2-carboxamide **10**

Thionyl chloride (2.5 cm^3^, 4.08 g, 34.3 mmol) was added to a suspension of 3-(benzyloxy)thiophene-2-carboxylic acid **17** (4.00 g, 17.1 mmol) in toluene (30 cm^3^) and the mixture was heated under reflux for 3 h. After cooling to room temperature, the mixture was evaporated to give 3-(benzyloxy)thiophene-2-carbonyl chloride as a brown oil which was used without further purification.

A solution of 3-(benzyloxy)thiophene-2-carbonyl chloride (assuming 17.1 mmol) in toluene (30 cm^3^) was added dropwise to a solution of *n*-butylamine (5.1 cm^3^, 3.77 g, 51.6 mmol) in toluene (10 cm^3^) stirred at 0 °C. Once the addition was complete, the reaction mixture was allowed to warm to room temperature over 1 h before being poured into water. The organic layer was separated and washed with 2M NaOH and brine, dried and evaporated to give, after purification by column chromatography (SiO_2_, Et_2_O/hexane 7:3), at R_f_ 0.65, **10** (4.49 g, 91%) as an orange oil; ν_max_/cm^–1^ 3364, 2961, 1628, 1558, 1435, 1364, 1310, 1074, 976, 773 and 606; δ_H_ (500 MHz) 7.43–7.38 (5H, m, Ph), 7.36 (1H, d, *J* 5.5, 5-H), 7.19 (1H, br s, NH), 6.89 (1H, d, *J* 5.5, 4-H), 5.19 (2H, s, OCH_2_), 3.36 (2H, td, *J* 7.0, 5.5, NCH_2_), 1.47–1.41 (2H, m, NCH_2_C***H***_2_), 1.28–1.20 (2H, m, C***H***_2_CH_3_) and 0.85 (3H, t, *J* 7.5, CH_3_); δ_C_ (125 MHz) 161.7 (C), 154.9 (C), 135.6 (C), 128.84 (2CH), 128.76 (CH), 128.5 (CH), 127.7 (2CH), 118.1 (C), 116.2 (CH), 73.9 (OCH_2_), 38.9 (NCH_2_), 31.5 (CH_2_), 20.0 (CH_2_) and 13.7 (CH_3_); HRMS (ESI^+^): found 312.1017. C_16_H_19_NaNO_2_S (M + Na) requires 312.1029.

#### 3.2.6. (2-(4,4-Dimethyl-4,5-dihydrooxazol-2-yl)thiophen-3-yl)(phenyl)methanol **19** and (*E*)-2-(2-Hydroxy-5,5-dimethyl-2-phenylmorpholin-3-ylidene)thiophen-3(2*H*)-one **20**

Under a nitrogen atmosphere, *n*-butyllithium (2.5 M in hexanes, 0.66 cm^3^, 1.65 mmol) was added to a stirred mixture of 2-(3-(benzyloxy)thiophen-2-yl)-4,4-dimethyl-4,5-dihydrooxazole **9** (0.1440 g, 0.50 mmol) and potassium *tert*-butoxide (0.1850 g, 1.65 mmol) in dry THF (5 cm^3^). The mixture was stirred at rt for 2 h before being quenched by the addition of saturated aq. NH_4_Cl and extracted with Et_2_O (3 × 10 cm^3^). The combined extracts were dried and evaporated to give, after purification by preparative TLC (Al_2_O_3_, Et_2_O/hexane 7:3), at R_f_ 0.65, **19** (12 mg, 8%) as an orange oil; δ_H_ (400 MHz) 7.42–7.38 (2H, m, Ph), 7.34–7.30 (3H, m, ArH and Ph), 7.28–7.23 (1H, m, Ph), 6.71 (1H, d, *J* 5.2, ArH), 6.02 (1H, s, C***H***OH), 4.11 and 4.09 (2H, AB pattern, *J* 8.2, CH_2_), 1.40 (3H, s, CH_3_) and 1.27 (3H, s, CH_3_); δ_C_ (125 MHz) 158.6 (C), 150.4 (C), 142.8 (C), 130.1 (CH), 128.2 (CH), 128.0 (2CH), 127.1 (CH), 126.5 (2CH), 124.7 (C), 79.5 (CH_2_), 70.9 (CHOH), 68.1 (***C***Me_2_), 28.3 (CH_3_) and 28.1 (CH_3_). The ^1^H NMR spectral data were consistent with those previously reported [18]. ^13^C NMR data are reported for the first time.

This was followed by a second fraction, at R_f_ 0.15, to give **20** (64.5 mg, 42%) in slightly impure form as brown crystals; mp 103–105 °C; ν_max_/cm^–1^ 1582, 1537, 1449, 1317, 1260, 1221, 1067, 768, 698 and 669; δ_H_ (400 MHz, CD_3_COCD_3_) 7.65–7.62 (2H, m, Ph), 7.52 (1H, d, *J* 5.6, 5-H), 7.40–7.35 (3H, m, Ph), 6.32 (1H, d, *J* 5.6, 4-H), 4.12 and 3.69 (2H, AB pattern, *J* 11.6, CH_2_), 3.06 (2H, br s, OH and NH), 1.53 (3H, s, CH_3_) and 1.39 (3H, s, CH_3_); δ_C_ (125 MHz, CD_3_COCD_3_) 182.1 (C=O), 162.7 (C)), 141.5 (C), 138.5 (CH), 129.5 (CH), 128.6 (2CH), 127.6 (2CH), 122.1 (CH), 103.5 (C), 95.0 (C), 68.3 (CH_2_), 51.4 (***C***Me_2_), 26.8 (CH_3_) and 26.5 (CH_3_); HRMS (NSI^+^): found 304.1004. C_16_H_18_NO_3_S (M + H) requires 304.1002.

#### 3.2.7. *N*-Butyl-3-(hydroxy(phenyl)methyl)thiophene-2-carboxamide **25** and 5-Butyl-4-phenyl-4,5-dihydro-6*H*-thieno[2,3-*c*]pyrrol-6-one **26**

Under a nitrogen atmosphere, *n*-butyllithium (2.5 M in hexanes, 6.6 cm^3^, 16.5 mmol) was added dropwise to a stirred solution of 3-(benzyloxy)-*N*-butylthiophene-2-carboxamide **10** (1.45 g, 5.01 mmol) in dry THF (50 cm^3^). After stirring at room temperature for 2 h, the reaction mixture was quenched by the addition of saturated aq. NH_4_Cl and extracted with Et_2_O (3 × 30 cm^3^). The combined organic extracts were washed with NaOH and water before being dried and evaporated to give **25** as a pale brown oil which was used without further purification; ν_max_/cm^–1^ 3256, 3086, 2957, 2930, 1612, 1545, 1450, 1302, 1026, 698 and 669; δ_H_ (400 MHz) 7.37–7.29 (4H, m, Ph), 7.27–7.23 (1H, m, Ph), 7.21 (1H, d, *J* 5.0, 5-H), 6.96 (1H, t, *J* 5.6, NH), 6.71 (1H, d, *J* 5.0, 4-H), 6.02 (1H, s, C***H***OH), 5.87 (1H, br s, OH), 3.30 (2H, td, *J* 7.2, 5.6, NCH_2_), 1.52–1.45 (2H, m, NCH_2_C***H***_2_), 1.35–1.26 (2H, m, C***H***_2_CH_3_) and 0.89 (3H, t, *J* 7.2, CH_3_); δ_C_ (75 MHz) 163.1 (C=O), 147.8 (C), 142.3 (C), 133.4 (C), 130.4 (CH), 128.2 (2CH), 127.3 (CH), 126.7 (CH), 126.2 (2CH), 70.9 (CHOH), 39.9 (NCH_2_), 31.3 (CH_2_), 20.0 (CH_2_) and 13.7 (CH_3_); HRMS (ESI^+^): found 312.1023. C_16_H_19_NaNO_2_S (M + Na) requires 312.1029.

A mixture of *N*-butyl-3-(hydroxy(phenyl)methyl)thiophene-2-carboxamide **25** (assuming 5.01 mmol) and *p*-toluenesulfonic acid monohydrate (1.90 g, 9.99 mmol) in toluene (50 cm^3^) was heated at reflux for 1 h. After cooling to room temperature, the reaction mixture was washed with water (50 cm^3^), 2 M NaOH (50 cm^3^) and brine (50 cm^3^) before being dried and evaporated. The crude residue was purified by filtration through a silica plug (Et_2_O) to give **26** (1.26 g, 93%) as a tan-coloured solid; mp 90–93 °C; ν_max_/cm^–1^ 2955, 1668, 1441, 1398, 1310, 1069, 781, 743, 698 and 637; δ_H_ (400 MHz) 7.55 (1H, d, *J* 4.8, 5-H), 7.38–7.32 (3H, m, Ph), 7.16–7.13 (2H, m, Ph), 6.79 (1H, d, *J* 4.8, 4-H), 5.39 (1H, s, CHPh), 3.84 (1H, dt, *J* 14.4, 7.8, NCH), 2.86–2.79 (1H, m, NCH), 1.54–1.46 (2H, m, NCH_2_C***H***_2_), 1.34–1.24 (2H, m, C***H***_2_CH_3_) and 0.88 (3H, t, *J* 7.2, CH_3_); δ_C_ (125 MHz) 164.4 (C=O), 155.7 (C), 136.0 (C), 134.8 (C), 134.5 (CH), 129.0 (2CH), 128.6 (CH), 127.3 (2CH), 120.7 (CH), 62.9 (CHPh), 40.3 (NCH_2_), 30.6 (CH_2_), 19.9 (CH_2_) and 13.7 (CH_3_); HRMS (NSI^+^): found 272.1103. C_16_H_18_NOS (M + H) requires 272.1104.

### 3.3. Preparation and Reactions of 2-Benzyloxy-3-thienyl Systems

#### 3.3.1. Attempted Cyclisation of 2-(2-(Benzyloxy)thiophen-3-yl)-4,4-dimethyl-4,5-dihydrooxazole **11**

Under a nitrogen atmosphere, *n*-butyllithium (2.5 M in hexanes, 0.66 cm^3^, 1.65 mmol) was added to a stirred mixture of 2-(2-(benzyloxy)thiophen-3-yl)-4,4-dimethyl-4,5-dihydrooxazole **11** [11] (0.1437 g, 0.50 mmol) and potassium *tert*-butoxide (0.1875 g, 1.67 mmol) in dry THF (5 cm^3^). The mixture was stirred at rt for 2 h before being quenched by the addition of saturated aq. NH_4_Cl and extracted with Et_2_O (3 × 10 cm^3^). The combined extracts were dried and evaporated to give, after purification by preparative TLC (Al_2_O_3_, Et_2_O/hexane 1:1), at R_f_ 0.50, (3-(4,4-Dimethyl-4,5-dihydrooxazol-2-yl)thiophen-2-yl)(phenyl)methanol **30** (96.7 mg, 67%) as an orange oil; ν_max_/cm^–1^ 3177, 2965, 1636, 1535, 1452, 1288, 1194, 1148, 974 and 698; δ_H_ (500 MHz) 8.04 (1H, br s, OH), 7.49 (2H, d, *J* 7.0, Ph), 7.36–7.28 (4H, m, ArH and Ph), 7.06 (1H, d, *J* 5.0, ArH), 6.10 (1H, s, C***H***OH), 4.09 and 4.06 (2H, AB pattern, *J* 6.8, CH_2_), 1.38 (3H, s, CH_3_) and 1.28 (3H, s, CH_3_); δ_C_ (125 MHz) 159.6 (C), 153.7 (C), 141.8 (C), 128.6 (CH), 127.9 (2CH), 127.7 (CH), 126.8 (2CH), 125.5 (C), 123.2 (CH), 79.0 (CH_2_), 69.5 (CHOH), 67.3 (4ry, ***C***Me_2_), 28.5 (CH_3_) and 28.1 (CH_3_); HRMS (NSI^+^): found 288.1052. C_16_H_18_NO_2_S (M + H) requires 288.1053.

#### 3.3.2. *N*-Butylthiophene-3-carboxamide **32**

Oxalyl chloride (2.0 cm^3^, 3.00 g, 23.6 mmol) was added to a solution of thiophene-3-carboxylic acid **31** (2.52 g, 19.7 mmol) in CH_2_Cl_2_ (30 cm^3^) and the mixture was stirred for 18 h. Evaporation gave thiophene-3-carbonyl chloride as a pale-yellow solid which was used immediately without further purification.

A solution of thiophene-3-carbonyl chloride (assuming 19.7 mmol) in toluene (30 cm^3^) was added dropwise to a solution of *n*-butylamine (5.8 cm^3^, 4.29 g, 58.7 mmol) in toluene (30 cm^3^) stirred at 0 °C. Once the addition was complete, the reaction mixture was allowed to warm to rt over 1 h before being poured into water. The organic layer was separated and washed with 2M NaOH and brine, dried and evaporated to give, after recrystallisation (EtOAc/hexane), **32** (2.54 g, 70%) as colourless crystals; mp 66–68 °C; (lit. [19] 53–55 °C); ν_max_/cm^–1^ 3253, 3085, 2921, 1617, 1555, 1301, 1220, 1127, 881, 831, 741 and 707; δ_H_ (500 MHz) 7.84 (1H, dd, *J* 3.0, 1.5, ArH), 7.37 (1H, dd, *J* 5.0, 1.5, ArH), 7.33 (1H, dd, *J* 5.0, 3.0, ArH), 6.02 (1H, br s, NH), 3.43 (2H, td, *J* 7.0, 6.0, NCH_2_), 1.62–1.56 (2H, m, NCH_2_C***H***_2_), 1.44–1.37 (2H, m, C***H***_2_CH_3_) and 0.95 (3H, t, *J* 7.5, CH_3_). The ^1^H NMR spectral data were in accordance with those previously reported [19]. IR data are reported for the first time.

#### 3.3.3. *N*-Butyl-2-(trimethylsilyl)thiophene-3-carboxamide **33**

Under a nitrogen atmosphere, *n*-butyllithium (2.5 M in hexane, 5.2 cm^3^, 13.0 mmol) was added dropwise to a stirred −78 °C solution of *N*-butylthiophene-3-carboxamide **32** (1.10 g, 6.00 mmol) in dry THF (30 cm^3^). After stirring at −78 °C for 5 min, the reaction mixture was allowed to warm to rt for 1 h, before being cooled to −78 °C and treated with bis(trimethylsilyl) peroxide (1.28 g, 7.18 mmol). The reaction mixture was allowed to warm to rt over 18 h before being poured into sat. aq. NH_4_Cl (100 cm^3^) and extracted with Et_2_O (3 × 50 cm^3^). The combined organic extracts were dried and evaporated and the crude residue was purified by column chromatography (SiO_2_, Et_2_O/hexane 3:2) to give, at R_f_ 0.90, **33** (0.32 g, 21%) as tan-coloured crystals; mp 86–89 °C; ν_max_/cm^–1^ 3285, 2957, 1620, 1558, 1402, 1296, 1240, 1005, 833, 746, 704 and 604; δ_H_ (500 MHz) 7.50 (1H, d, *J* 5.0, ArH), 7.29 (1H, d, *J* 5.0, ArH), 5.94 (1H, br s, NH), 3.41 (2H, td, *J* 7.0, 6.0, NCH_2_), 1.61–1.55 (2H, m, NCH_2_C***H***_2_), 1.43–1.36 (2H, m, C***H***_2_CH_3_), 0.94 (3H, t, *J* 7.5, CH_2_C***H***_3_) and 0.39 (9H, s, SiMe_3_); δ_C_ (125 MHz) 164.6 (C=O), 145.7 (C), 143.0 (C), 130.2 (CH), 126.9 (CH), 39.6 (NCH_2_), 31.7 (CH_2_), 20.1 (CH_2_), 13.8 (CH_3_) and 0.0 (SiMe_3_); HRMS (NSI^+^): found 256.1184. C_12_H_22_NOSSi (M + H) requires 256.1186.

#### 3.3.4. 5-Butyl-6-phenyl-5,6-dihydro-4*H*-thieno[2,3-*c*]pyrrol-4-one **35** and 2-Benzoyl-*N*-butylthiophene-3-carboxamide **36**

Under a nitrogen atmosphere, *n*-butyllithium (2.5 M in hexane, 4.2 cm^3^, 10.5 mmol) was added dropwise to a stirred −78 °C solution of *N*-butylthiophene-3-carboxamide **32** (0.9158 g, 5.00 mmol) in dry THF (50 cm^3^). After stirring at −78 °C for 5 min, the reaction mixture was allowed to warm to rt for 1 h before benzaldehyde (0.57 cm^3^, 0.60 g, 5.61 mmol) was added and stirring was continued for 18 h. The reaction mixture was poured into sat. aq. NH_4_Cl (100 cm^3^) and extracted with Et_2_O (3 × 50 cm^3^) and the combined organic layers were dried and evaporated.

The residue was dissolved in toluene (100 cm^3^) and treated with *p*-toluenesulfonic acid monohydrate (1.90 g, 9.99 mmol) before being heated at reflux for 1 h. After cooling to rt, the reaction mixture was washed with water (50 cm^3^), 2 M NaOH (50 cm^3^) and brine (50 cm^3^) before being dried and evaporated. The crude residue was purified by column chromatography (SiO_2_, Et_2_O/hexane 3:2) to give, at R_f_ 0.50, **35** (0.3223 g, 24%) as a pale yellow solid; mp 96–98 °C; ν_max_/cm^–1^ 2953, 1668, 1454, 1412, 1375, 1308, 1267, 1070, 760, 700 and 575; δ_H_ (400 MHz) 7.40–7.34 (4H, m, ArH), 7.27 (1H, d, *J* 4.8, ArH), 7.17–7.13 (2H, m, ArH), 5.51 (1H, s, CHPh), 3.84 (1H, dt, *J* 14.0, 8.0, NCH), 2.81 (1H, ddd, *J* 14.0, 7.6, 6.4, NC), 1.53–1.45 (2H, m, NCH_2_C***H***_2_), 1.35–1.24 (2H, m, C***H***_2_CH_3_) and 0.88 (3H, t, *J* 7.4, CH_3_); δ_C_ (125 MHz) 165.1 (C=O), 155.0 (C), 139.8 (C), 136.7 (C), 130.2 (CH), 129.2 (2CH), 129.0 (CH), 127.3 (2CH), 120.1 (CH), 62.9 (CHPh), 40.3 (NCH_2_), 30.7 (CH_2_), 20.0 (CH_2_) and 13.7 (CH_3_); HRMS (ASAP^+^): found 272.1115. C_16_H_18_NOS (M + H) requires 272.1104.

This was followed by a second fraction to give, at R_f_ 0.30, **36** (0.1817 g, 13%) as an orange oil; ν_max_/cm^–1^ 3277, 2957, 2870, 1630, 1549, 1449, 1406, 1279, 847 and 691; δ_H_ (400 MHz) 8.65 (1H, br s, NH), 7.84–7.81 (2H, m, Ph), 7.75 (1H, d, *J* 5.0, ArH), 7.64–7.60 (1H, m, Ph), 7.55 (1H, d, *J* 5.0, ArH), 7.51–7.46 (2H, m, Ph), 3.33 (2H, td, *J* 7.2, 5.6, NCH_2_), 1.56–1.49 (2H, m, NCH_2_C***H***_2_), 1.42–1.33 (2H, m, C***H***_2_CH_3_) and 0.92 (3H, t, *J* 7.4, CH_3_); δ_C_ (100 MHz, signals for major amide rotamer only) 190.6 (COPh), 162.5 (CONHBu), 142.6 (C), 138.8 (C), 138.1 (C), 133.3 (CH), 132.8 (CH), 130.4 (CH), 129.8 (2CH), 128.3 (2CH), 39.7 (NCH_2_), 31.2 (CH_2_), 20.2 (CH_2_) and 13.7 (CH_3_); HRMS (NSI^+^): found 288.1052. C_16_H_18_NO_2_S (M + H) requires 288.1053.

### 3.4. Preparation and Reactions of 4-Benzyloxy-3-thienyl Systems

#### 3.4.1. Methyl 3-((2-Methoxy-2-oxoethyl)thio)propanoate **37**

Following a literature procedure [12], methyl acrylate (18.37 g, 0.213 mol) was added dropwise to a stirred mixture of methyl thioglycolate (21.18 g, 0.200 mol) and piperidine (0.2 cm^3^, 0.17 g, 2.02 mmol). Once approximately half of the methyl acrylate had been added, further piperidine (0.2 cm^3^, 0.17 g, 2.02 mmol) was added. Once the addition was complete, the reaction mixture was heated at 80 °C for 30 min. After cooling to rt, the reaction mixture was diluted with Et_2_O (150 cm^3^) and washed with water (5 × 50 cm^3^) before being dried and evaporated to give **37** (37.12 g, 97%) as a pale yellow oil which was used without further purification; δ_H_ (400 MHz) 3.75 (3H, s, CH_3_), 3.71 (3H, s, CH_3_), 3.26 (2H, s, MeO_2_CCH_2_S), 2.92 (2H, t, *J* 7.2, SC***H***_2_CH_2_CO_2_Me) and 2.66 (2H, t, *J* 7.2, SCH_2_C***H***_2_CO_2_Me); δ_C_ (100 MHz) 172.0 (C=O), 170.7 (C=O), 52.4 (CH_3_), 51.8 (CH_3_), 34.1 (CH_2_), 33.4 (CH_2_) and 27.5 (CH_2_). The ^1^H NMR spectral data were in accordance with those previously reported [11]. ^13^C NMR data are reported for the first time.

#### 3.4.2. Methyl 4-Oxotetrahydrothiophene-3-carboxylate **38**

Following a literature procedure [13], sodium methoxide was prepared by the addition of sodium (12.51 g, 0.544 mol) in small portions to methanol (90 cm^3^). Once the sodium had fully dissolved, a solution of methyl 3-((2-methoxy-2-oxoethyl)thio)propanoate **37** (37.12 g, 0.193 mol) in methanol (30 cm^3^) was added dropwise and the reaction mixture was heated at reflux for 1 h. After cooling to rt, the reaction mixture was poured into a mixture of crushed ice (400 g) and conc. HCl (100 cm^3^) before being extracted with CH_2_Cl_2_ (2 × 300 cm^3^). The combined organic layers were washed with sat. aq. NaHCO_3_ (250 cm^3^) before being dried and evaporated. The crude residue was purified by distillation to give **38** (11.69 g, 38%) as a colourless oil which partially crystallised on standing; bp 103 °C/4.9 Torr; (lit. [20] 109 °C/4 Torr).

#### 3.4.3. Methyl 4-Hydroxythiophene-3-carboxylate **39**

Following a literature procedure [14], sulfuryl chloride (9.7 cm^3^, 16.15 g, 0.120 mol) was added dropwise to a stirred 0 °C solution of methyl 4-oxotetrahydrothiophene-3-carboxylate **973** (17.41 g, 0.109 mol) in CH_2_Cl_2_ (110 cm^3^) over a period of 1 h. The reaction mixture was stirred at 0 °C for 30 min before being washed with sat. aq. NaHCO_3_ (150 cm^3^) and water (3 × 50 cm^3^). The organic layer was dried and evaporated to give after filtration through a silica plug (Et_2_O), **39** (12.93 g, 75%) as a light brown low-melting solid; δ_H_ (300 MHz) 8.72 (1H, s, OH), 7.90 (1H, d, *J* 3.6, ArH), 6.39 (1H, d, *J* 3.6, ArH) and 3.92 (3H, s, CH_3_); δ_C_ (125 MHz) 165.4 (C=O), 155.3 (C–O), 131.0 (CH), 119.0 (C), 100.0 (CH) and 51.8 (CH_3_). The ^1^H NMR spectral data were in accordance with those previously reported [15]. ^13^C NMR data are reported for the first time.

#### 3.4.4. Methyl 4-Acetoxythiophene-3-carboxylate **40**

Following a literature procedure [15], a mixture of methyl 4-oxotetrahydrothiophene-3-carboxylate **38** (30.80 g, 0.192 mol) and *p*-toluenesulfonic acid monohydrate (0.19 g, 1.00 mmol) in isopropenyl acetate (70 cm^3^) was heated at reflux for 18 h. After cooling to rt, the reaction mixture was concentrated *in vacuo* to give methyl 4-acetoxy-2,5-dihydrothiophene-3-carboxylate as a dark brown oil which was used without further purification.

Following a literature procedure [15], sulfuryl chloride (19.5 cm^3^, 32.47 g, 0.241 mol) was added dropwise to a stirred −25 °C solution of methyl 4-acetoxy-2,5-dihydrothiophene-3-carboxylate (assuming 0.192 mol) in CH_2_Cl_2_ (80 cm^3^) over a period of 1 h. The reaction mixture was allowed to warm to rt for 18 h before being evaporated to give **40** (19.10 g, 50%) as a dark brown oil which was used without further purification; δ_H_ (500 MHz) 8.07 (1H, d, *J* 3.8, ArH), 6.98 (1H, d, *J* 3.8, ArH), 3.83 (3H, s, OCH_3_) and 2.33 (3H, s, COCH_3_). The ^1^H NMR spectral data were in accordance with those previously reported [15].

#### 3.4.5. Silver(I) Oxide

A solution of sodium hydroxide (14.61 g, 0.365 mol) in water (440 cm^3^) was added dropwise to a stirred solution of silver nitrate (60.00 g, 0.353 mol) in water (110 cm^3^). Once the addition was complete, the precipitate was collected by filtration and washed with water until the washings were neutral before being dried *in vacuo* to give the title compound (39.60 g, 97%) as a brown solid which was stored in the dark and used without further purification.

#### 3.4.6. Methyl 4-(Benzyloxy)thiophene-3-carboxylate **41**

A mixture of methyl 4-hydroxythiophene-3-carboxylate **39** (12.91 g, 81.6 mmol), silver(I) oxide (28.40 g, 0.123 mol) and benzyl bromide (10.7 cm^3^, 15.39 g, 90.0 mmol) in CH_2_Cl_2_ (500 cm^3^) was heated at reflux for 3 d. After cooling to rt, the reaction mixture was filtered and evaporated and the crude residue was purified by column chromatography (SiO_2_, Et_2_O/hexane 1:1) to give, at R_f_ 0.90, **983** (5.47 g, 27%) as a red oil; ν_max_/cm^–1^ 3113, 2949, 1726, 1541, 1449, 1265, 1082, 770 and 698; δ_H_ (500 MHz) 8.01 (1H, d, *J* 3.5, ArH), 7.48 (2H, d, *J* 7.5, Ph), 7.38 (2H, t, *J* 7.5, Ph), 7.31 (1H, t, *J* 7.5, Ph), 6.31 (1H, d, *J* 3.5, ArH), 5.13 (2H, s, CH_2_) and 3.86 (3H, s, CH_3_); δ_C_ (125 MHz) 162.2 (C=O), 156.1 (C–O), 136.5 (C), 133.2 (CH), 128.5 (2CH), 127.8 (CH), 126.8 (2CH), 123.7 (C), 99.9 (CH), 72.3 (CH_2_) and 51.6 (CH_3_); HRMS (ESI^+^): found 271.0393. C_13_H_12_NaO_3_S (M + Na) requires 271.0399.

#### 3.4.7. 4-(Benzyloxy)thiophene-3-carboxylic Acid **42**

A mixture of methyl 4-(benzyloxy)thiophene-3-carboxylate **41** (5.17 g, 20.8 mmol) and sodium hydroxide (1.74 g, 43.5 mmol) in water (45 cm^3^) was heated at reflux for 18 h. After cooling to rt, the reaction mixture was washed with CH_2_Cl_2_ (30 cm^3^) before being adjusted to pH 1 by the addition of 2 M HCl and extracted with CH_2_Cl_2_ (2 × 50 cm^3^). The combined organic extracts were dried and evaporated to give, after recrystallisation (PhMe), **42** (3.08 g, 63%) as brown crystals; mp 101–105 °C; ν_max_/cm^–1^ 3123, 1667, 1537, 1447, 1285, 1196, 1088, 880 and 764; δ_H_ (500 MHz) 9.96 (1H, br s, CO_2_H), 8.21 (1H, d, *J* 3.5, ArH), 7.45–7.37 (5H, m, Ph), 6.48 (1H, d, *J* 3.5, ArH) and 5.20 (2H, s, CH_2_); δ_C_ (125 MHz) 164.7 (C=O), 155.0 (C–O), 135.5 (C), 135.2 (CH), 128.6 (2CH), 128.3 CH), 127.2 (2CH), 122.7 (C), 100.4 (CH) and 72.9 (CH_2_); HRMS (ESI^+^): found 257.0239. C_12_H_10_NaO_3_S (M + Na) requires 257.0243.

#### 3.4.8. 4-(Benzyloxy)-*N*-(1-hydroxy-2-methylpropan-2-yl)thiophene-3-carboxamide **43**

Thionyl chloride (0.31 cm^3^, 0.51 g, 4.25 mmol) was added to a suspension of 4-(benzyloxy)thiophene-3-carboxylic acid **42** (0.50 g, 2.13 mmol) in toluene (5 cm^3^) and the mixture was heated under reflux for 3 h. After cooling to rt, the mixture was evaporated to give 4-(benzyloxy)thiophene-3-carbonyl chloride as a red oil which was used immediately without further purification.

A solution of 4-(benzyloxy)thiophene-3-carbonyl chloride (assuming 2.13 mmol) in CH_2_Cl_2_ (30 cm^3^) was added dropwise to a solution of 2-amino-2-methylpropan-1-ol (0.41 g, 4.60 mmol) in CH_2_Cl_2_ (10 cm^3^) stirred at 0 °C. After the addition, the mixture was allowed to warm to rt and stirred for 18 h before being poured into water. The organic layer was separated and the aqueous layer extracted with CH_2_Cl_2_ (2 × 20 cm^3^). The combined organic layers were washed successively with 2M HCl, 2M NaOH and water before being dried and evaporated to give **43** (0.60 g, 92%) as a pale-yellow solid which was used without further purification; mp 90–92 °C; ν_max_/cm^–1^ 3366, 2970, 1628, 1560, 1435, 1364, 1310, 1074, 976 and 606; δ_H_ (400 MHz) 8.07 (1H, d, *J* 3.6, ArH), 7.65 (1H, br s, NH), 7.46–7.37 (5H, m, Ph), 6.46 (1H, d, *J* 3.6, ArH), 5.22 (1H, br s, OH), 5.08 (2H, s, OCH_2_Ph), 3.57 (2H, s, CH_2_OH) and 1.16 (6H, s, CH_3_); δ_C_ (100 MHz) 162.1 (C=O), 153.1 (C–O), 135.1 (C), 131.9 (CH), 128.8 (CH), 128.7 (2CH), 128.1 (2CH), 126.6 (C), 99.7 (CH), 73.2 (CH_2_), 70.7 (CH_2_), 56.0 (***C***Me_2_) and 24.5 (2CH_3_); HRMS (NSI^+^): found 306.1150. C_16_H_20_NO_3_S (M + H) requires 306.1158.

#### 3.4.9. 2-(4-(Benzyloxy)thiophen-3-yl)-4,4-dimethyl-4,5-dihydrooxazole **13**

Thionyl chloride (0.15 cm^3^, 0.24 g, 2.06 mmol) was added to a solution of 4-(benzyloxy)-*N*-(1-hydroxy-2-methylpropan-2-yl)thiophene-3-carboxamide **43** (0.50 g, 1.64 mmol) in CH_2_Cl_2_ (20 cm^3^) and the mixture was stirred at rt for 18 h. The mixture was washed with 2M NaOH and water before being dried and evaporated to give, after purification by column chromatography (SiO_2_, Et_2_O/hexane 3:2), at R_f_ 0.40, **13** (0.41 g, 87%) as a pale yellow solid; mp 77–80 °C; ν_max_/cm^–1^ 2965, 1651, 1535, 1449, 1371, 1211, 1194, 1042, 733 and 698; δ_H_ (400 MHz) 7.79 (1H, d, *J* 3.6, ArH), 7.47 (2H, d, *J* 7.6, Ph), 7.36 (2H, t, *J* 7.4, Ph), 7.29 (1H, t, *J* 7.2, Ph), 6.30 (1H, d, *J* 3.6, ArH), 5.16 (2H, s, OCH_2_Ph), 4.05 (2H, s, OCH_2_) and 1.39 (6H, s, CH_3_); δ_C_ (100 MHz) 157.2 (C), 155.3 (C), 136.9 (C), 129.1 (CH), 128.4 (2CH), 127.6 CH), 126.7 (2CH), 121.6 (C), 100.3 (CH), 78.5 (OCH_2_), 72.4 (OCH_2_), 67.5 (***C***Me_2_) and 28.4 (2CH_3_); HRMS (ESI^+^): found 288.1047. C_16_H_18_NO_2_S (M + H) requires 288.1053.

#### 3.4.10. 4-((1-Hydroxy-2-methylpropan-2-yl)carbamoyl)thiophen-3-yl Benzoate **45**

Under a nitrogen atmosphere, *n*-butyllithium (2.5 M in hexanes, 0.88 cm^3^, 2.20 mmol) was added to a stirred mixture of 2-(4-(benzyloxy)thiophen-3-yl)-4,4-dimethyl-4,5-dihydrooxazole **13** (0.1443 g, 0.50 mmol) and potassium *tert*-butoxide (0.2485 g, 2.21 mmol) in dry THF (5 cm^3^). The mixture was stirred at rt for 2 h before being quenched by the addition of saturated aq. NH_4_Cl and extracted with Et_2_O (3 × 10 cm^3^). The combined extracts were dried and evaporated to give, after purification by preparative TLC (Al_2_O_3_, Et_2_O/hexane 1:1), at R_f_ 0.35, **45** (55.8 mg, 35%) in slightly impure form as a brown oil; ν_max_/cm^–1^ 3335, 2972, 1744, 1638, 1545, 1450, 1246, 1177, 1047, 908, 766 and 700; δ_H_ (400 MHz) 8.19–8.16 (2H, m, Ph), 7.99 (1H, d, *J* 3.6, ArH), 7.71–7.67 (1H, m, Ph), 7.55 (2H, t, *J* 7.8, Ph), 7.34 (1H, d, *J* 3.6, ArH), 6.61 (1H, br s, NH), 4.58 (1H, br s, OH), 3.59 (2H, s, CH_2_) and 1.24 (6H, s, CH_3_); δ_C_ (125 MHz) 163.7 (CO), 162.2 (CO), 143.2 (C), 134.4 (CH), 130.0 (2CH), 129.9 (CH), 129.3 (C), 128.9 (2CH), 128.4 (C), 113.7 (CH), 69.9 (OCH_2_), 56.3 (***C***Me_2_) and 24.6 (2CH_3_); HRMS (NSI^+^): found 320.0953. C_16_H_18_NO_4_S (M + H) requires 320.0951.

#### 3.4.11. 4-(Benzyloxy)-*N*-butylthiophene-3-carboxamide **14**

Thionyl chloride (1.0 cm^3^, 1.63 g, 13.7 mmol) was added to a suspension of 4-(benzyloxy)thiophene-3-carboxylic acid **42** (1.50 g, 6.40 mmol) in toluene (15 cm^3^) and the mixture was heated under reflux for 3 h. After cooling to rt, the mixture was evaporated to give 4-(benzyloxy)thiophene-3-carbonyl chloride as a red oil which was used immediately without further purification.

A solution of 4-(benzyloxy)thiophene-3-carbonyl chloride (assuming 6.40 mmol) in toluene (30 cm^3^) was added dropwise to a solution of *n*-butylamine (1.9 cm^3^, 1.41 g, 19.2 mmol) in toluene (10 cm^3^) stirred at 0 °C. Once the addition was complete, the reaction mixture was allowed to warm to rt over 1 h before being poured into water. The organic layer was separated and washed with 2M NaOH and brine, dried and evaporated to give, after purification by column chromatography (SiO_2_, Et_2_O/hexane 3:2), at R_f_ 0.50, **14** (1.24 g, 67%) as a tan-coloured solid; mp 51–53 °C; ν_max_/cm^–1^ 3385, 3111, 2970, 1630, 1557, 1435, 1364, 1265, 1184, 1074, 988, 714 and 579; δ_H_ (400 MHz) 8.10 (1H, d, *J* 3.6, ArH), 7.45–7.37 (6H, m, NH and Ph), 6.43 (1H, d, *J* 3.6, ArH), 5.10 (2H, s, OCH_2_), 3.34 (2H, td, *J* 6.8, 5.6, NCH_2_), 1.45–1.38 (2H, m, NCH_2_C***H***_2_), 1.25–1.15 (2H, m, C***H***_2_CH_3_) and 0.81 (3H, t, *J* 7.2, CH_3_); δ_C_ (100 MHz) 161.5 (C=O), 153.5 (C–O), 135.5 (C), 131.5 (CH), 128.8 (2CH), 128.7 (CH), 127.9 (2CH), 127.1 (C), 99.5 (CH), 73.1 (OCH_2_), 38.7 (NCH_2_), 31.3 (CH_2_), 20.0 (CH_2_) and 13.7 (CH_3_); HRMS (NSI^+^): found 290.1207. C_16_H_20_NO_2_S (M + H) requires 290.1209.

#### 3.4.12. Attempted [1,2]-Wittig Rearrangement of 4-(Benzyloxy)-*N*-butylthiophene-3-carboxamide **14**

Under a nitrogen atmosphere, *n*-butyllithium (2.5 M, 2.7 cm^3^, 6.75 mmol) was added dropwise to a stirred solution of 4-(benzyloxy)-*N*-butylthiophene-3-carboxamide **14** (0.5787 g, 2.00 mmol) in dry THF (20 cm^3^). After stirring at rt for 2 h, the reaction mixture was quenched by the addition of saturated aq. NH_4_Cl and extracted with Et_2_O (3 × 30 cm^3^). The combined organic extracts were washed with NaOH and water before being dried and evaporated to give, after purification by column chromatography (SiO_2_, Et_2_O/hexane 3:2), at R_f_ 0.65, 4-(benzyloxy)-*N*-butyl-2-(hydroxy(phenyl)methyl)thiophene-3-carboxamide **46** (27.8 mg, 4%) in slightly impure form as a tan-coloured solid; mp 74–76 °C; ν_max_/cm^–1^ 3393, 3084, 2930, 2870, 1624, 1557, 1443, 1217, 1173, 1016 and 696; δ_H_ (400 MHz) 7.73 (1H, t, *J* 5.0, NH), 7.52–7.48 (2H, m, Ph), 7.44–7.30 (8H, m, Ph), 6.77 (1H, br s, OH), 6.26 (1H, s, ArH), 6.21 (1H, s, C***H***OH), 5.04 (2H, s, OCH_2_), 3.41–3.25 (2H, m, NCH_2_), 1.42–1.35 (2H, m, NCH_2_C***H***_2_), 1.21–1.12 (2H, m, C***H***_2_CH_3_) and 0.80 (3H, t, *J* 7.2, CH_3_); δ_C_ (100 MHz) 163.6 (C=O), 155.9 (C), 154.3 (C), 140.9 (C), 135.2 (C), 128.8 (3CH), 128.03 (2CH), 128.01 (2CH), 127.97 (CH), 127.3 (2CH), 121.9 (C), 97.5 (CH), 73.0 (OCH_2_), 70.8 (CHOH), 39.0 (NCH_2_), 31.0 (CH_2_), 19.9 (CH_2_) and 13.7 (CH_3_); HRMS (ESI^+^): found 418.1439. C_23_H_25_NaNO_3_S (M + Na) requires 418.1447.

This was followed by a second fraction at R_f_ 0.50 which was further purified by preparative TLC (SiO_2_, CH_2_Cl_2_) to give, at R_f_ 0.35 *N*-butyl-4-hydroxythiophene-3-carboxamide **47** (30.9 mg, 8%) in slightly impure form as a brown oil; ν_max_/cm^–1^ 3327, 2957, 2930, 1634, 1557, 1441, 1273, 739 and 698; ^1^H NMR revealed a 3:2 mixture of enol and keto tautomers; δ_H_ (500 MHz, enol tautomer **47**) 10.36 (1H, br s, OH), 7.54 (1H, d, *J* 3.3, ArH), 6.36 (1H, d, *J* 3.3, ArH), 6.35 (1H, br s, NH), 3.41 (2H, td, *J* 7.3, 6.0, NCH_2_), 1.62–1.51 (2H, m, NCH_2_C***H***_2_), 1.44–1.33 (2H, m, C***H***_2_CH_3_) and 0.95 (3H, t, *J* 7.5, CH_3_); δ_C_ (125 MHz, enol tautomer **47**) 165.1 (C=O), 156.1 (C–O), 124.6 (CH), 121.7 (C), 100.1 (CH), 39.1 (NCH_2_), 31.6 (CH_2_), 20.08 (CH_2_) and 13.7 (CH_3_); δ_H_ (500 MHz, keto tautomer **47a**) 9.33 (1H, s, CH), 8.06 (1H, br s, NH), 3.89 (2H, s, SCH_2_), 3.36 (2H, td, *J* 7.0, 6.0, NCH_2_), 1.62–1.51 (2H, m, NCH_2_C***H***_2_), 1.44–1.33 (2H, m, C***H***_2_CH_3_) and 0.93 (3H, t, *J* 7.3, CH_3_); δ_C_ (125 MHz, keto tautomer **47a**) 199.9 (C=O), 174.7 (CH), 159.9 (CONH), 128.2 (C), 42.7 (SCH_2_), 38.7 (NCH_2_), 31.5 (CH_2_), 20.11 (CH_2_) and 13.7 (CH_3_); HRMS (NSI^+^): found 200.0740. C_9_H_14_NO_2_S (M + H) requires 200.0740. 

### 3.5. X-ray Structure Determination

Data have been deposited at the Cambridge Crystallographic Data Centre as CCDC 2111424 (**9**), 2111425 (**20**), 2111426 (**11**), 2111427 (**26**) and 2111428 (**13**). The data can be obtained free of charge from the Cambridge Crystallographic Data Centre via http://www.ccdc.cam.ac.uk/getstructures. In all cases, data were collected on a Rigaku XtaLAB 200 diffractometer using graphite monochromated Mo-Kα radiation, λ = 0.71075 Å and the structures were solved by direct methods and refined by full-matrix least-squares against F2 (SHELXL Version 2014/7 [21]).

Compound **20**

Slow evaporation of an acetone/CH_2_Cl_2_ solution gave tan-coloured crystals suitable for X-ray structure determination. Crystal data for **20**: 2C_16_H_17_NO_3_S•CH_2_Cl_2_•Me_2_CO, M = 749.76, yellow prism, crystal dimensions 0.10 × 0.10 × 0.10 mm, monoclinic, space group P2_1_/n, a = 17.4520, b = 9.9840, c = 21.1050 Å, β = 92.3760°, V = 3674.1899 Å^3^, Z = 4, D_c_ = 1.355 Mg m^–3^, T = 93 K, R = 0.0929, R_W_ = 0.2464 for 4473 reflections with I > 2σ(I) and 458 variables.

Compound **26**

Slow evaporation of a CH_2_Cl_2_ solution gave crystals suitable for X-ray structure determination. Crystal data for **26**: C_16_H_17_NOS, M = 271.38, colourless needle, crystal dimensions 0.12 × 0.02 × 0.02 mm, monoclinic, space group P2_1_, a = 8.237(3), b = 5.717(2), c = 15.097(6) Å, β = 90.539(10)°, V = 710.9(5) Å^3^, Z = 2, D_c_ = 1.268 Mg m^–3^, T = 93 K, R = 0.0481, R_W_ = 0.1086 for 2072 reflections with I > 2σ(I) and 172 variables.

Compound **9**

Slow evaporation of an MeCN solution gave crystals suitable for X-ray structure determination. Crystal data for **9**: C_16_H_17_NO_2_S, M = 287.38, colourless plate, crystal dimensions 0.10 × 0.10 × 0.01 mm, monoclinic, space group P2_1_/c, a = 15.9970(19), b = 7.5839(6), c = 12.4523(15) Å, β = 111.010(14)°, V = 1410.3(3) Å^3^, Z = 4, D_c_ = 1.353 Mg m^–3^, T = 93 K, R = 0.0584, R_W_ = 0.1406 for 2462 reflections with I > 2σ(I) and 181 variables.

Compound **11**

Slow evaporation of a CH_2_Cl_2_ solution gave crystals suitable for X-ray structure determination. Crystal data for **11**: C_16_H_17_NO_2_S, M = 287.38, colourless prism, crystal dimensions 0.12 × 0.10 × 0.06 mm, monoclinic, space group P2_1_/c, a = 14.8176(4), b = 8.72450(17), c = 11.9720(3) Å, β = 113.314(3)°, V = 1421.32(7) Å^3^, Z = 4, D_c_ = 1.343 Mg m^–3^, T = 93 K, R = 0.0274, R_W_ = 0.0730 for 2906 reflections with I > 2σ(I) and 181 variables.

Compound **13**

Slow evaporation of a toluene solution gave crystals suitable for X-ray structure determination. Crystal data for **13**: C_16_H_17_NO_2_S, M = 287.38, colourless plate, crystal dimensions 0.20 × 0.20 × 0.01 mm, monoclinic, space group P2_1_/c, a = 15.6319(4), b = 8.7152(3), c = 10.7572(3) Å, β = 93.909(3)°, V = 1462.10(7) Å^3^, Z = 4, D_c_ = 1.305 Mg m^–3^, T = 296 K, R = 0.0333, R_W_ = 0.0888 for 2781 reflections with I > 2σ(I) and 181 variables.

## 4. Conclusions

The three isomeric thienyloxazolines showed a varied and interesting pattern of reactivity with two of them undergoing Wittig rearrangement and two giving products derived from ring cleavage of an intermediate 3-aminothienofuran. One of the corresponding thienyl amides also underwent Wittig rearrangement and cyclisation of the product, as well as an isomeric one obtained by other means, gave two isomeric thienopyrrolones. The pattern of reactivity in the oxazoline series correlates well with the molecular geometry in the solid state as determined by X-ray diffraction, and the use of this method to explain patterns of competing reactivity between closely similar molecules may be useful more generally.

## Figures and Tables

**Table 1:** Hydrogen bonding parameters for **20** (Å, °).

D—H…A	D—H	H…A	D…A	D—H…A
O(7)–H(7)…O(23)	0.98(7)	1.61(7)	2.592(6)	177(7)
N(9)–H(9)…O(3)	0.98(6)	1.74(7)	2.649(6)	153(7)
O(27)–H(27)…O(3)	0.98(5)	1.72(5)	2.694(6)	173(6)
N(29)–H(29)…O(23)	0.98(5)	1.70(5)	2.584(6)	147(5)

## Data Availability

The X-ray data is deposited at CCDC as stated above and all NMR spectroscopic data is in the Supplementary Material.

## Supplementary Materials

The following are available online, Figures S1–S41: NMR spectra of new compounds. Cif and check-cif files for X-ray structures of **9**, **11**, **13**, **20** and **26**.
