# Supplementary material for: Rationalisation of Patterns of Competing Reactivity by X-ray Structure Determination: Reaction of Isomeric (Benzyloxythienyl)oxazolines with a Base
Source: Molecules. 2021 Dec 20;26(24):7690. doi: 10.3390/molecules26247690 (PMC8705950; doi:10.3390/molecules26247690)

Rationalisation of Patterns of Competing Reactivity by X-Ray Stucture Determination: Reaction of Isomeric (Benzyloxythienyl)oxazolines with Base

R. Alan Aitken*, Andrew D. Harper and Alexandra M. Z. Slawin

EaStCHEM School of Chemistry, University of St Andrews, North Haugh, St Andrews, Fife, KY16 9ST, UK.

Supplementary Material	Figure
DEPTQ ^{13}C NMR spectrum of **16**	S1
DEPTQ ^{13}C NMR spectrum of **17**	S2
^{1}H NMR and DEPTQ ^{13}C NMR spectra of **18**	S3, S4
^{1}H NMR and DEPTQ ^{13}C NMR spectra of **9**	S5, S6
DEPTQ ^{13}C NMR spectrum of **19**	S7
^{1}H NMR and DEPTQ ^{13}C NMR spectra of **20**	S8, S9
^{1}H NMR and DEPTQ ^{13}C NMR spectra of **10**	S10, S11
^{1}H NMR and DEPTQ ^{13}C NMR spectra of **25**	S12, S13
^{1}H NMR and DEPTQ ^{13}C NMR spectra of **26**	S14, S15
^{1}H NMR and DEPTQ ^{13}C NMR spectra of **30**	S16, S17
^{1}H NMR and DEPTQ ^{13}C NMR spectra of **33**	S18, S19
^{1}H NMR and DEPTQ ^{13}C NMR spectra of **35**	S20, S21
^{1}H NMR and DEPTQ ^{13}C NMR spectra of **36**	S22, S23
DEPTQ ^{13}C NMR spectrum of **37**	S24
DEPTQ ^{13}C NMR spectrum of **39**	S25

^{1}H NMR and DEPTQ ^{13}C NMR spectra of **41**	S26, S27
^{1}H NMR and DEPTQ ^{13}C NMR spectra of **42**	S28, S29
^{1}H NMR and DEPTQ ^{13}C NMR spectra of **43**	S30, S31
^{1}H NMR and DEPTQ ^{13}C NMR spectra of **13**	S32, S33
^{1}H NMR and DEPTQ ^{13}C NMR spectra of **45**	S34, S35
^{1}H NMR and DEPTQ ^{13}C NMR spectra of **18**	S36, S37
^{1}H NMR and DEPTQ ^{13}C NMR spectra of **46**	S38, S39
^{1}H NMR and DEPTQ ^{13}C NMR spectra of **47**	S40, S41

Figure S1. 125 MHz DEPTQ ^{13}C NMR spectrum of **16**

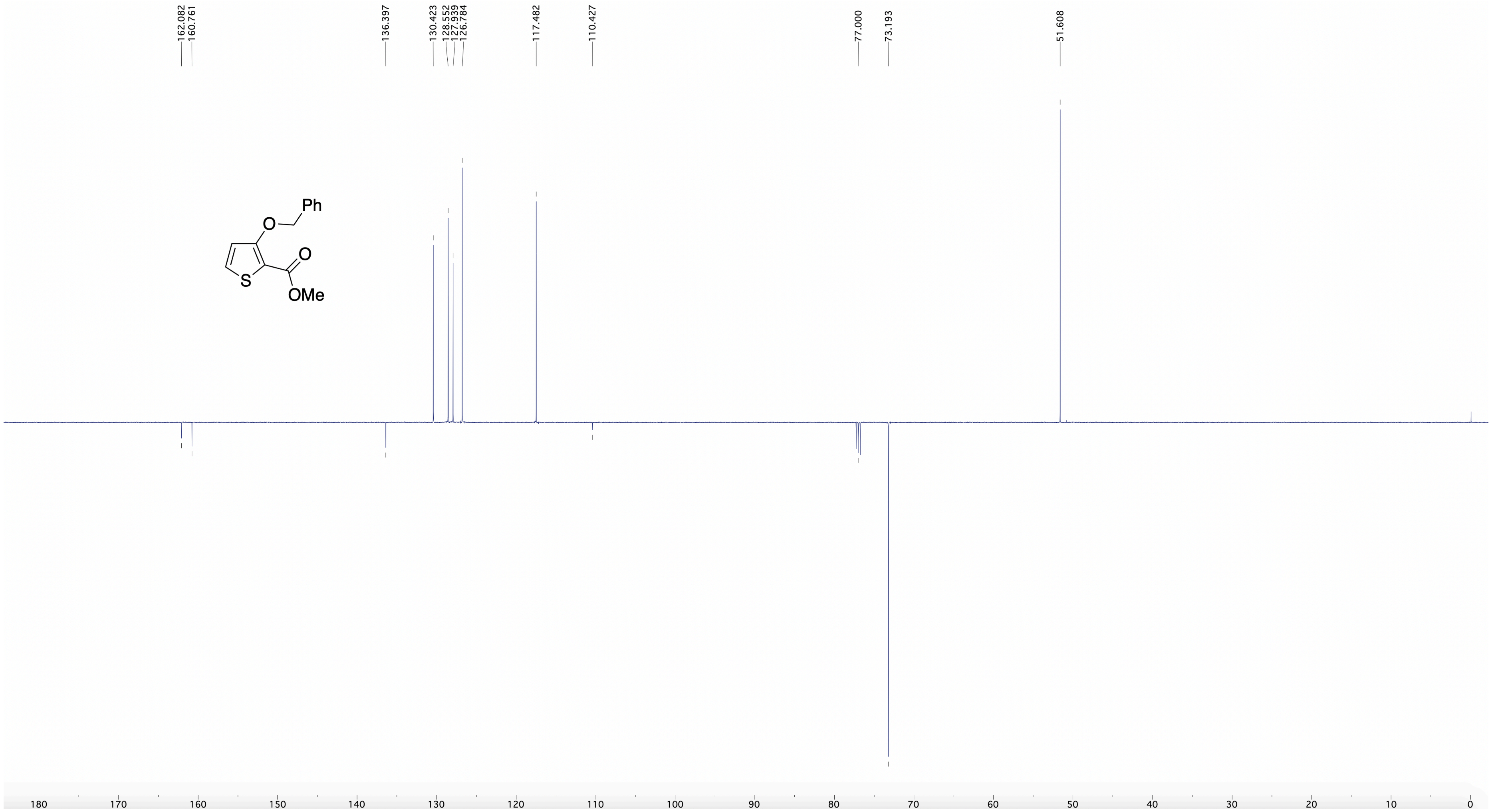

Figure S2. 100 MHz DEPTQ ^{13}C NMR spectrum of **17**

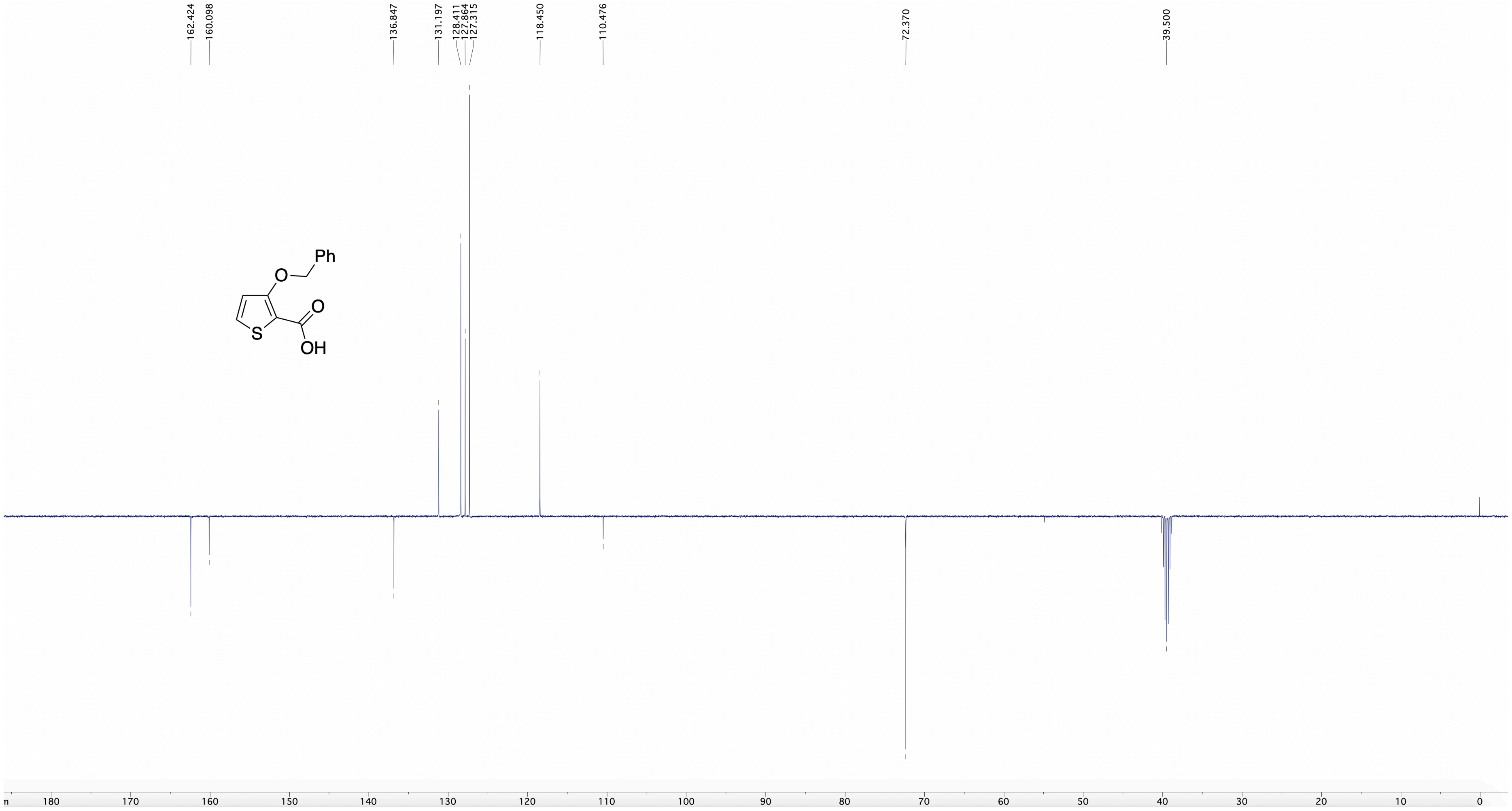

Figure S3. 500 MHz ^{1}H NMR spectrum of **18**

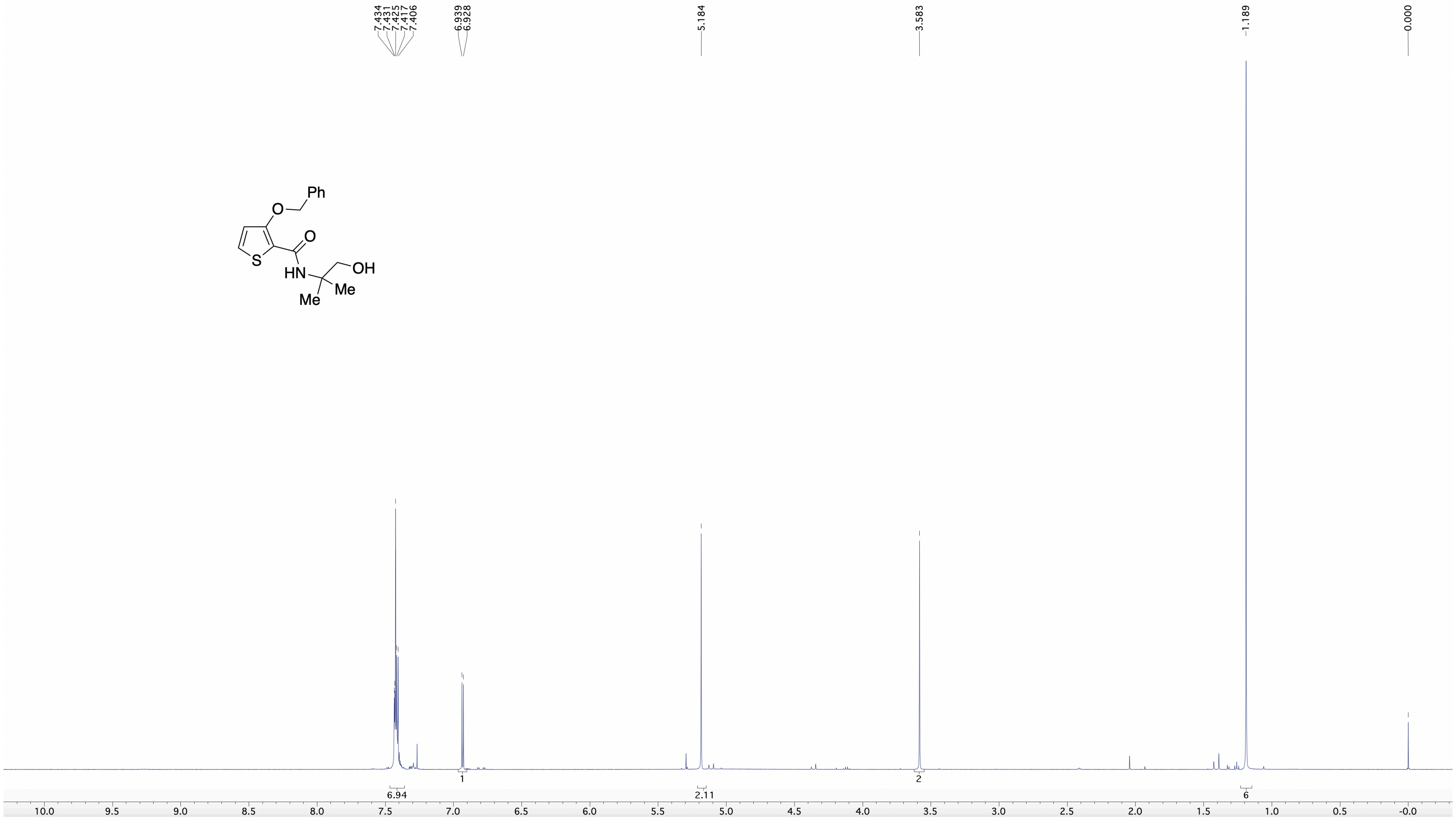

Figure S4. 125 MHz DEPTQ ^{13}C NMR spectrum of **18**

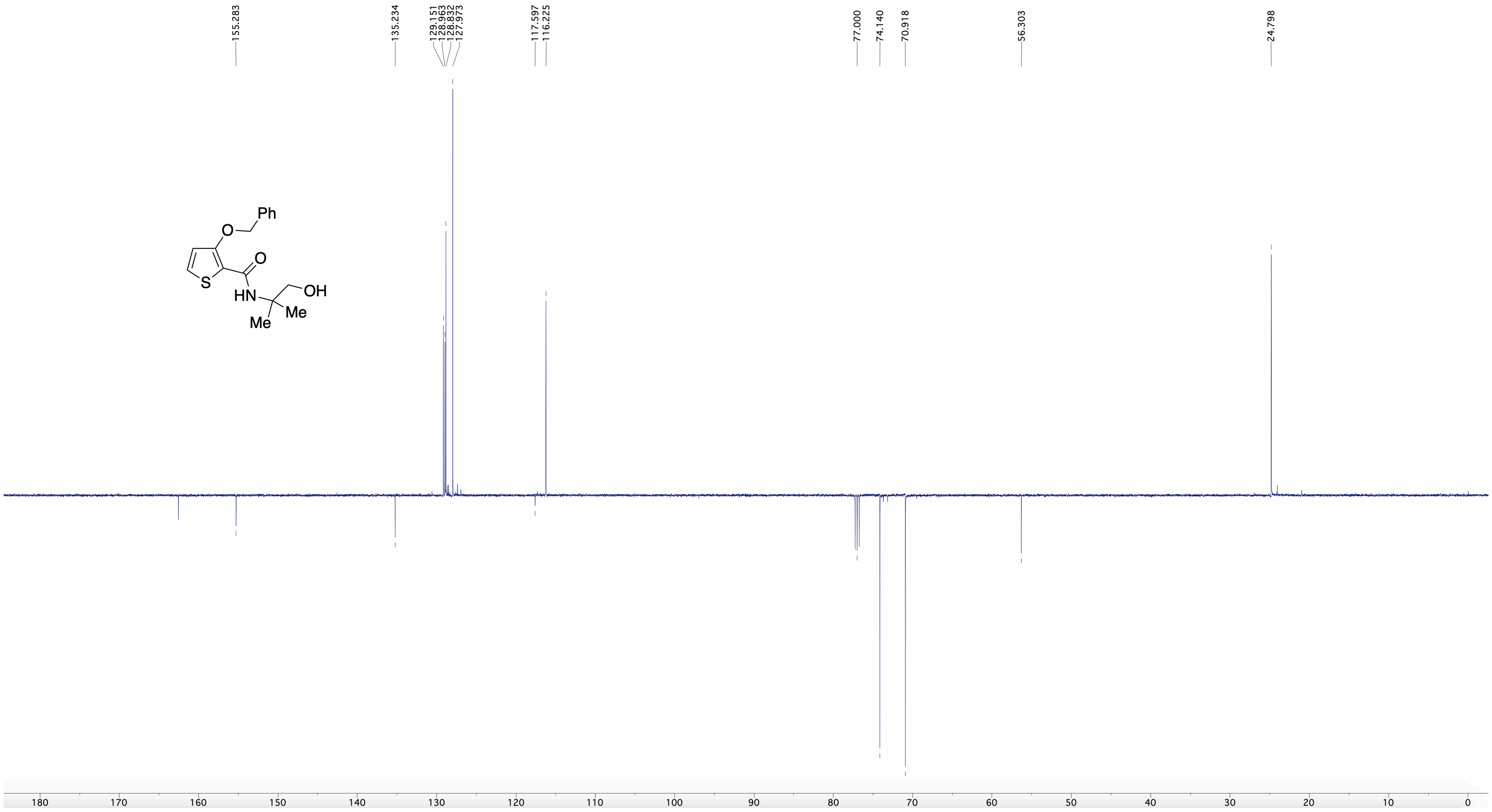

Figure S5. 500 MHz ^{1}H NMR spectrum of **9**

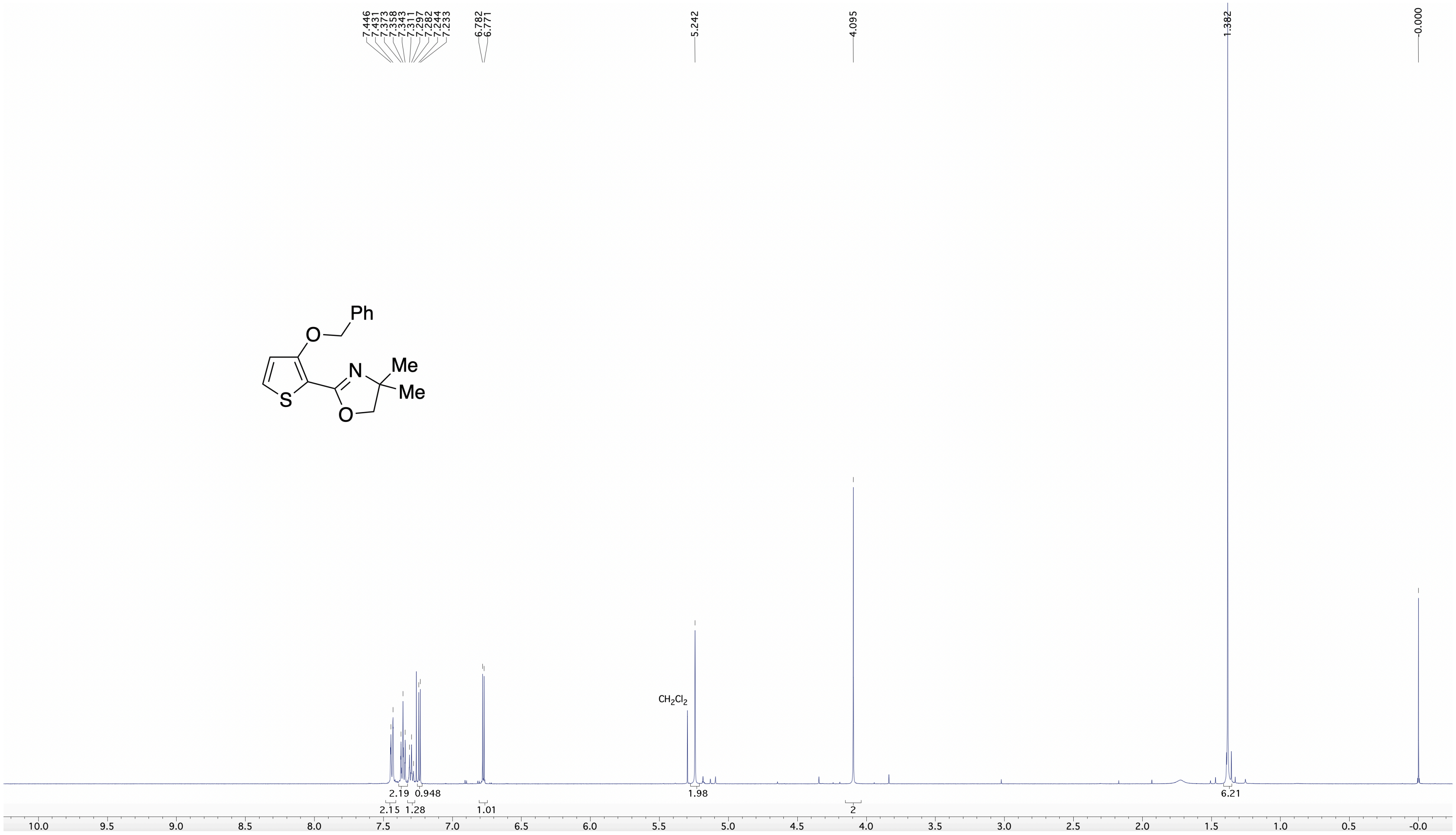

Figure S6. 125 MHz DEPTQ ^{13}C NMR spectrum of **9**

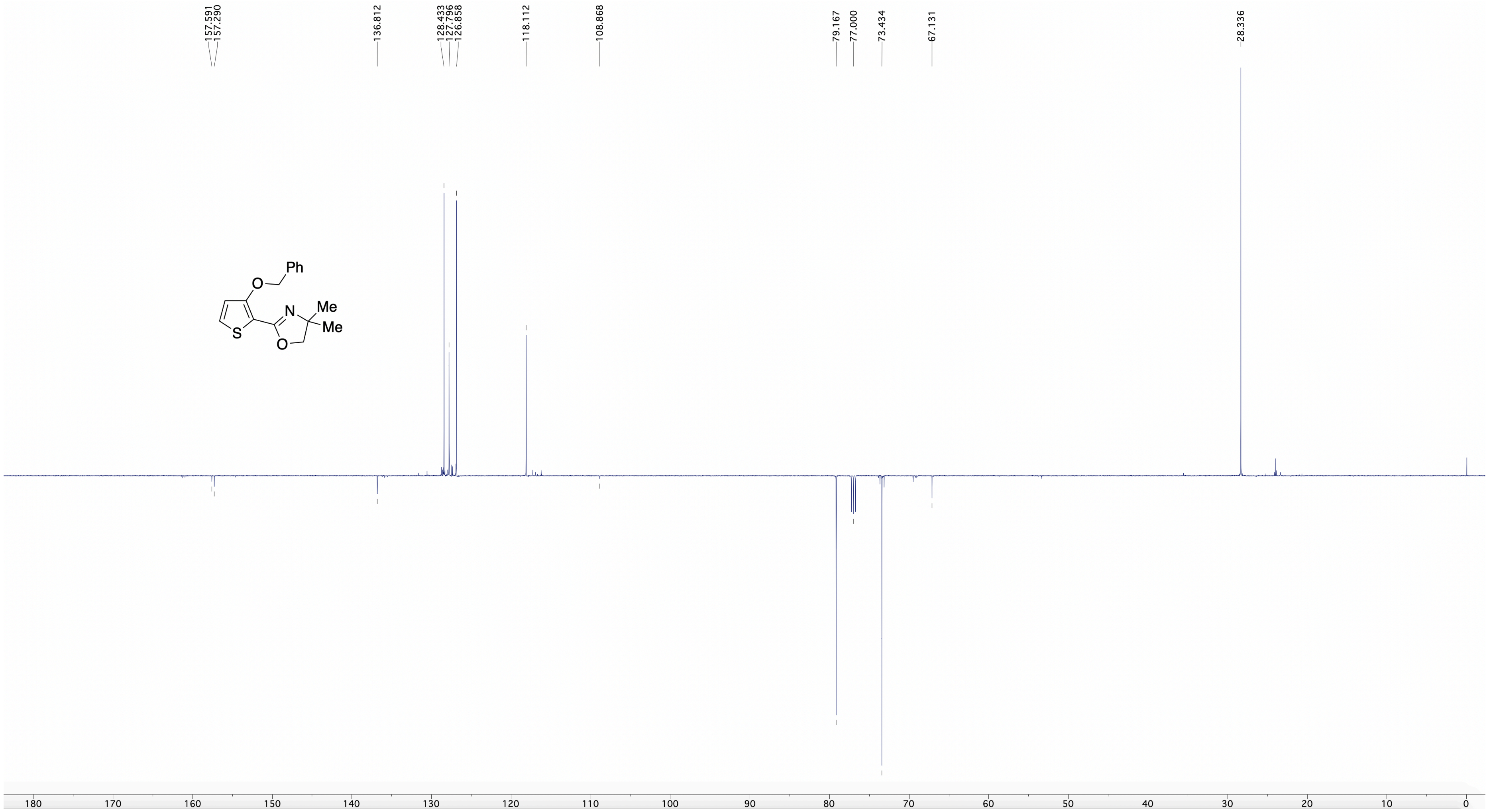

Figure S7. 125 MHz DEPTQ ^{13}C NMR spectrum of **19**

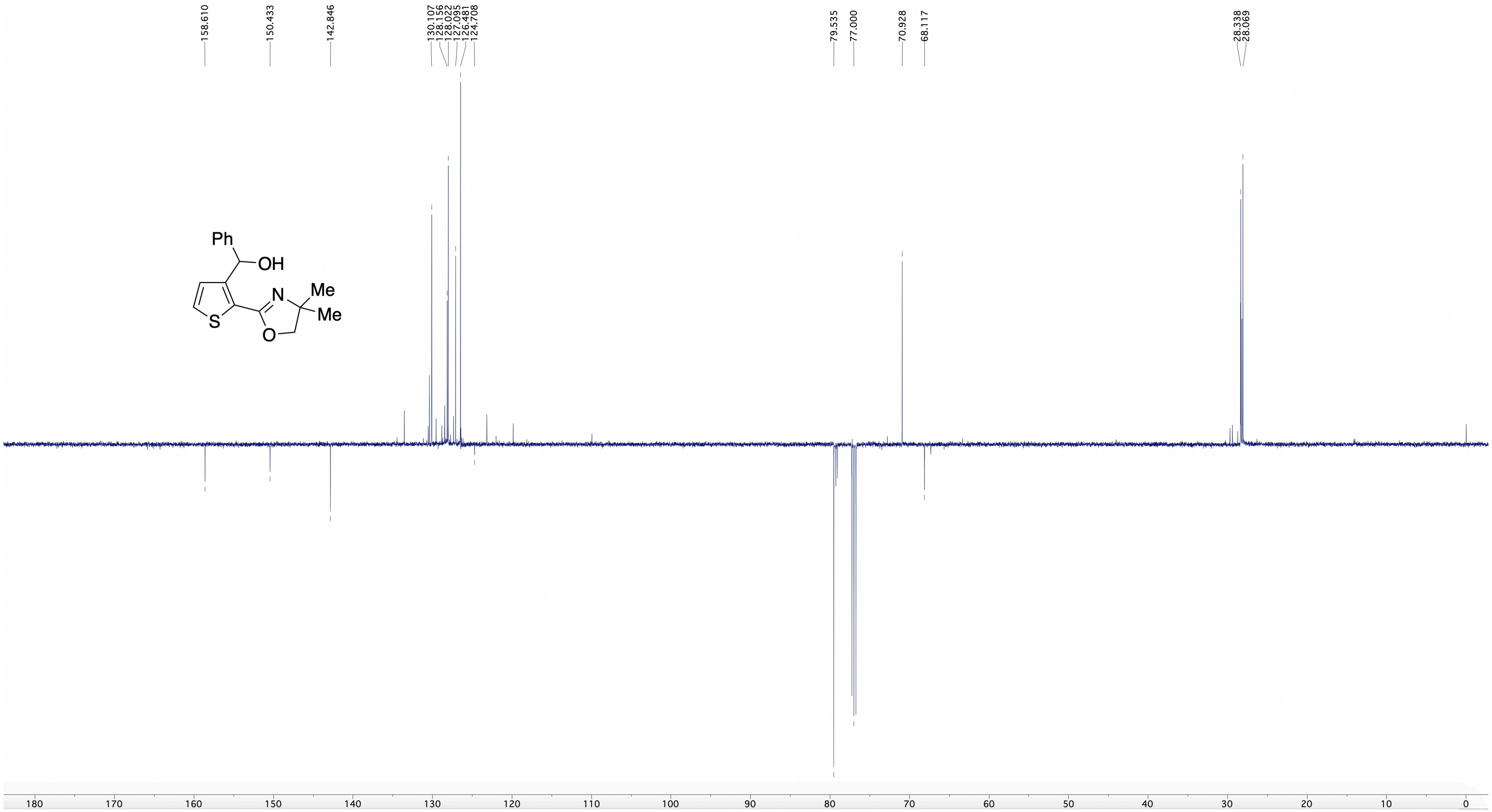

Figure S8. 400 MHz ^{1}H NMR spectrum of **20**

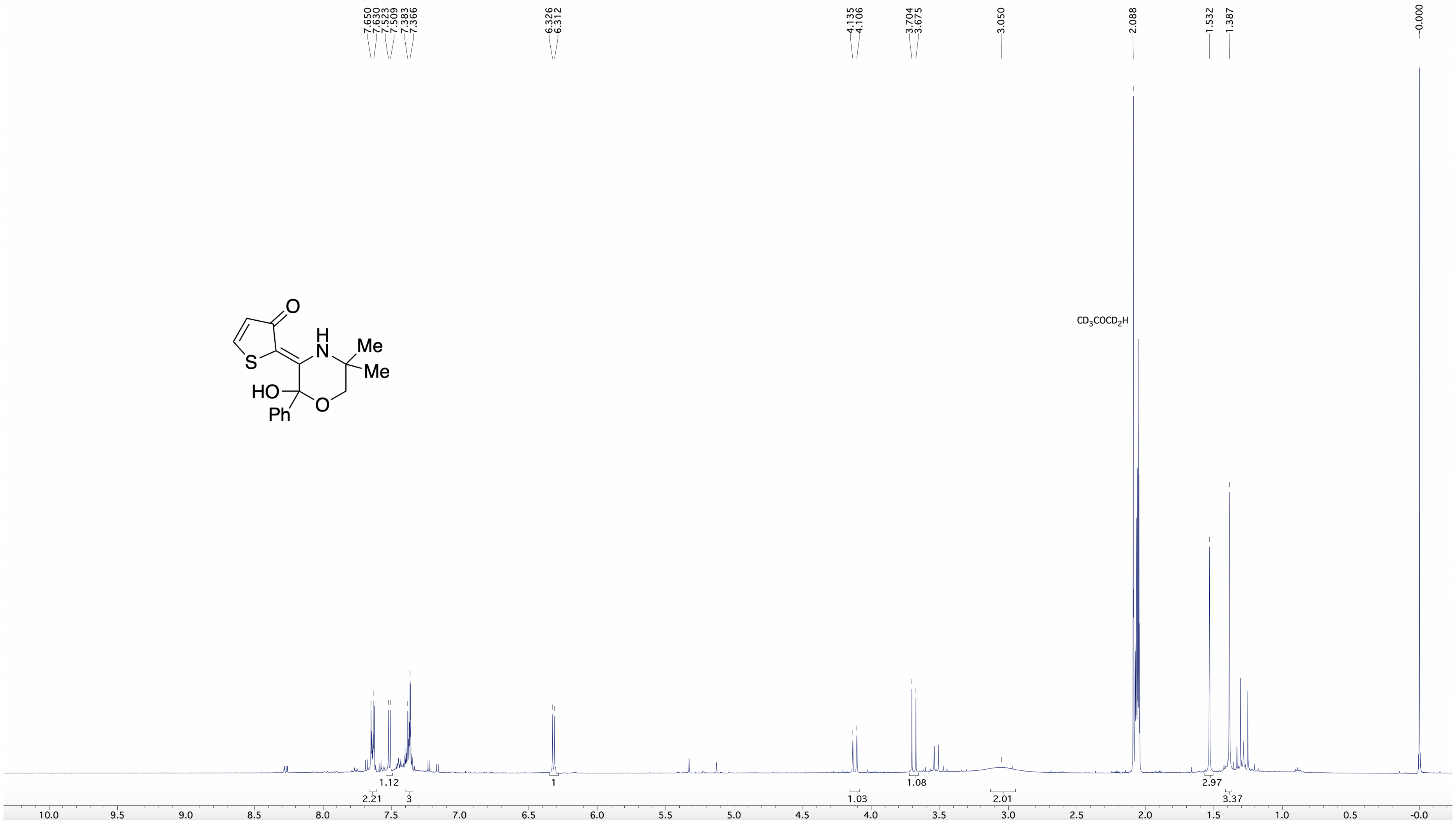

Figure S9. 125 MHz DEPTQ ^{13}C NMR spectrum of **20**

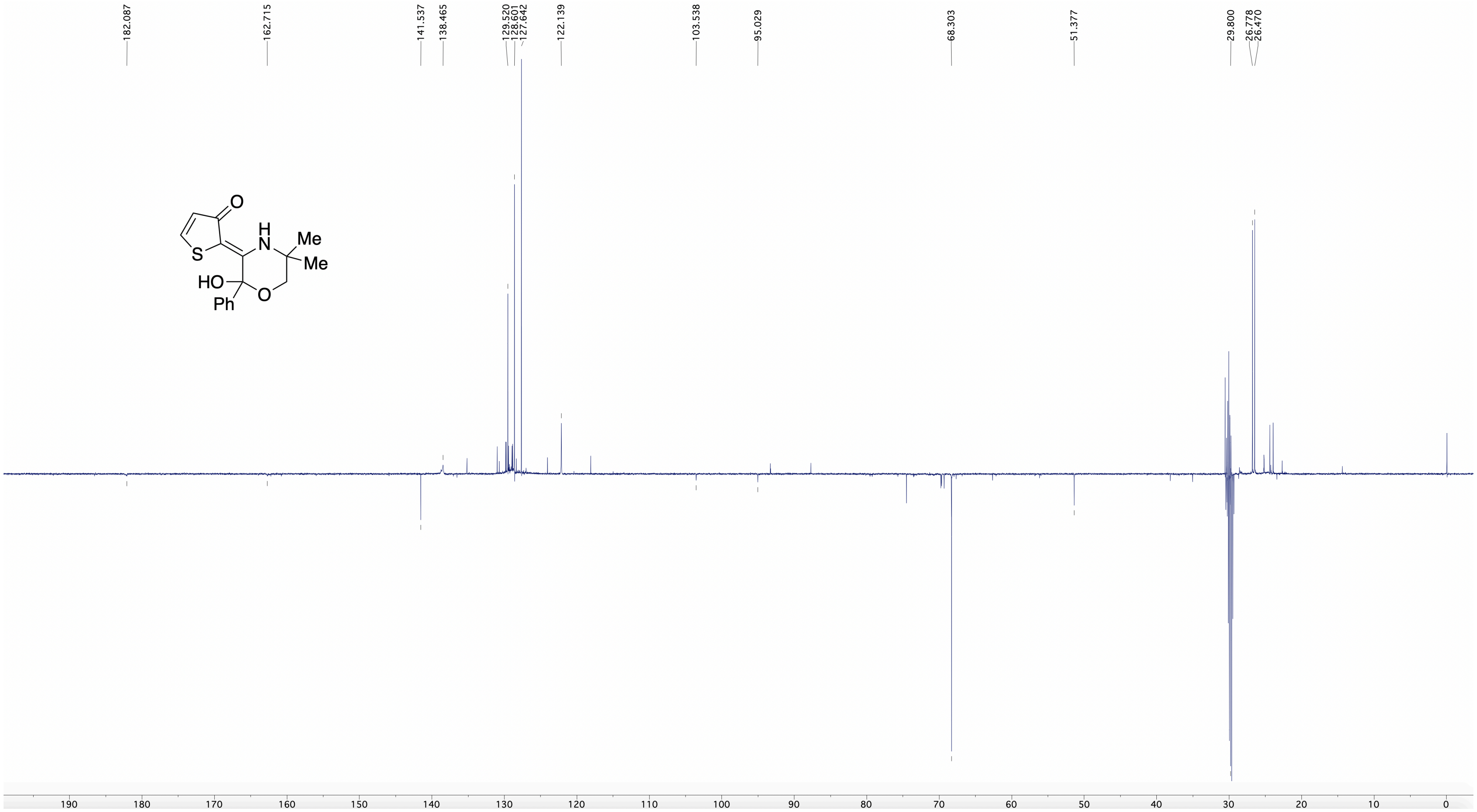

Figure S10. 500 MHz ^{1}H NMR spectrum of **10**

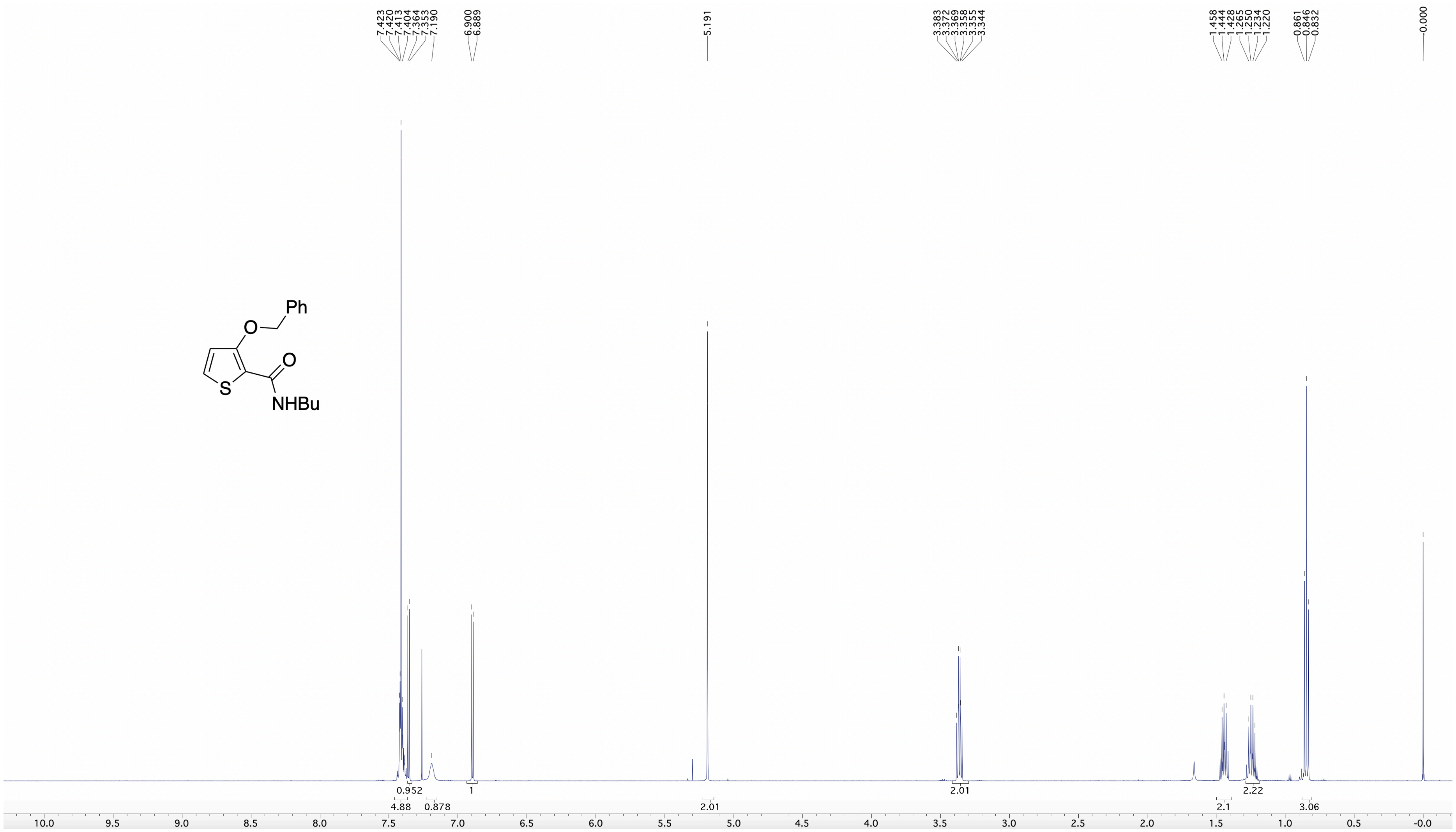

Figure S11. 125 MHz DEPTQ ^{13}C NMR spectrum of **10**

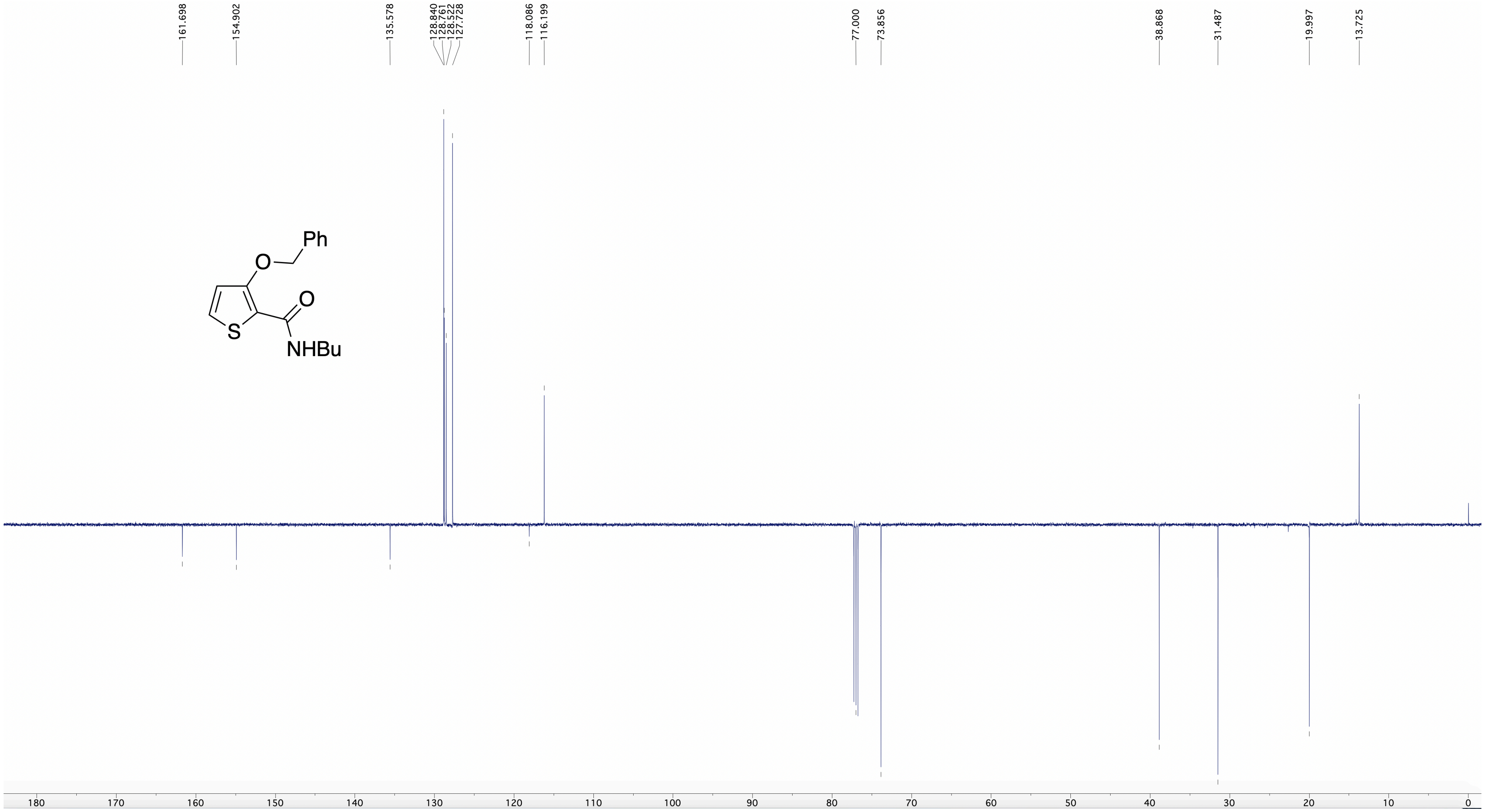

Figure S12. 400 MHz ^1H NMR spectrum of **25**

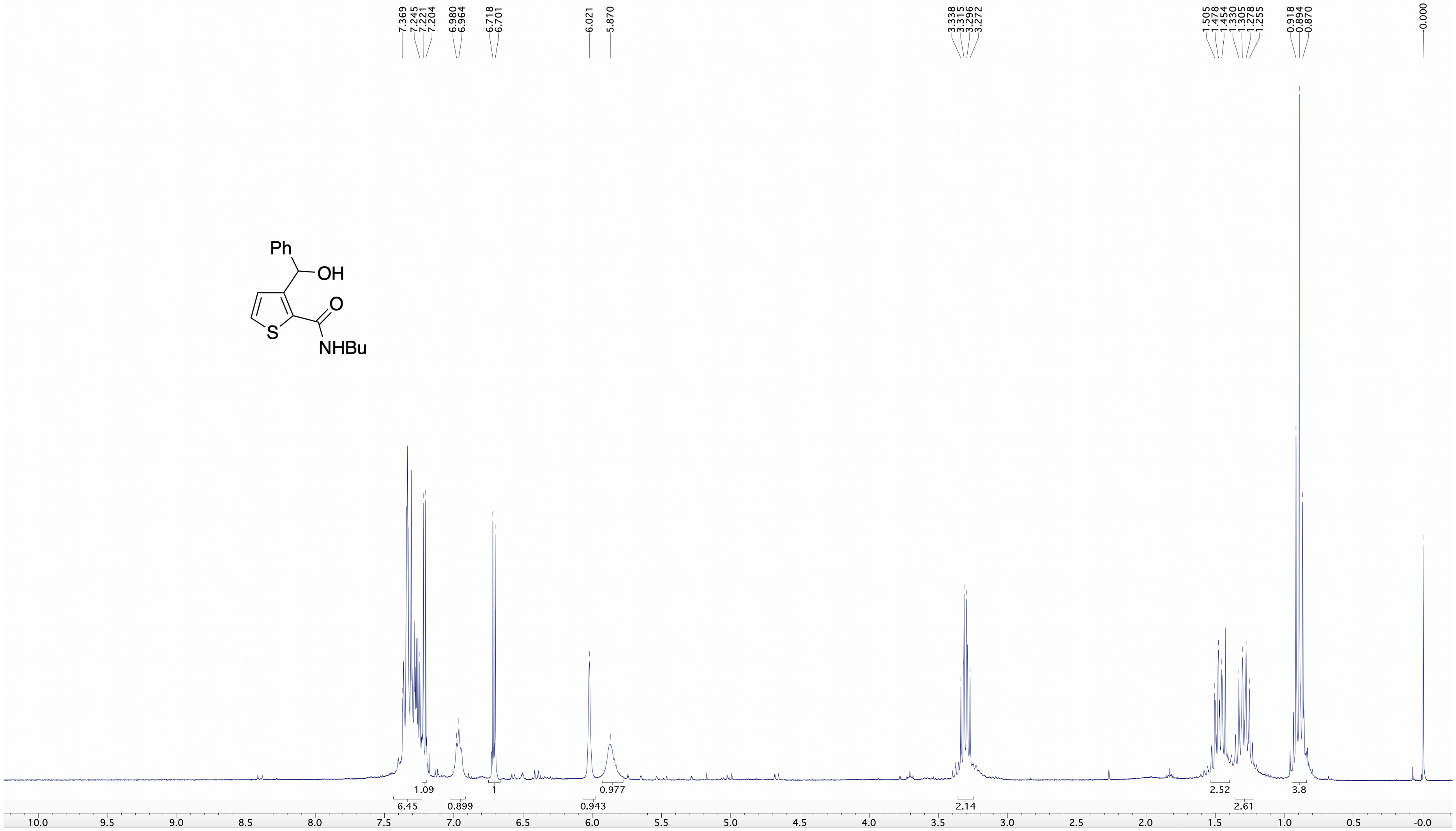

Figure S13. 75 MHz DEPTQ ^{13}C NMR spectrum of **25**

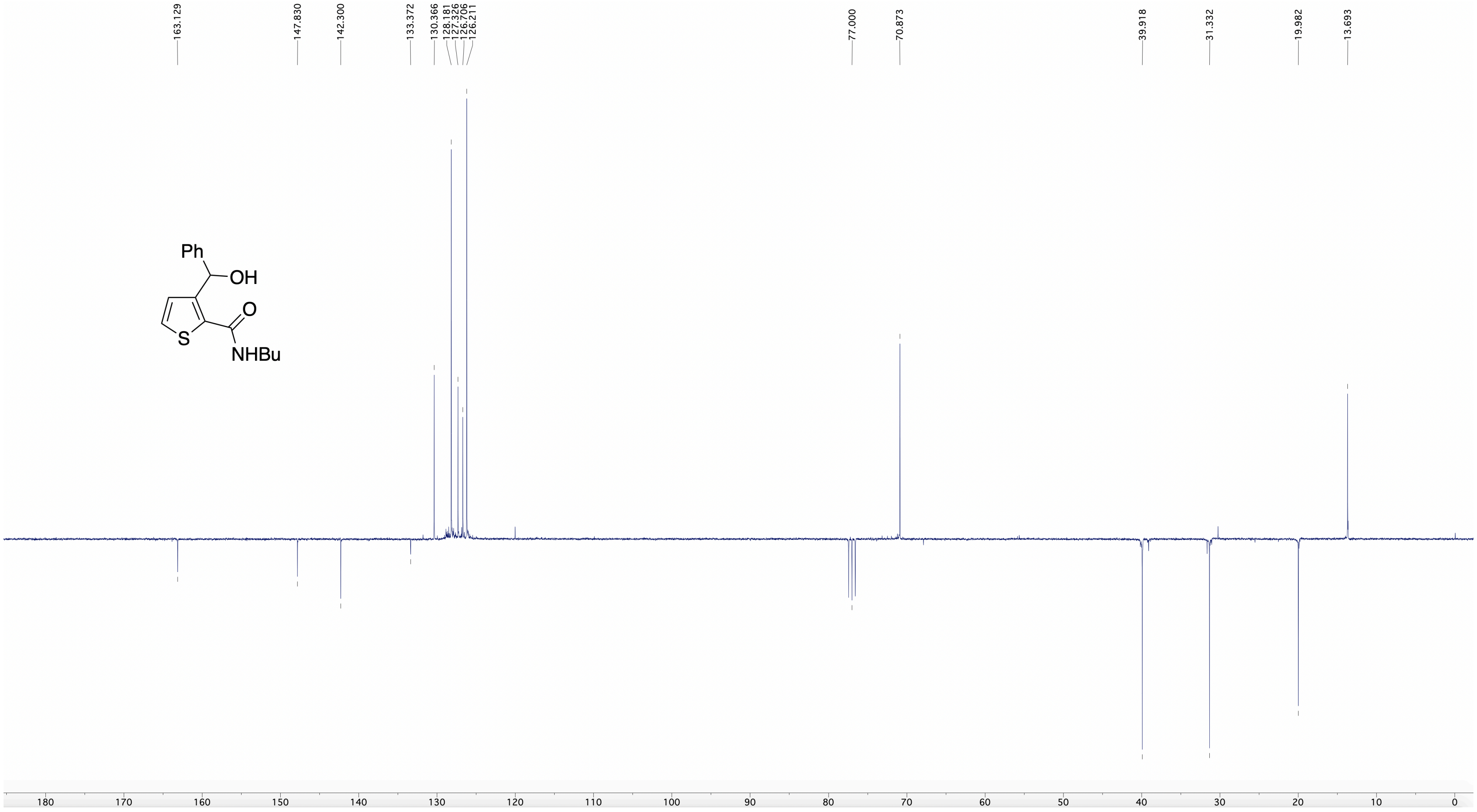

Figure S14. 400 MHz ^1H NMR spectrum of **26**

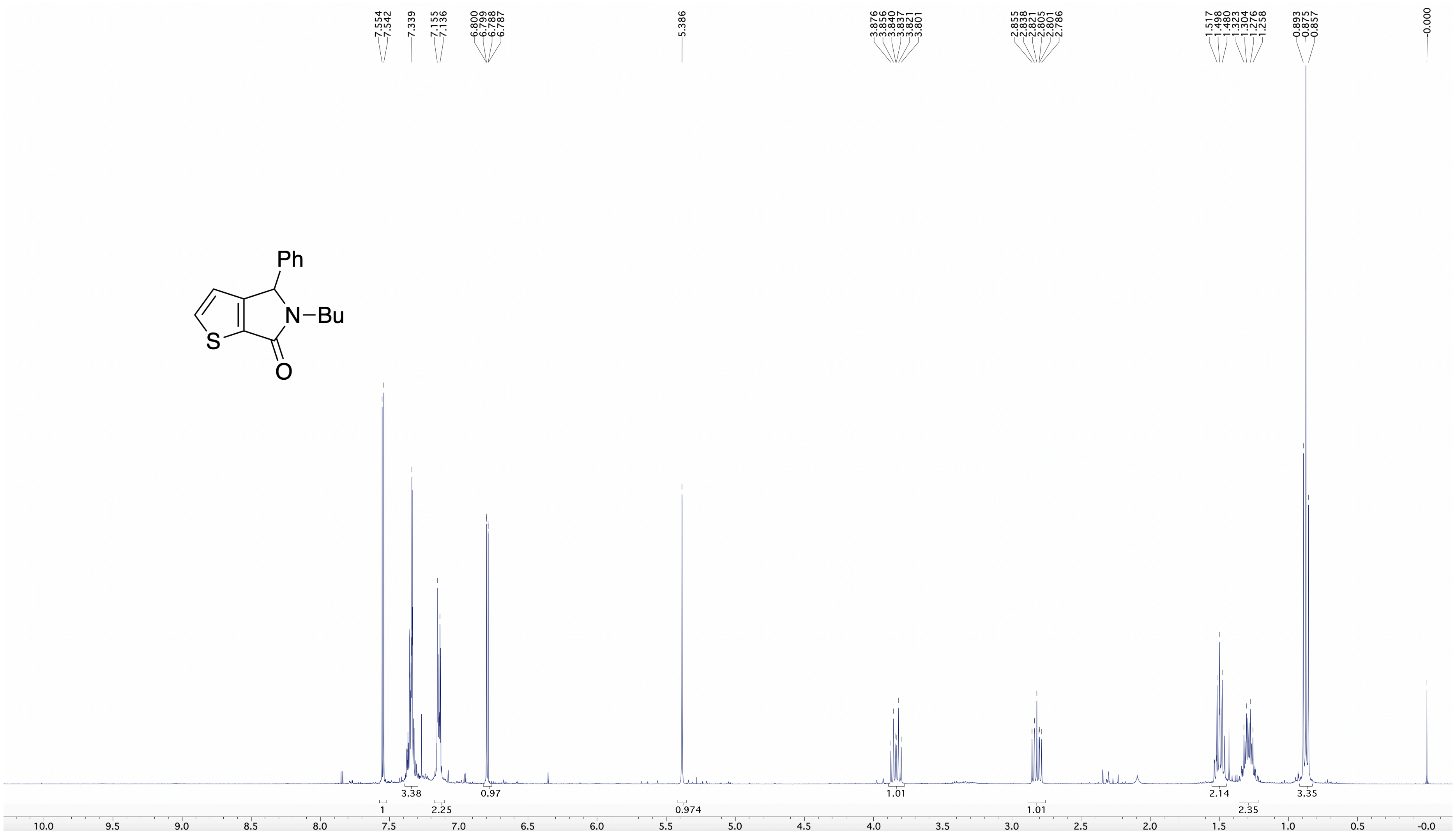

Figure S15. 125 MHz DEPTQ ^{13}C NMR spectrum of **26**

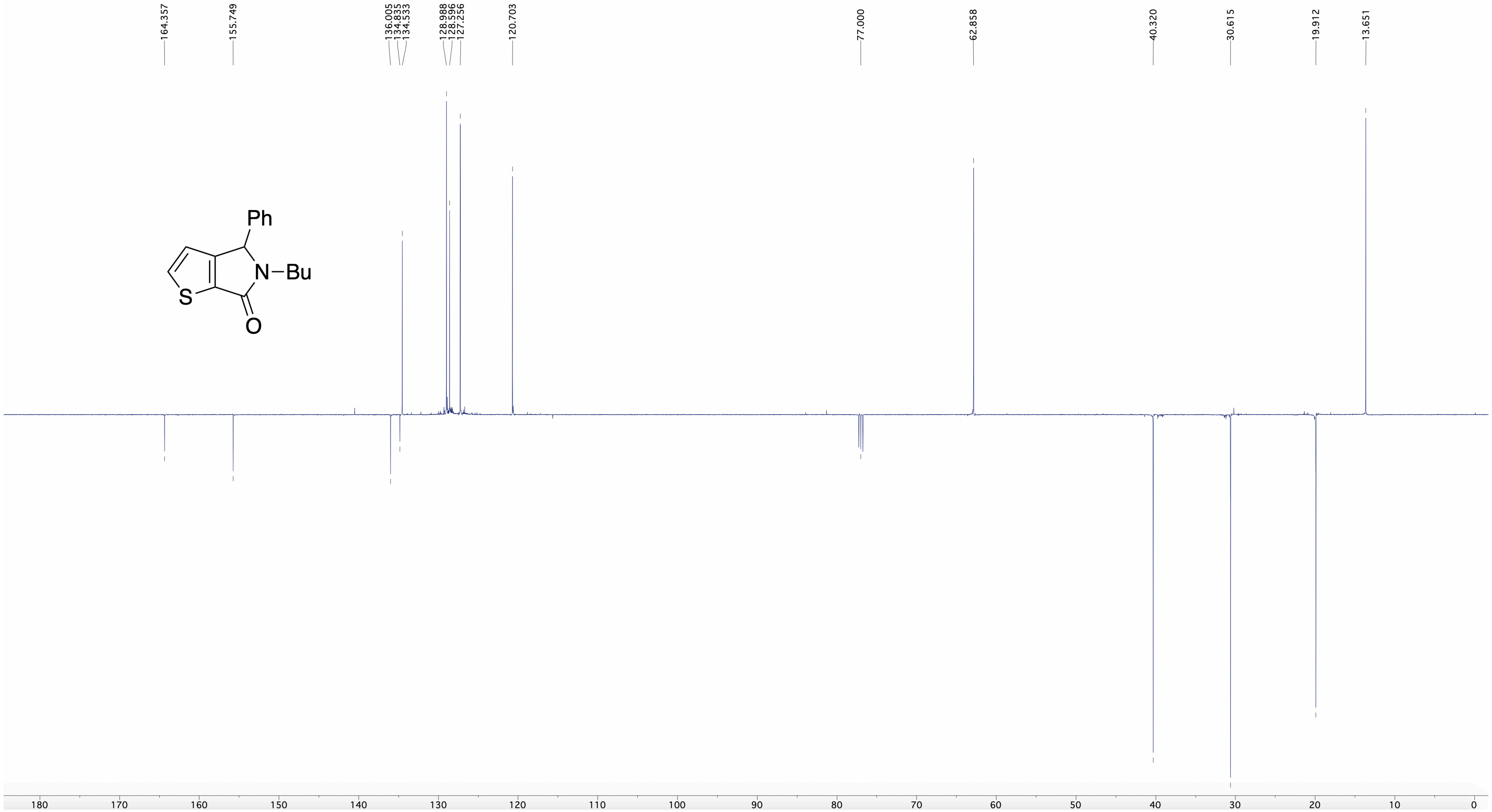

Figure S16. 500 MHz ^{1}H NMR spectrum of **30**

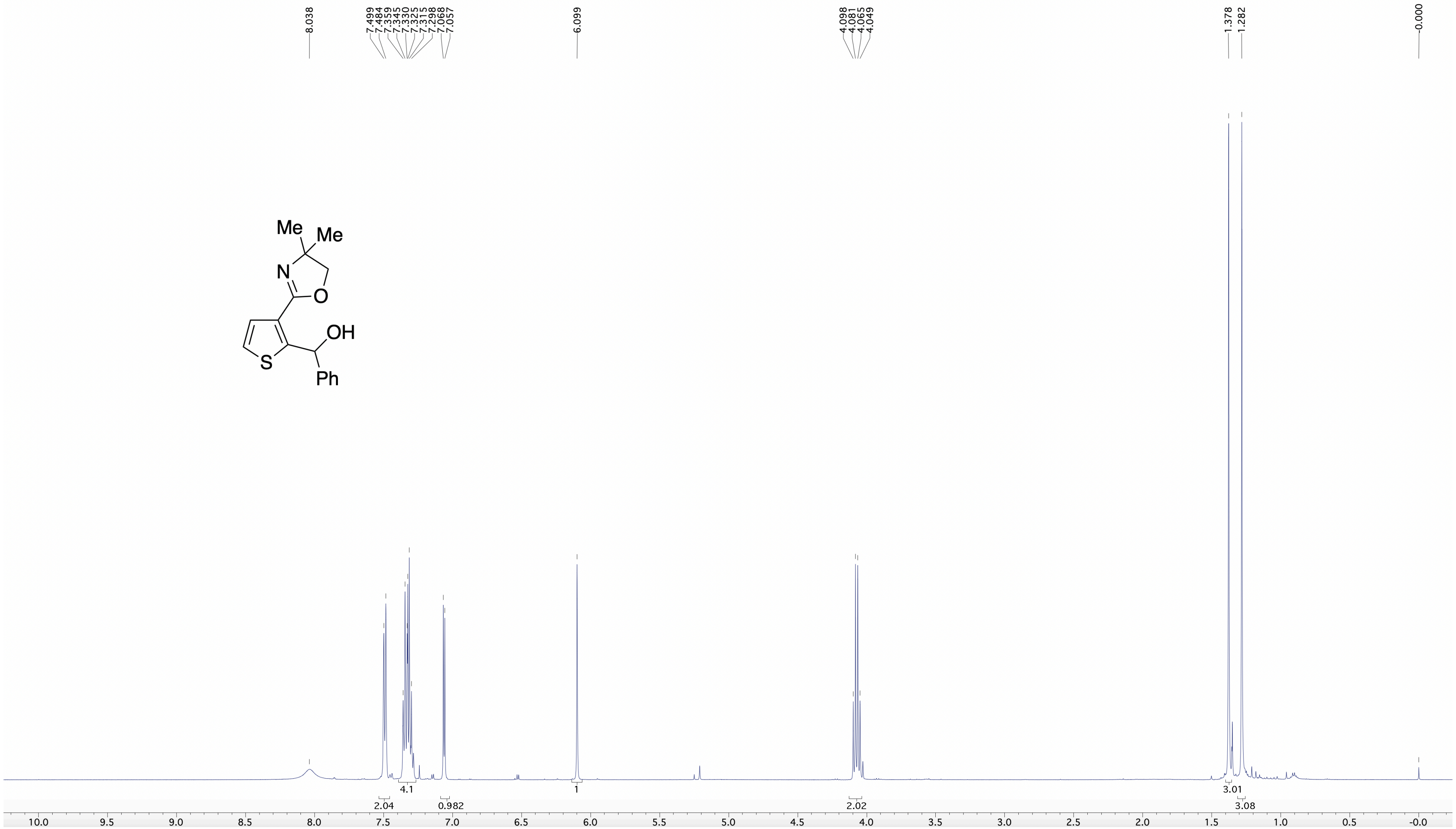

Figure S17. 125 MHz DEPTQ ^{13}C NMR spectrum of **30**

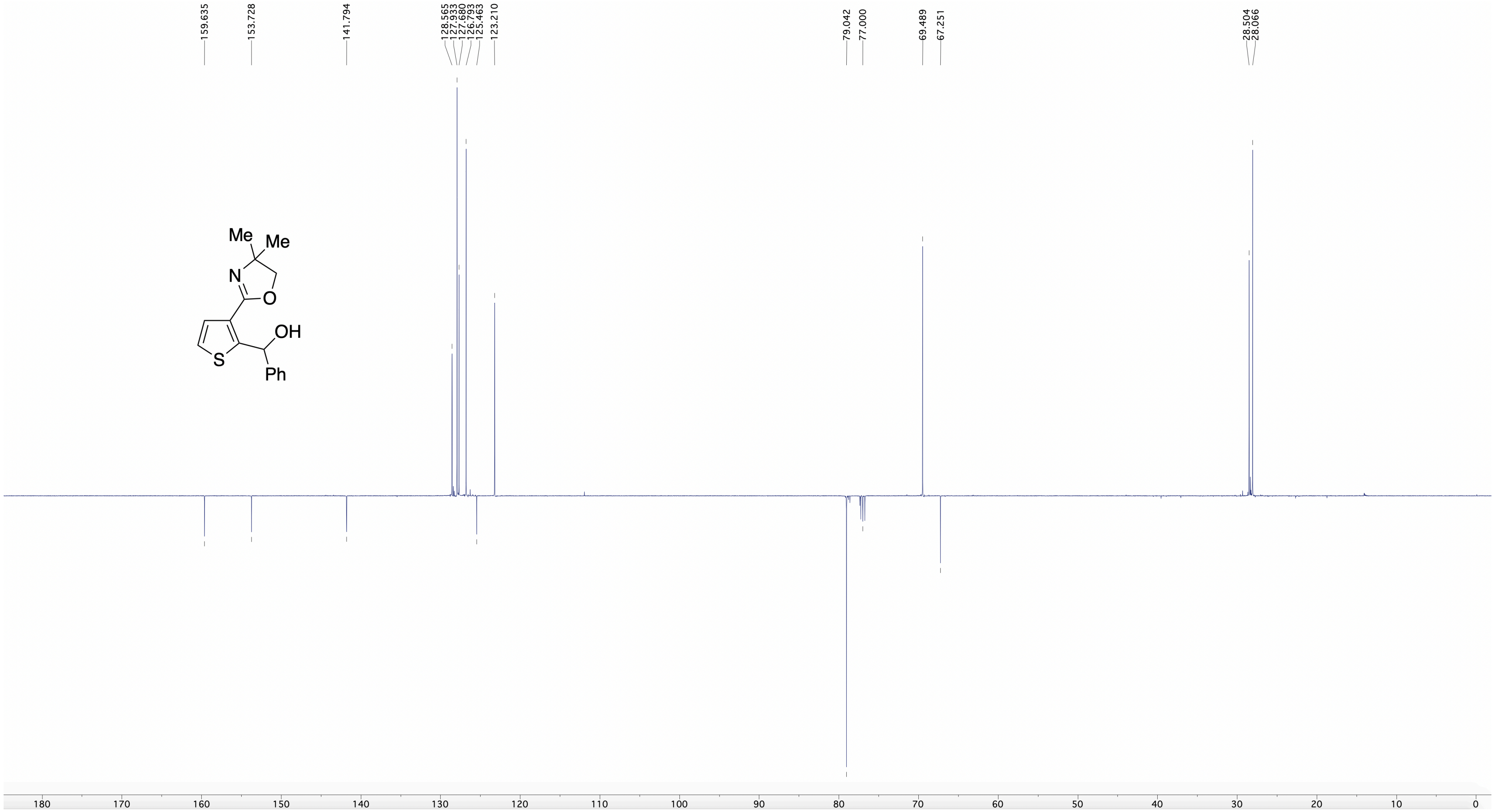

Figure S18. 500 MHz ¹H NMR spectrum of **33**

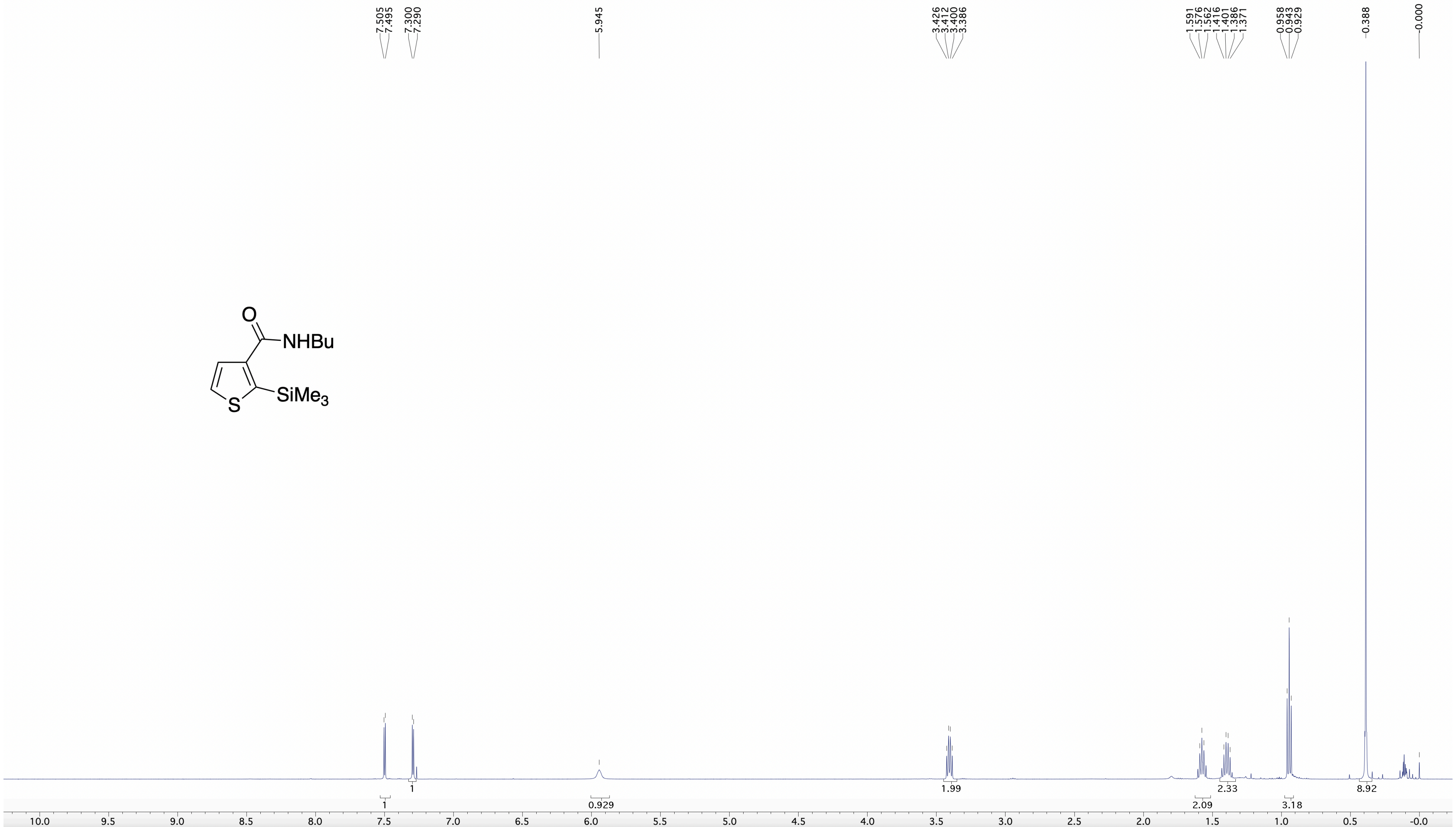

Figure S19. 125 MHz DEPTQ ^{13}C NMR spectrum of **33**

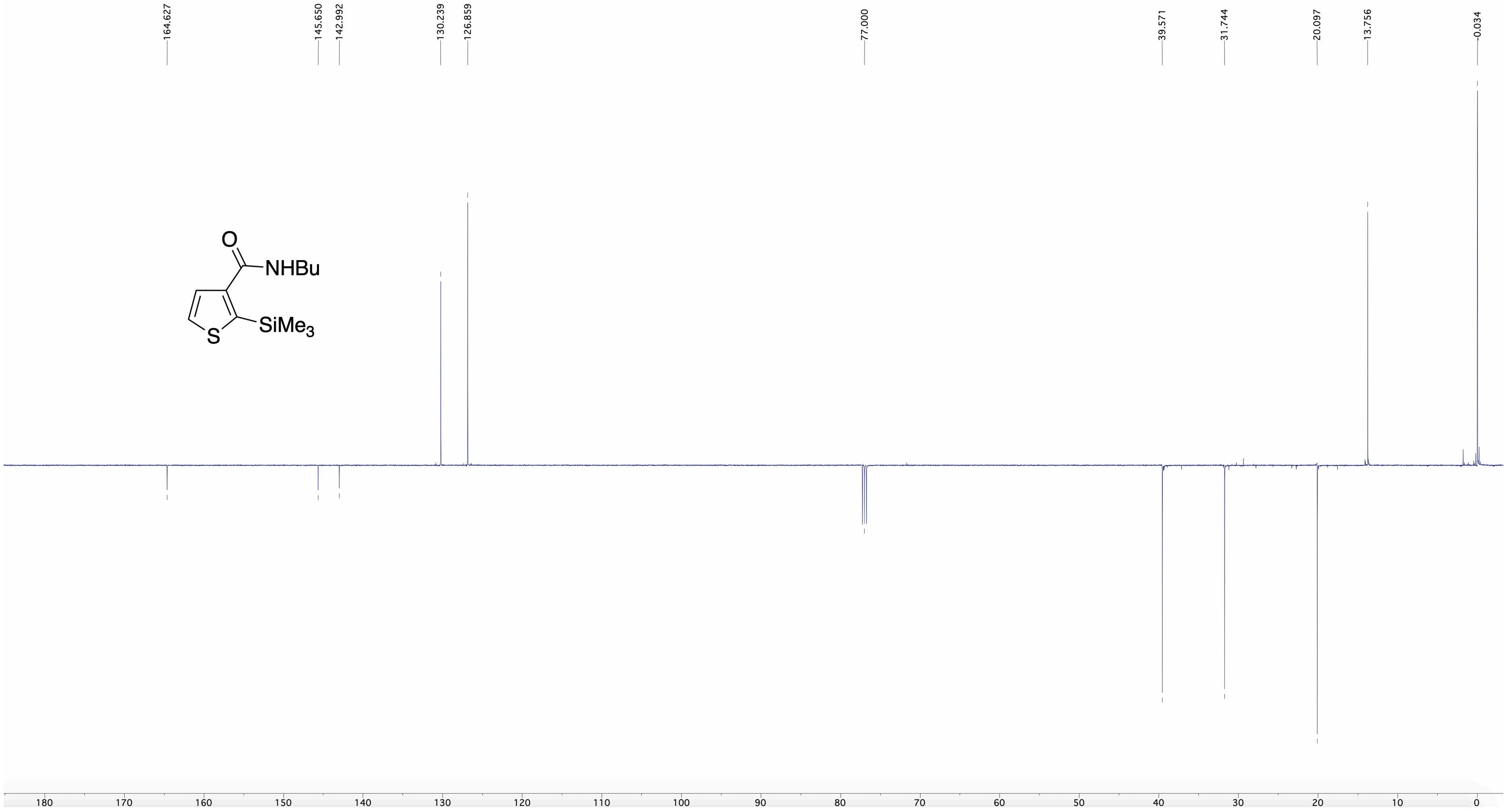

Figure S20. 400 MHz ^{1}H NMR spectrum of **35**

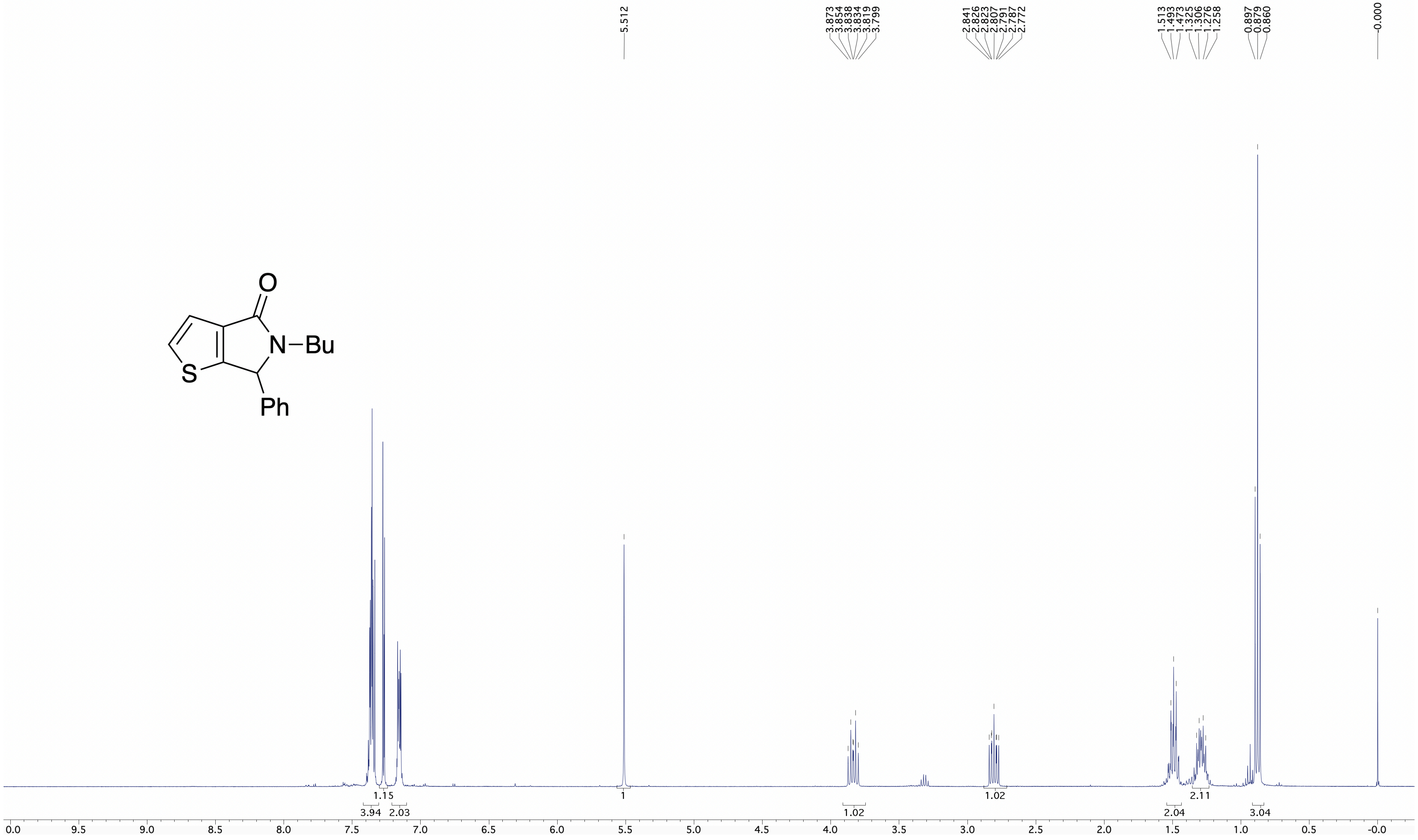

Figure S21. 125 MHz DEPTQ ^{13}C NMR spectrum of **35**

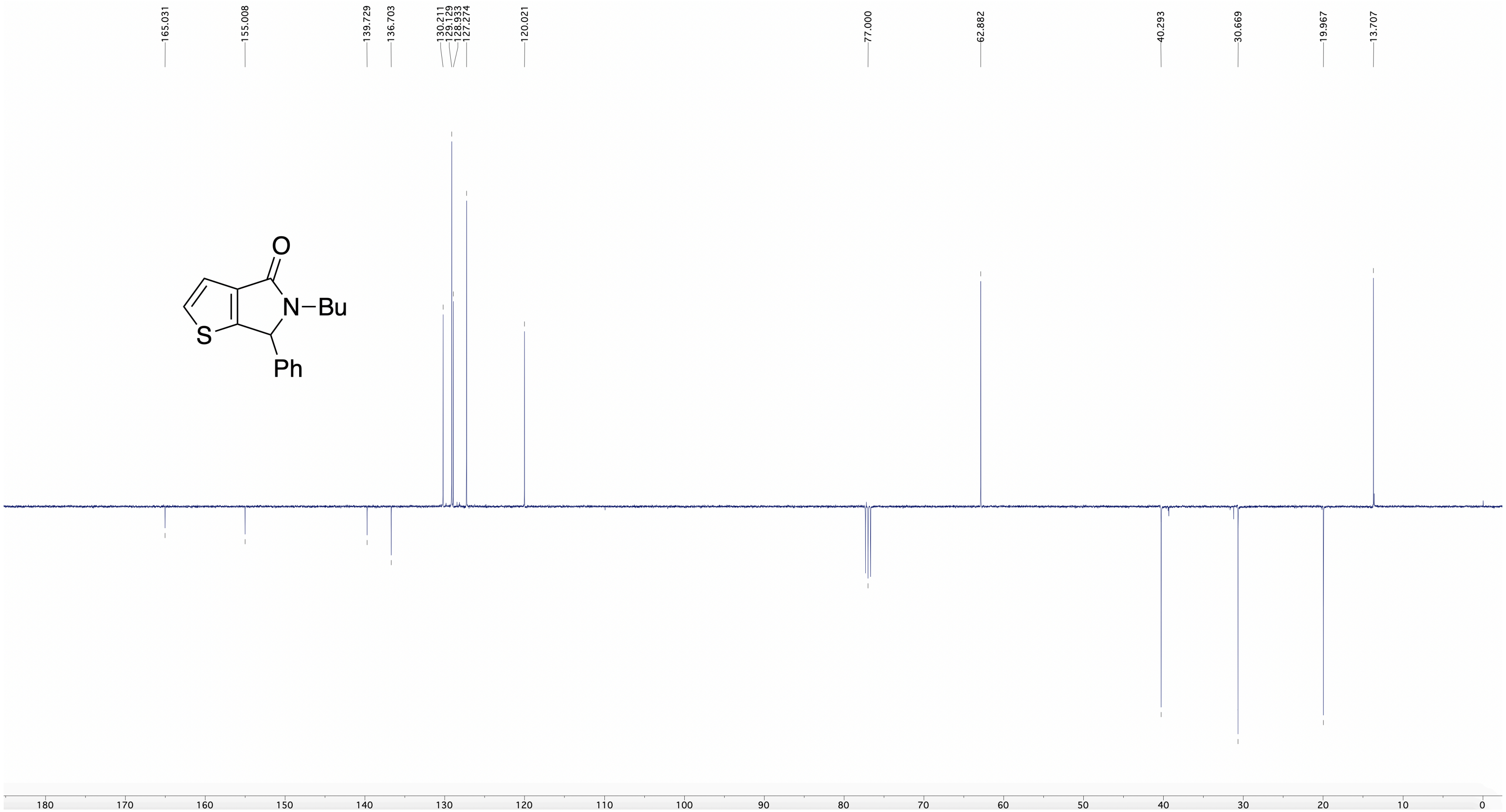

Figure S22. 400 MHz ^{1}H NMR spectrum of **36**

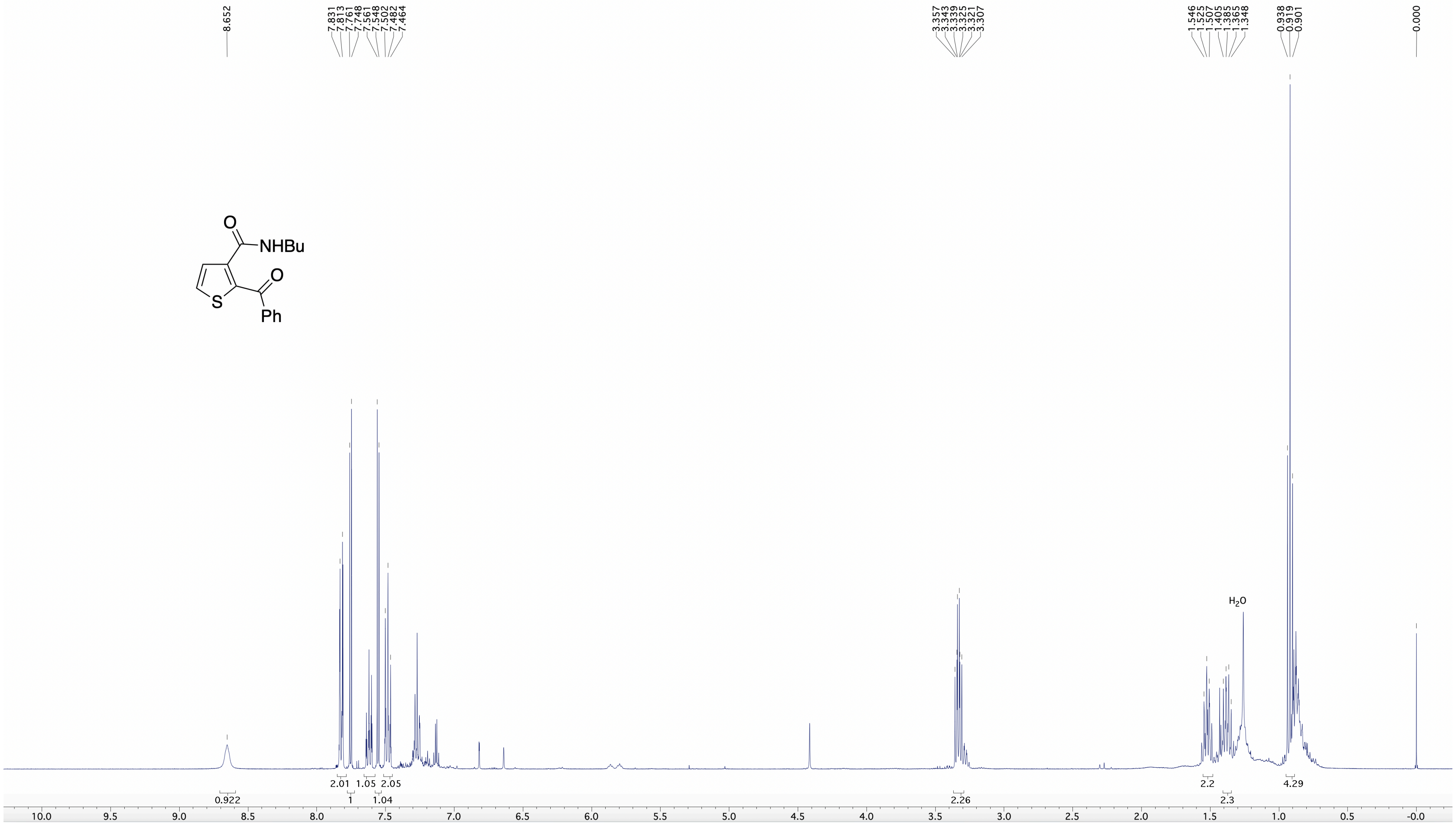

Figure S23. 100 MHz DEPTQ ^{13}C NMR spectrum of **36**

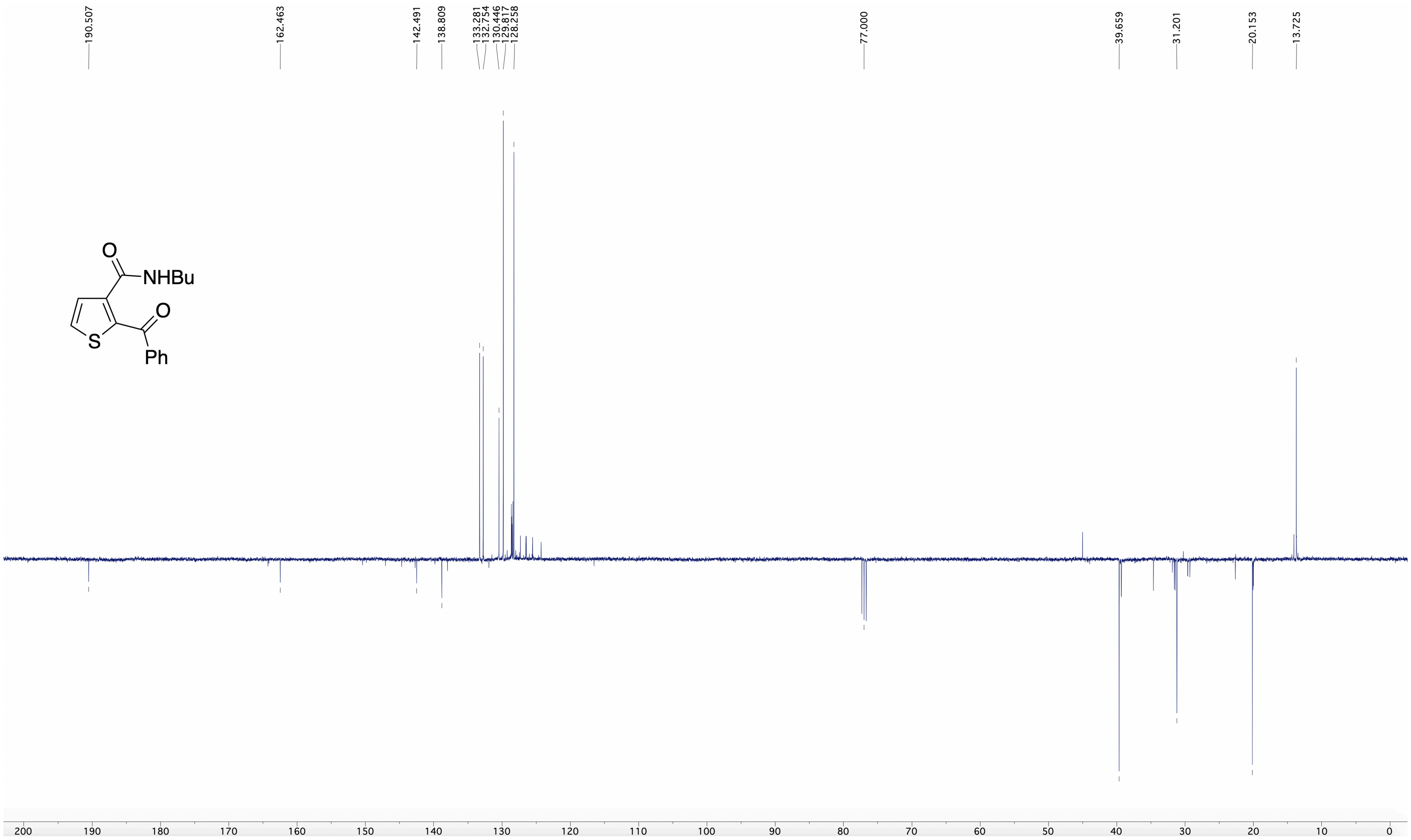

Figure S24. 100 MHz DEPTQ ^{13}C NMR spectrum of **37**

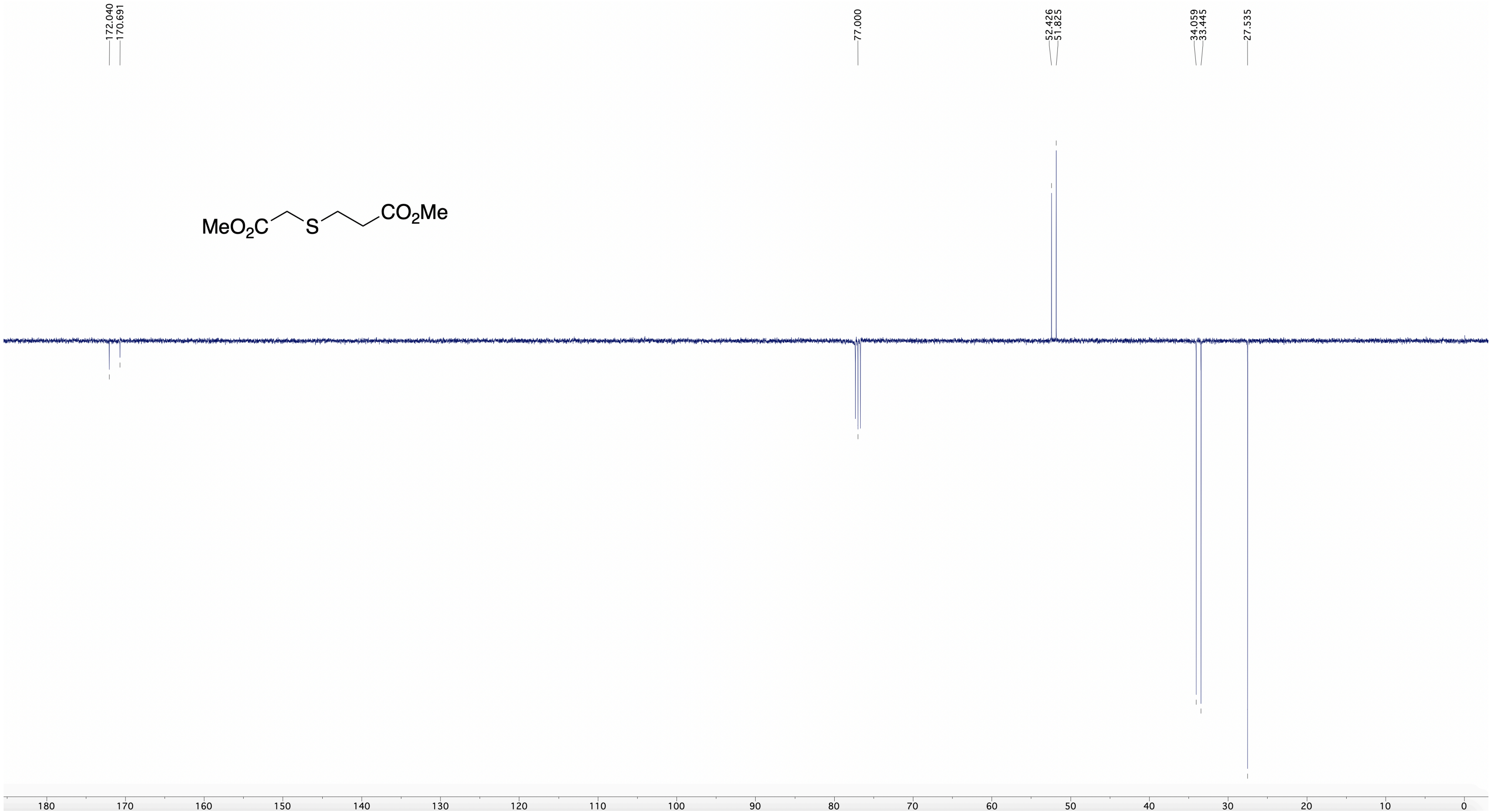

Figure S25. 125 MHz DEPTQ ^{13}C NMR spectrum of **39**

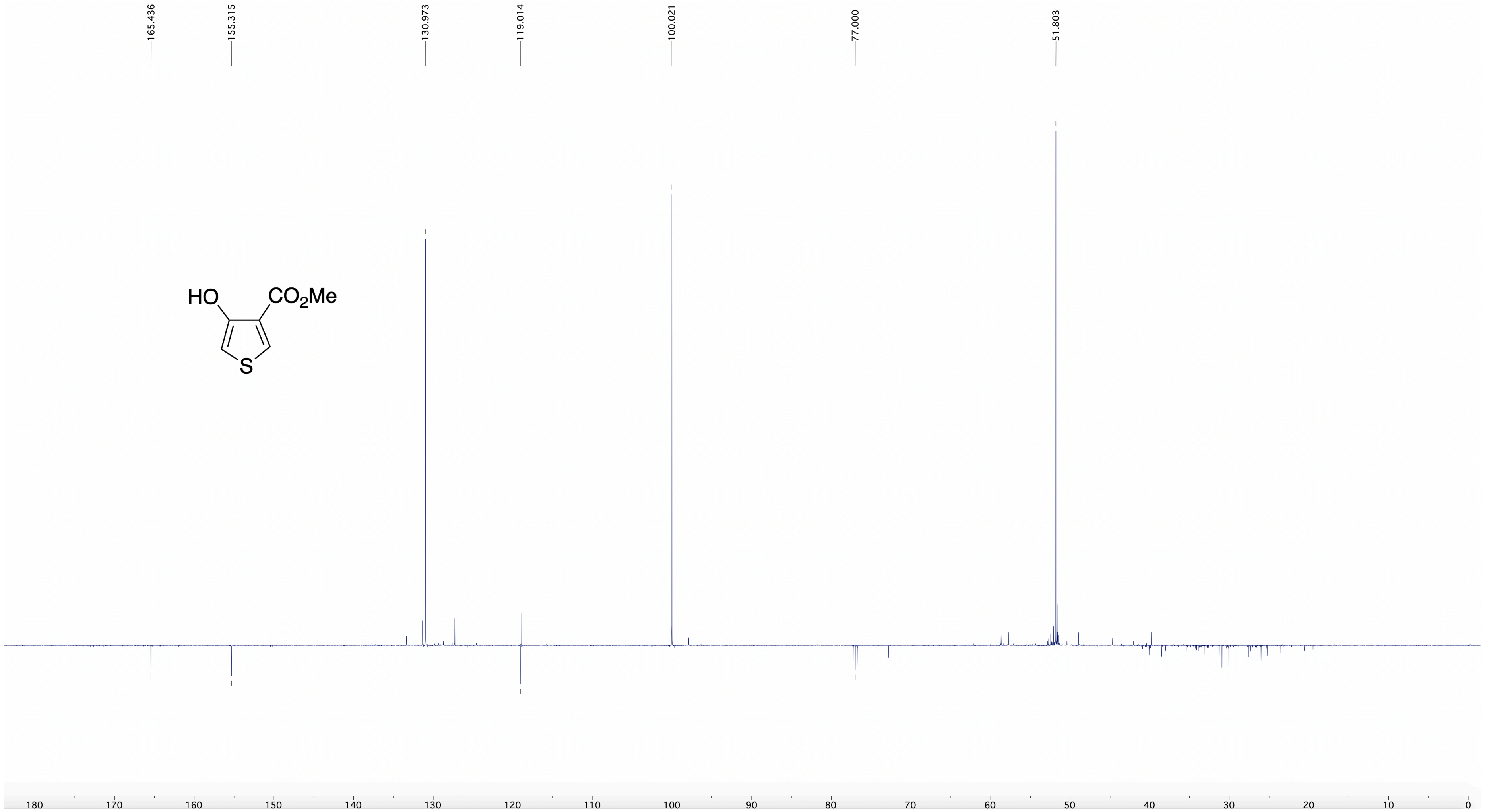

Figure S26. 500 MHz ^{1}H NMR spectrum of **41**

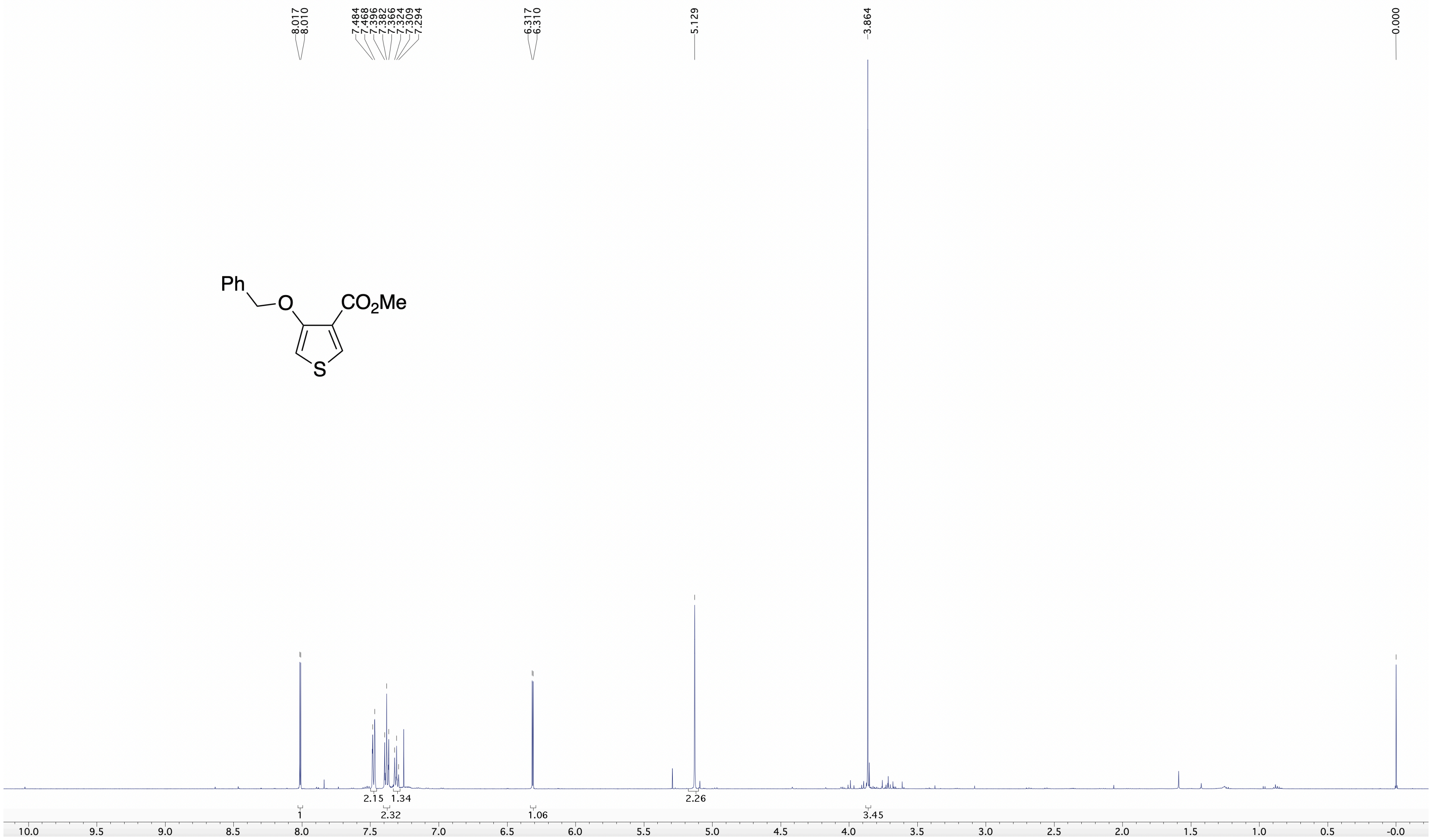

Figure S27. 125 MHz DEPTQ ^{13}C NMR spectrum of **41**

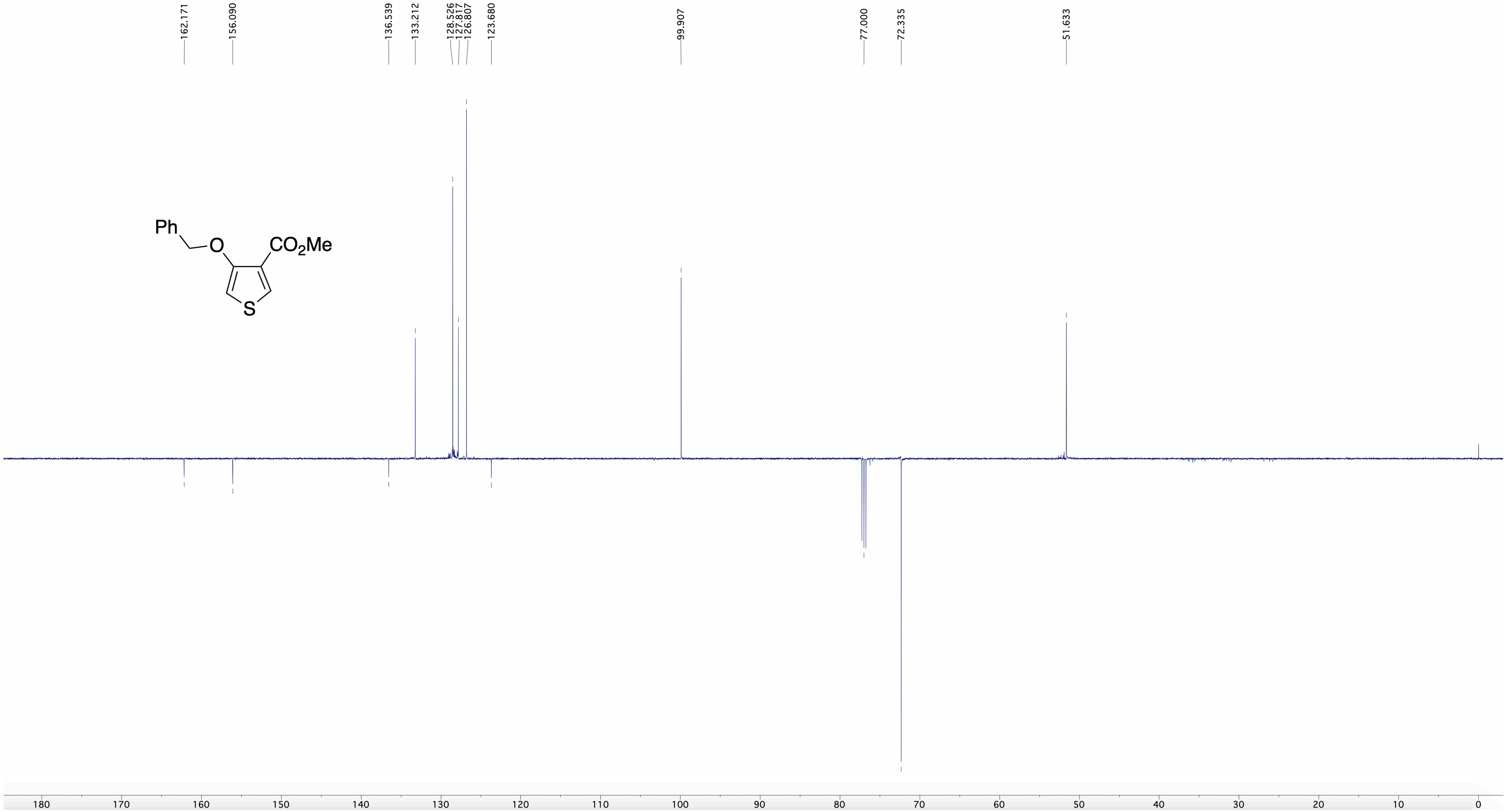

Figure S28. 500 MHz ^{1}H NMR spectrum of **42**

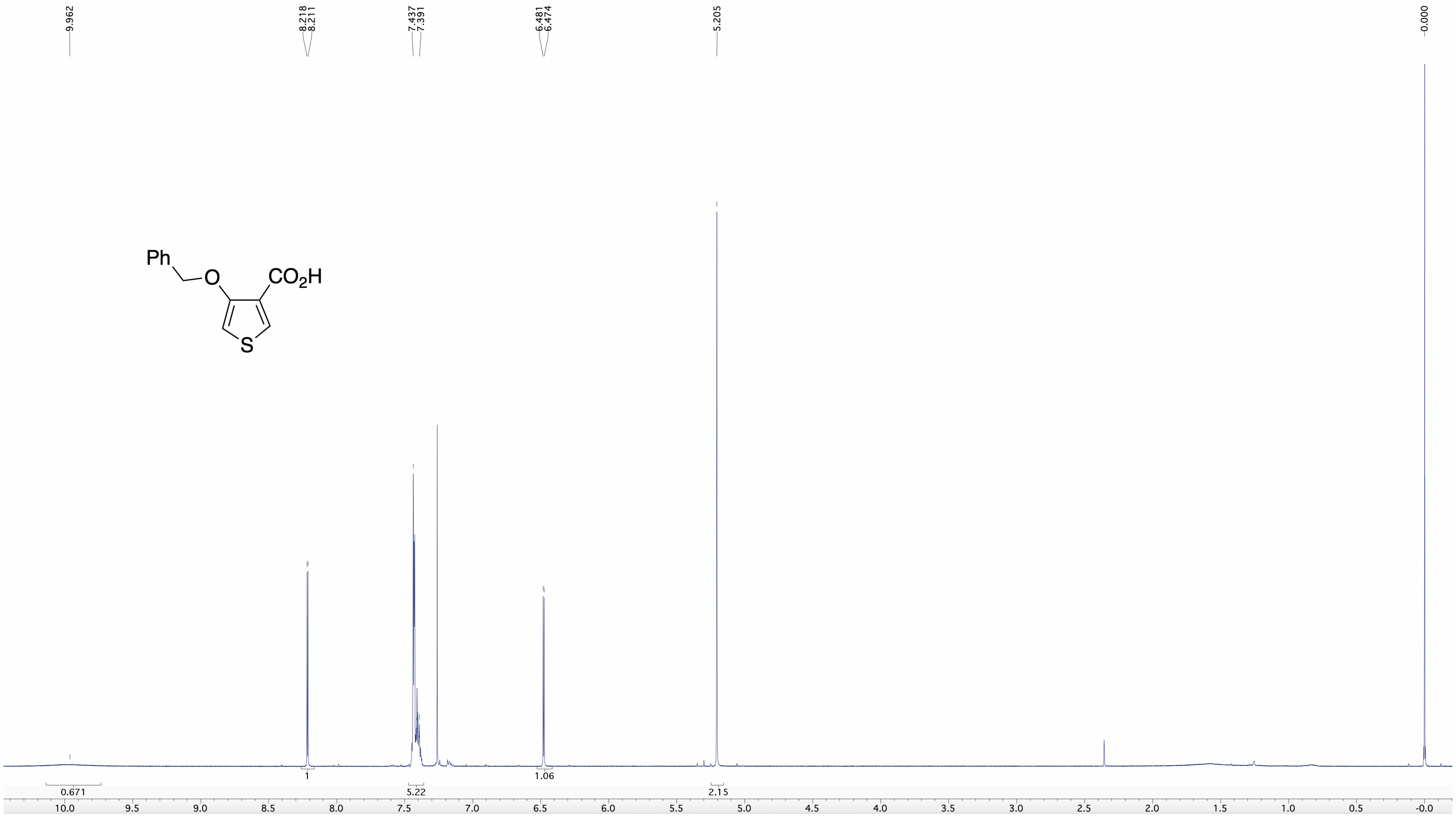

Figure S29. 125 MHz DEPTQ ^{13}C NMR spectrum of **42**

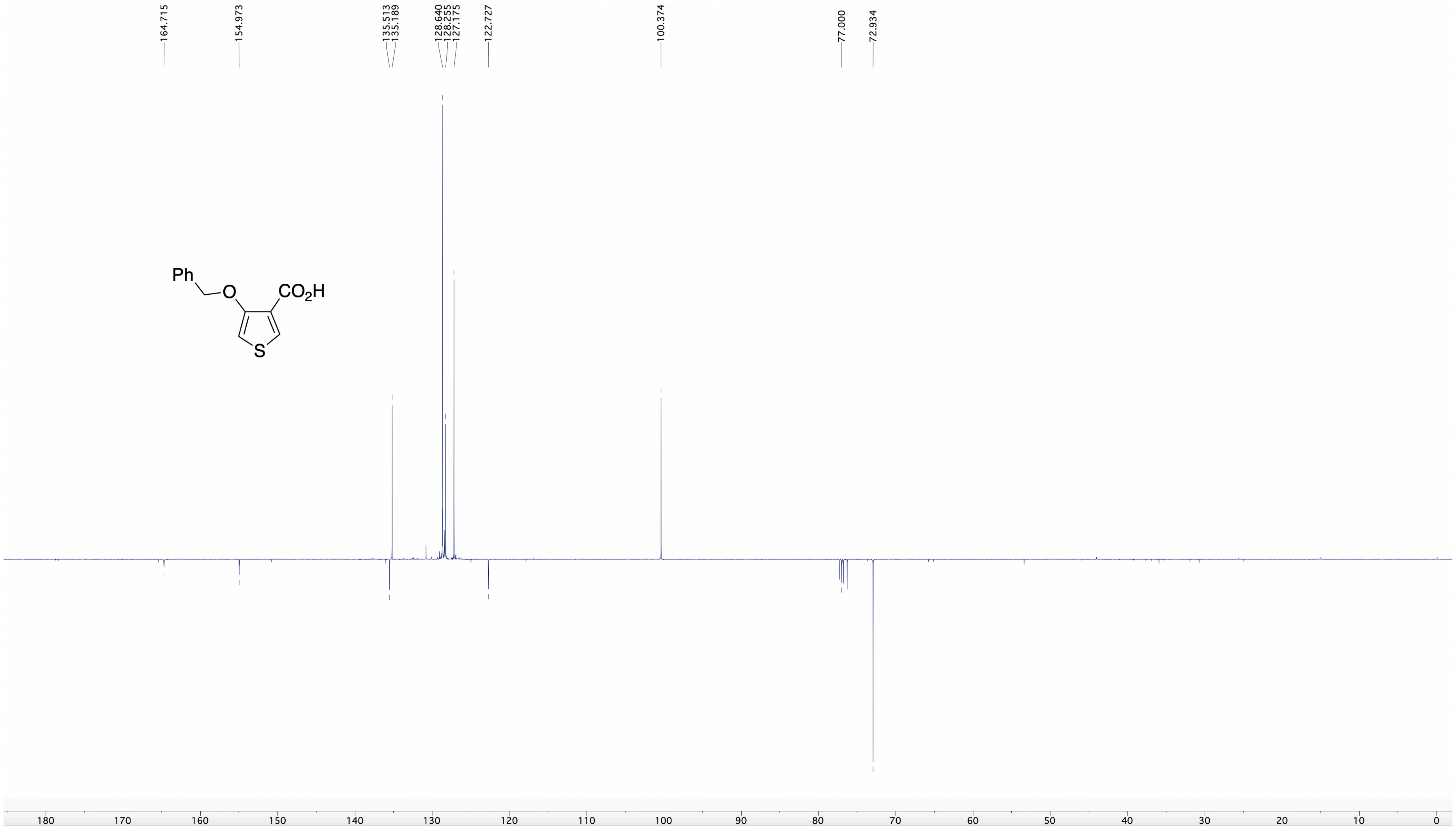

Figure S30. 400 MHz ^{1}H NMR spectrum of **43**

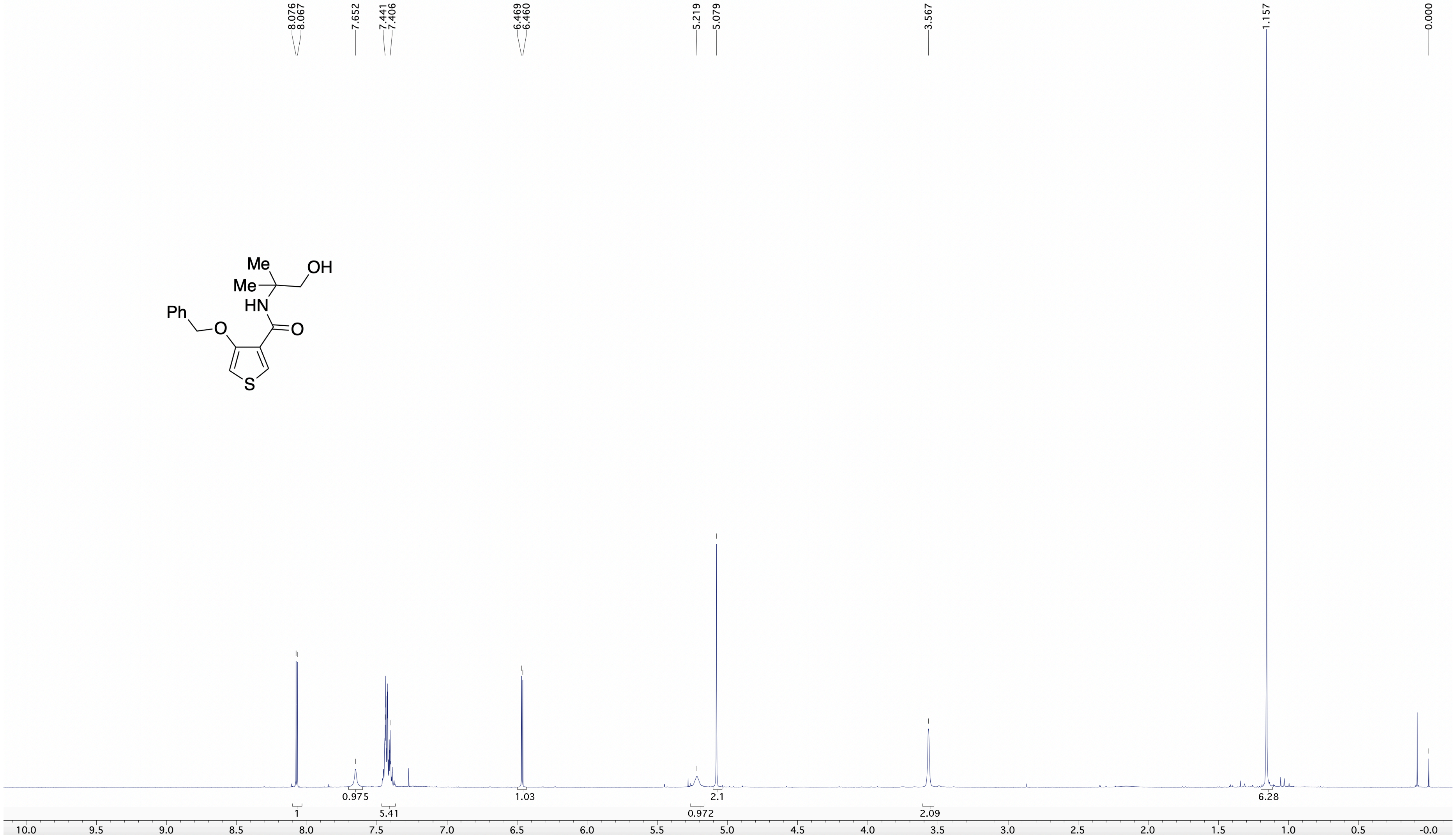

Figure S31. 100 MHz DEPTQ ^{13}C NMR spectrum of **43**

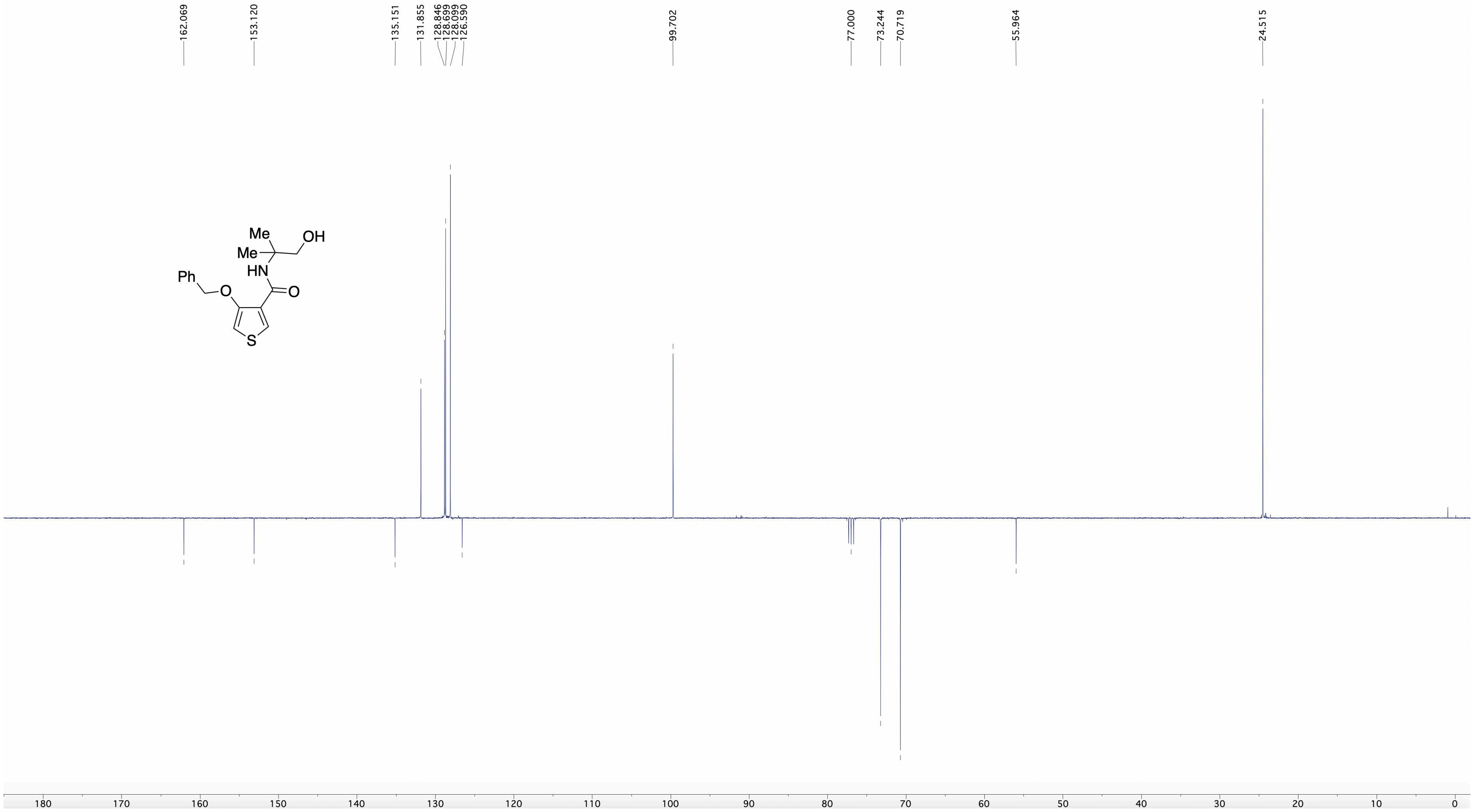

Figure S32. 400 MHz ^{1}H NMR spectrum of **13**

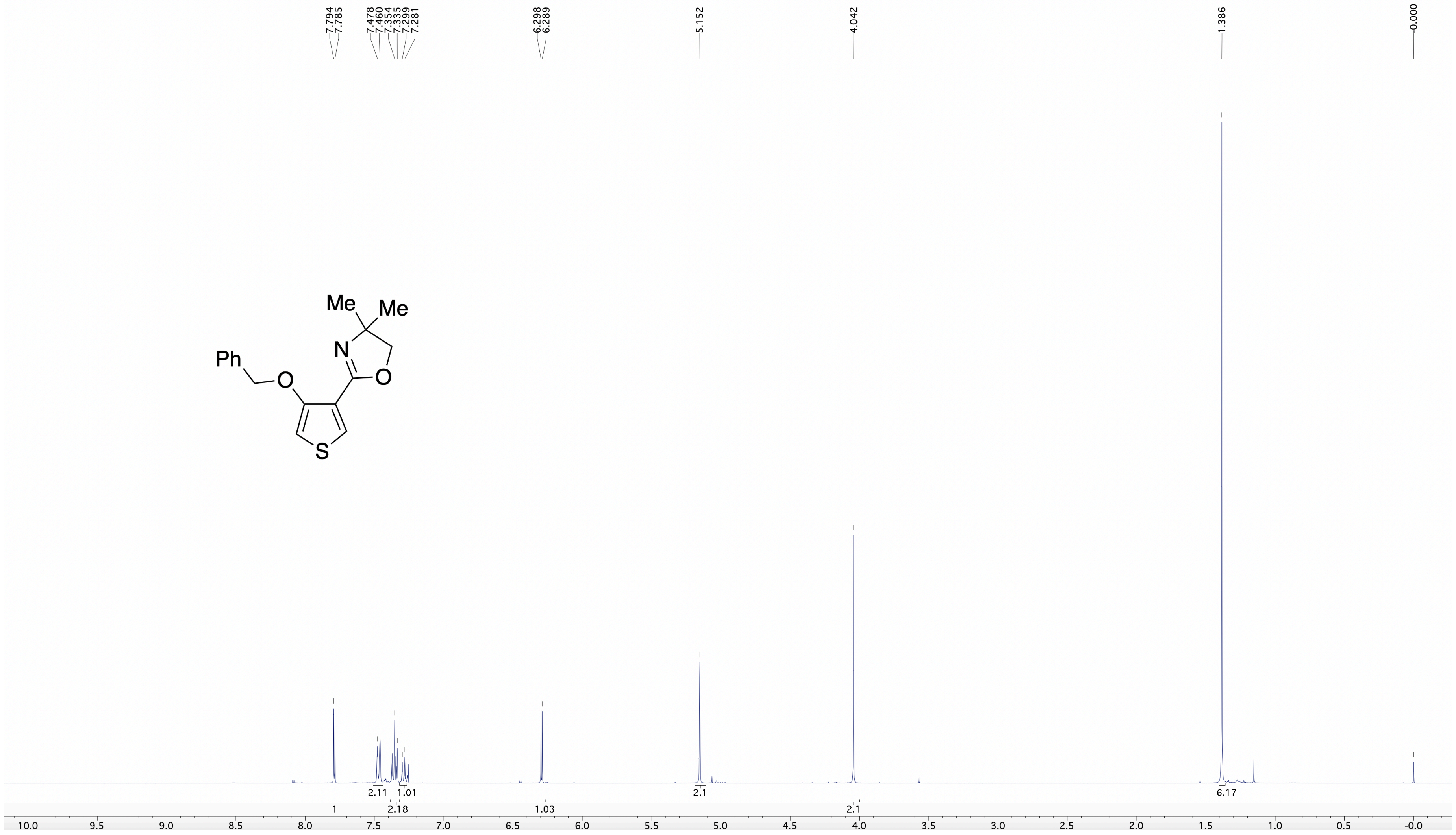

Figure S33. 100 MHz DEPTQ ^{13}C NMR spectrum of **13**

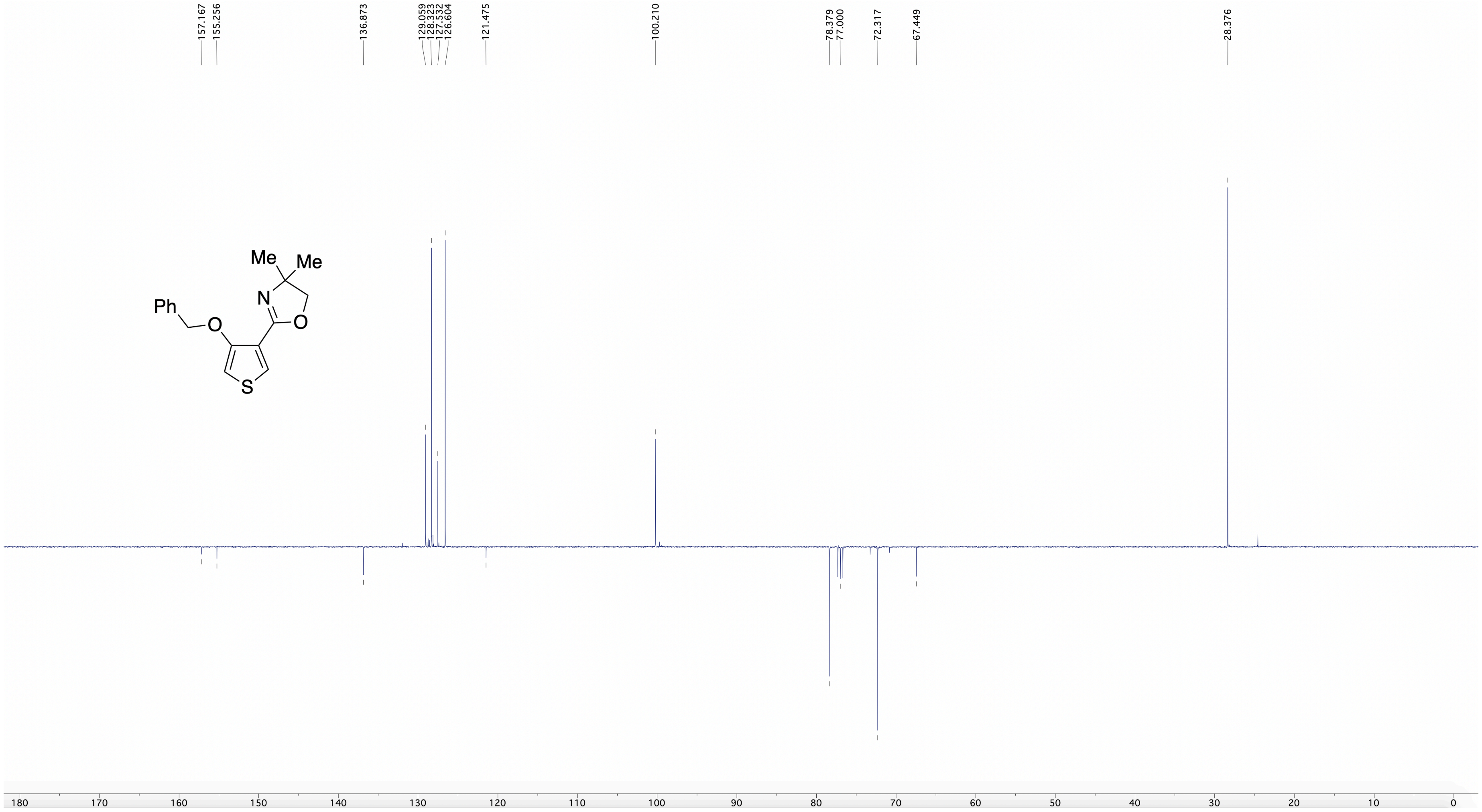

Figure S34. 400 MHz ^{1}H NMR spectrum of **45**

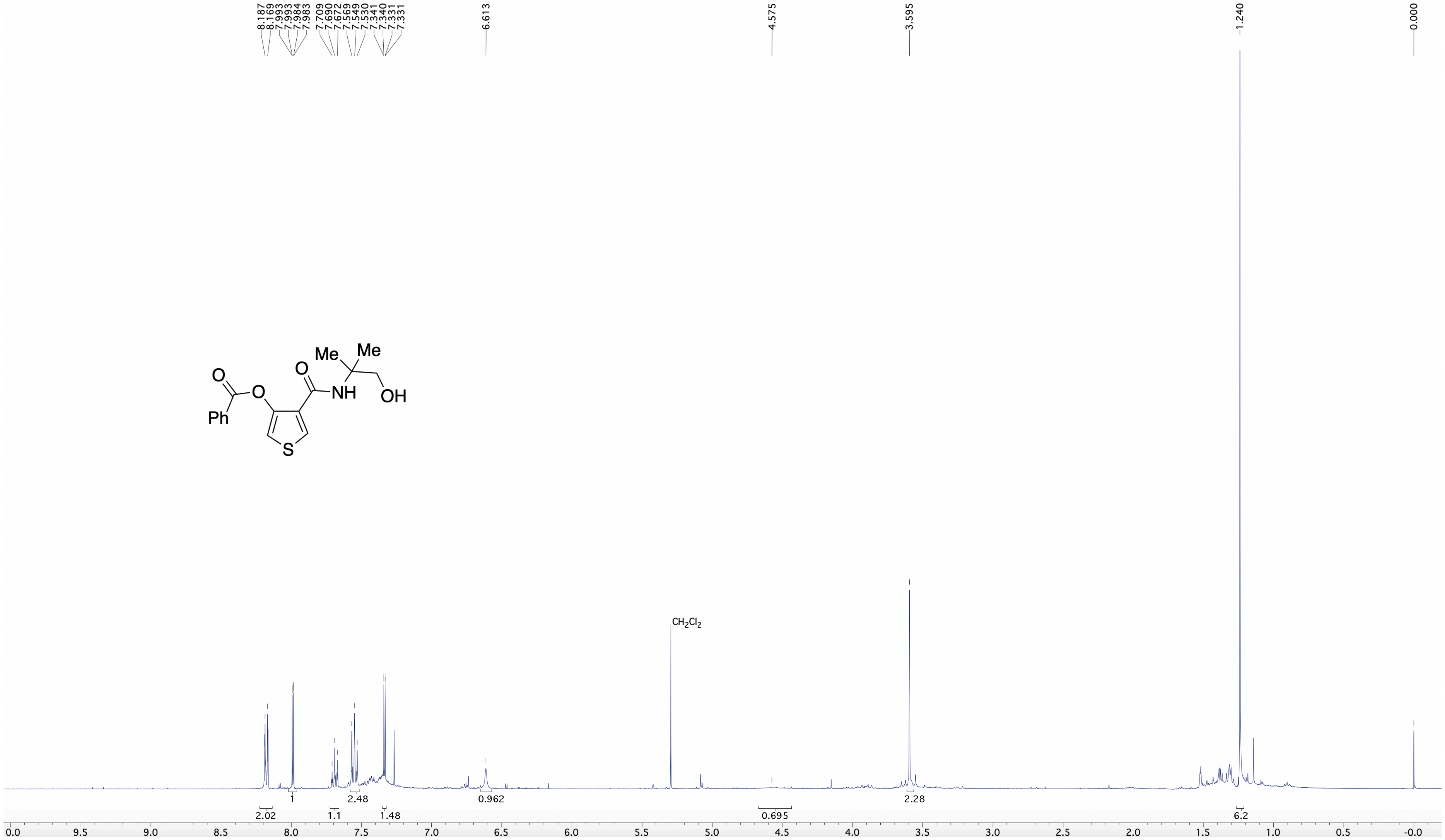

Figure S35. 125 MHz DEPTQ ^{13}C NMR spectrum of **45**

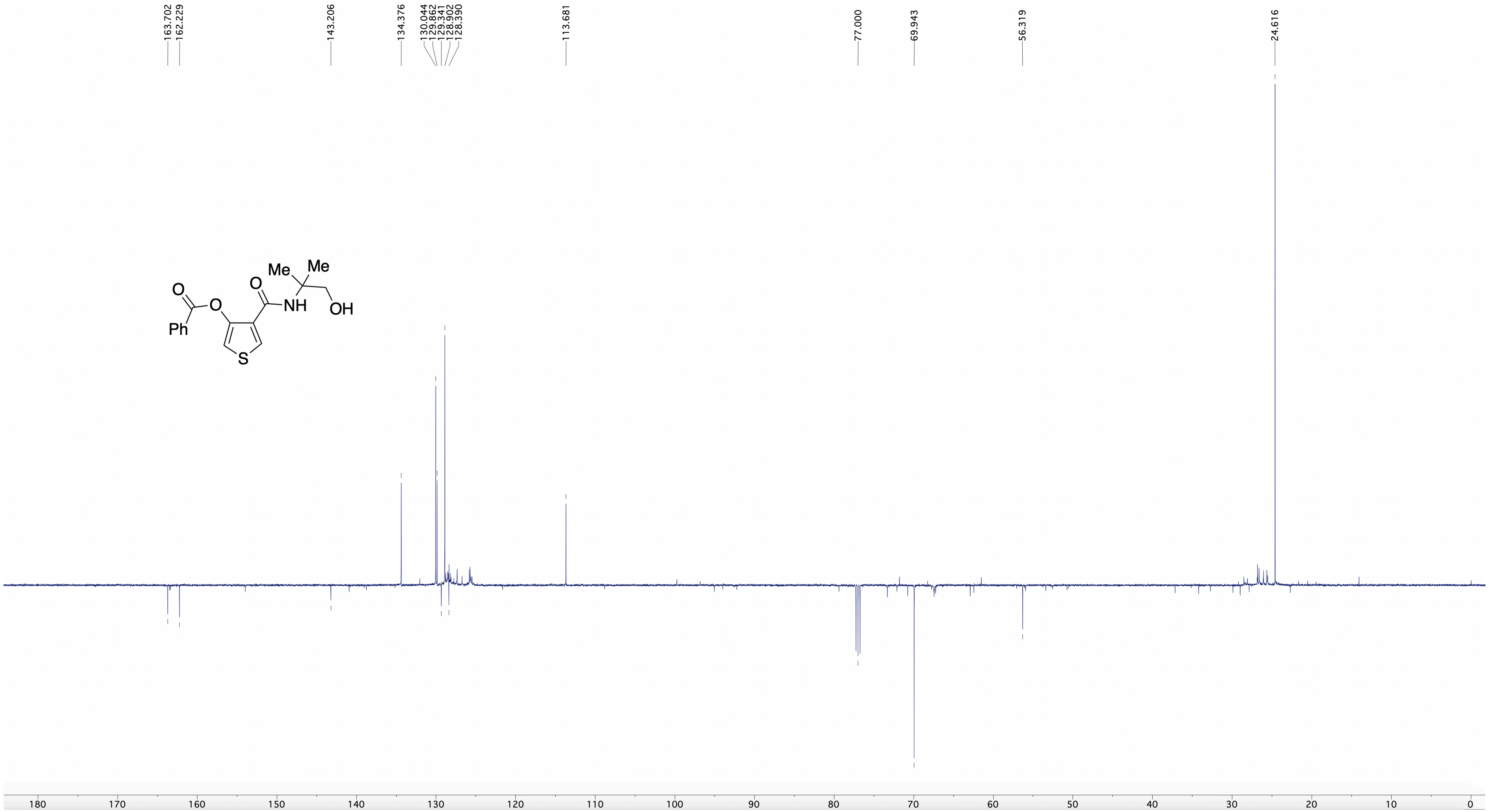

Figure S36. 400 MHz ^{1}H NMR spectrum of **14**

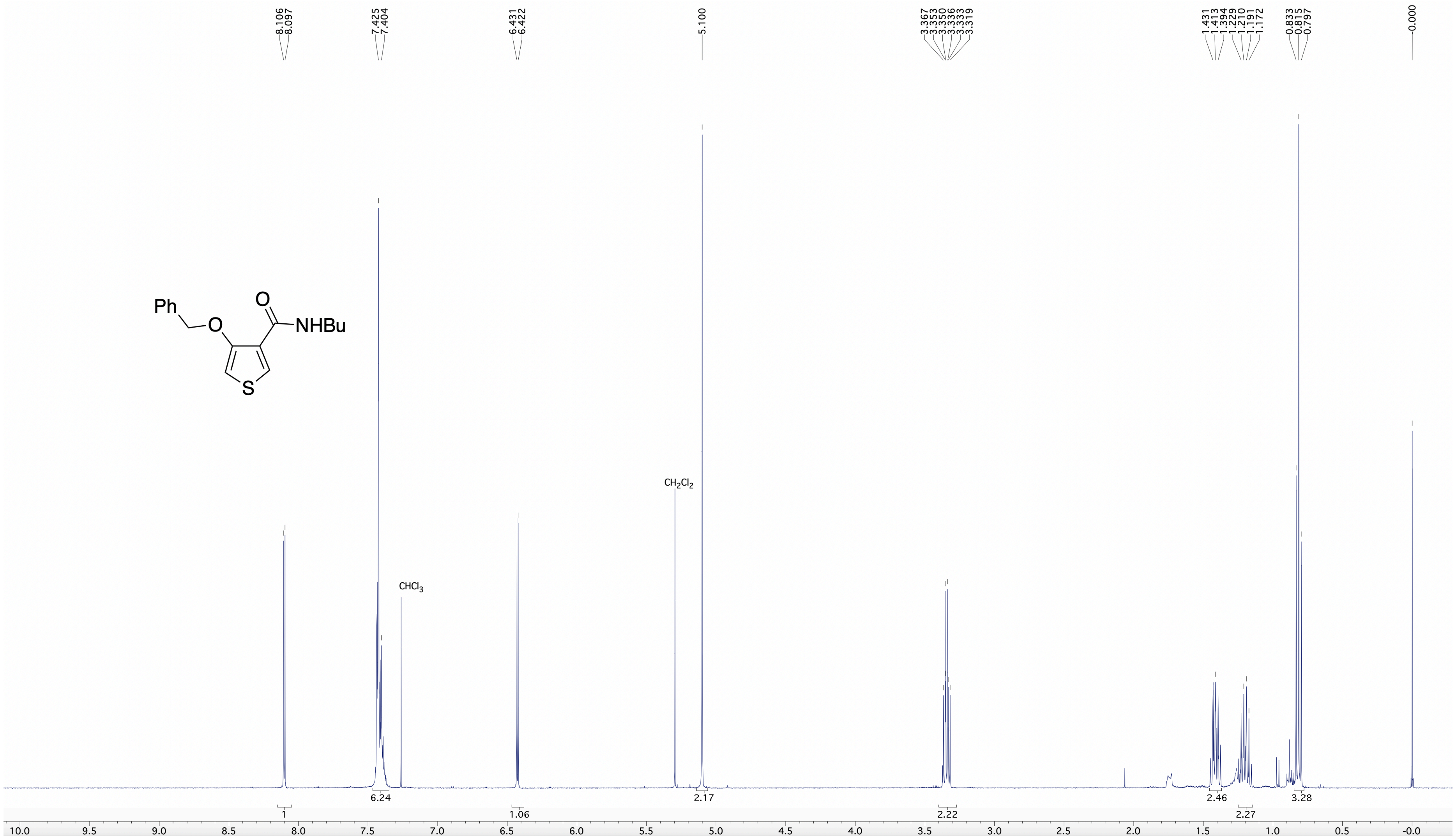

Figure S37. 10 MHz DEPTQ ^{13}C NMR spectrum of **14**

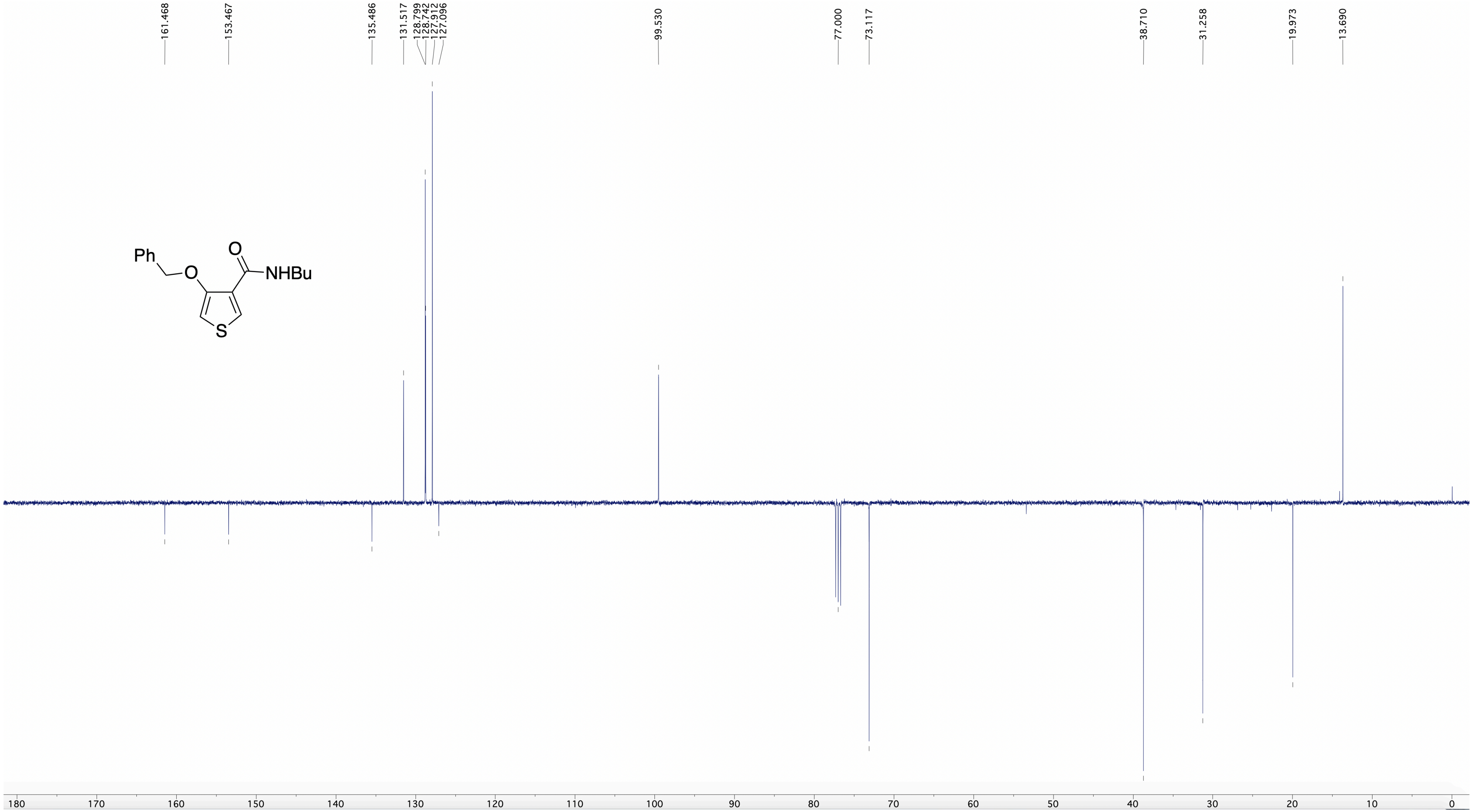

Figure S38. 400 MHz ^{1}H NMR spectrum of **46**

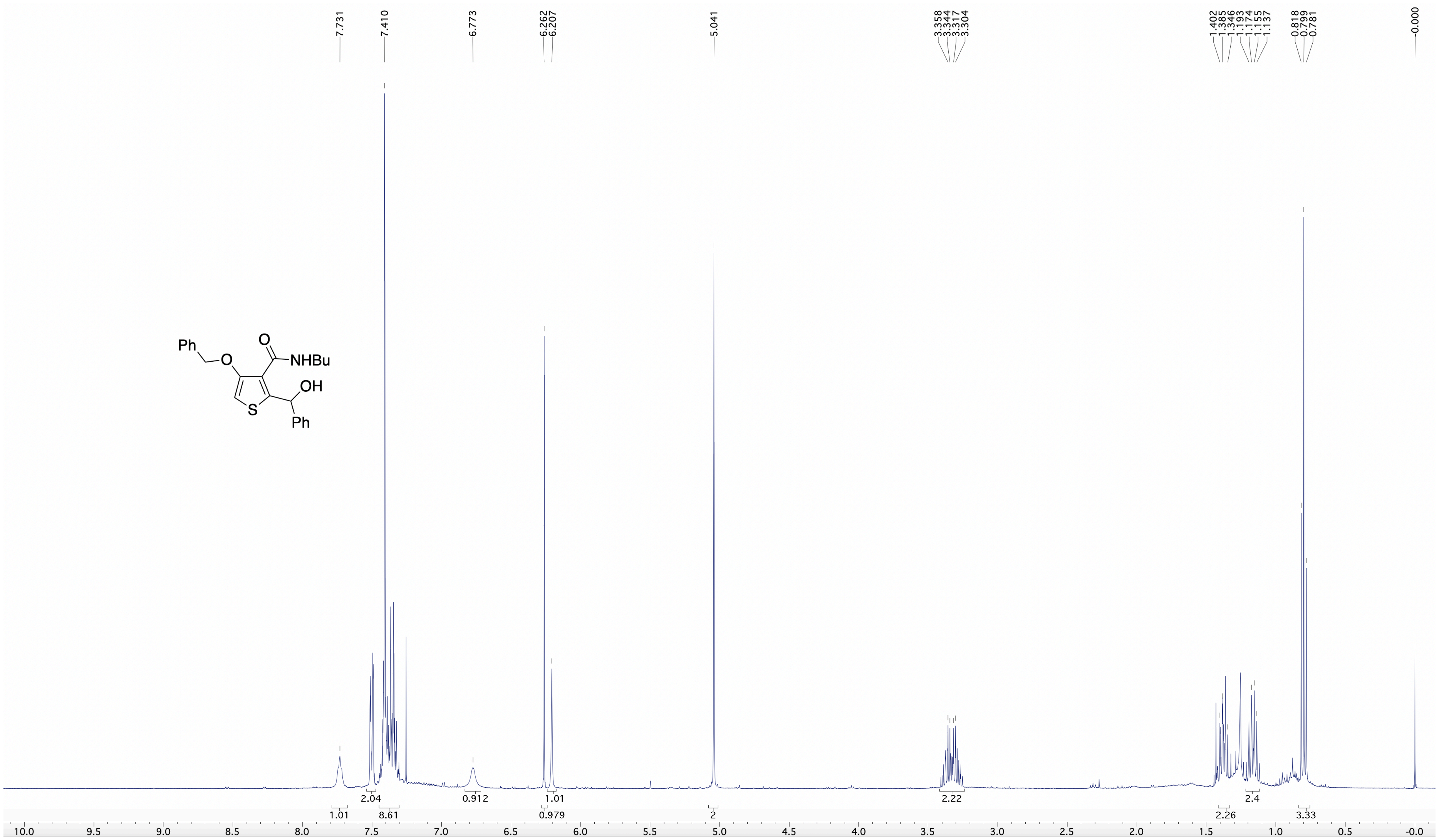

Figure S39. 100 MHz DEPTQ ^{13}C NMR spectrum of **46**

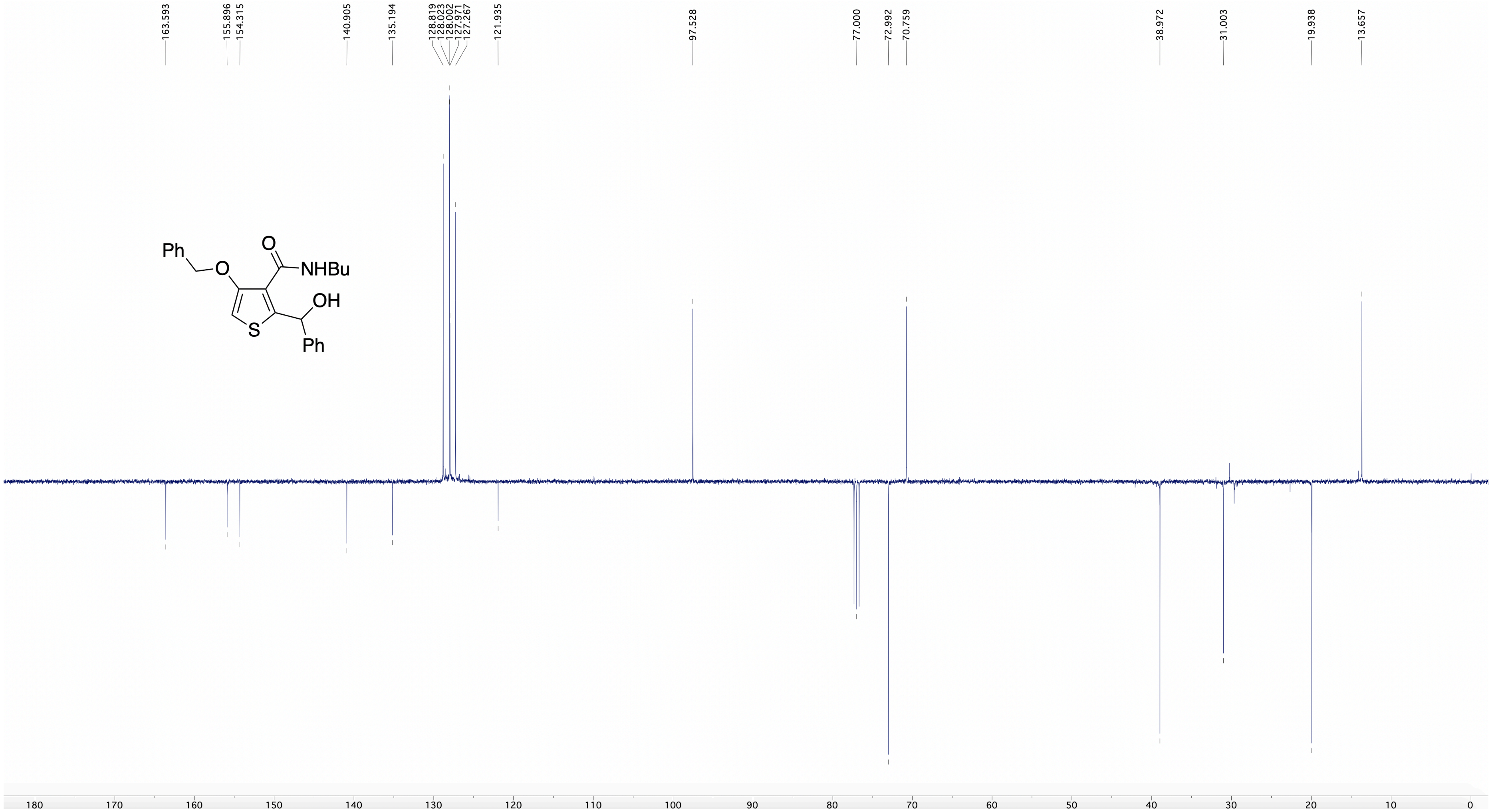

Figure 40. 500 MHz ^{1}H NMR spectrum of **47**

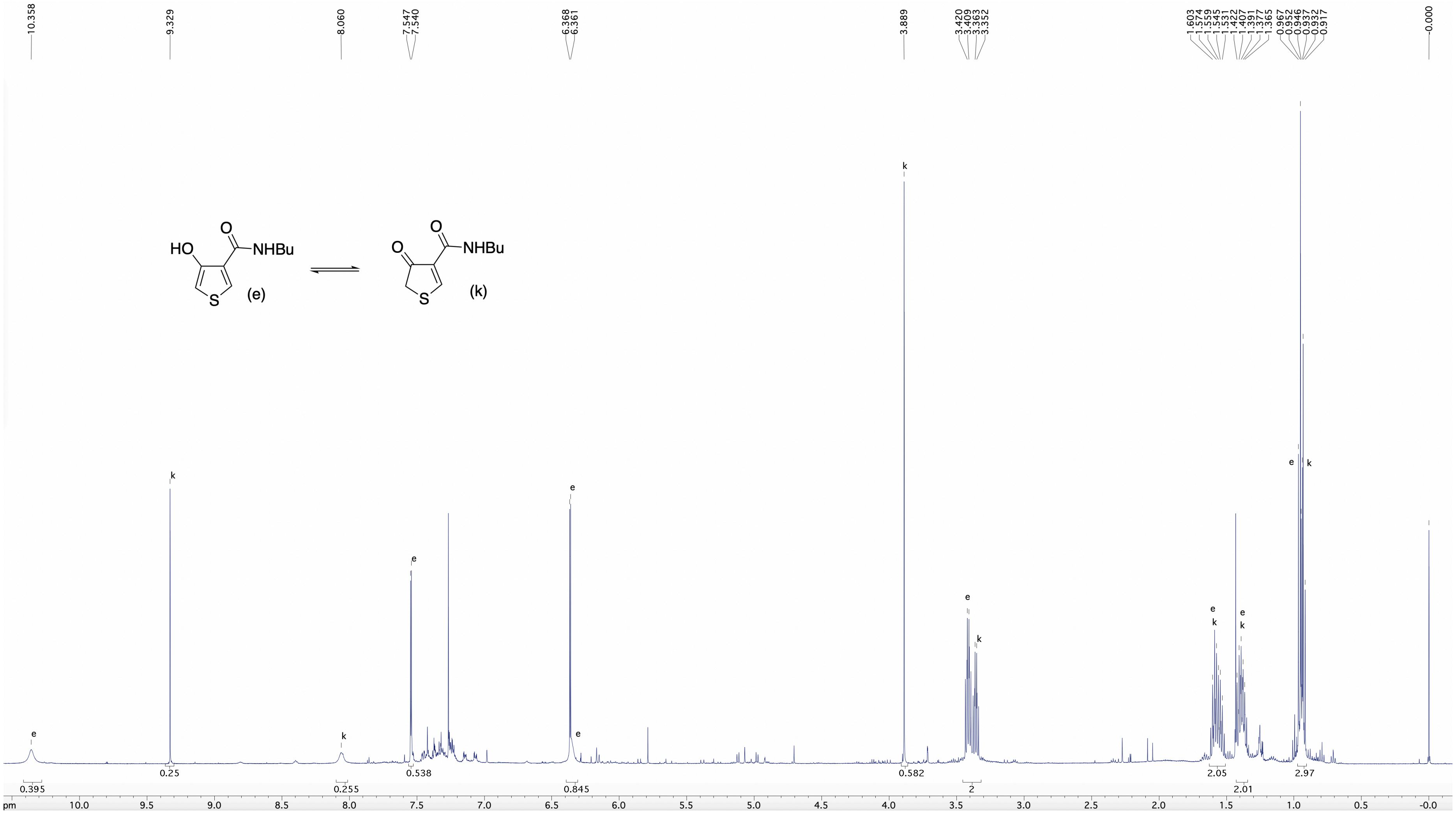

Figure S41. 125 MHz DEPTQ ^{13}C NMR spectrum of **47**

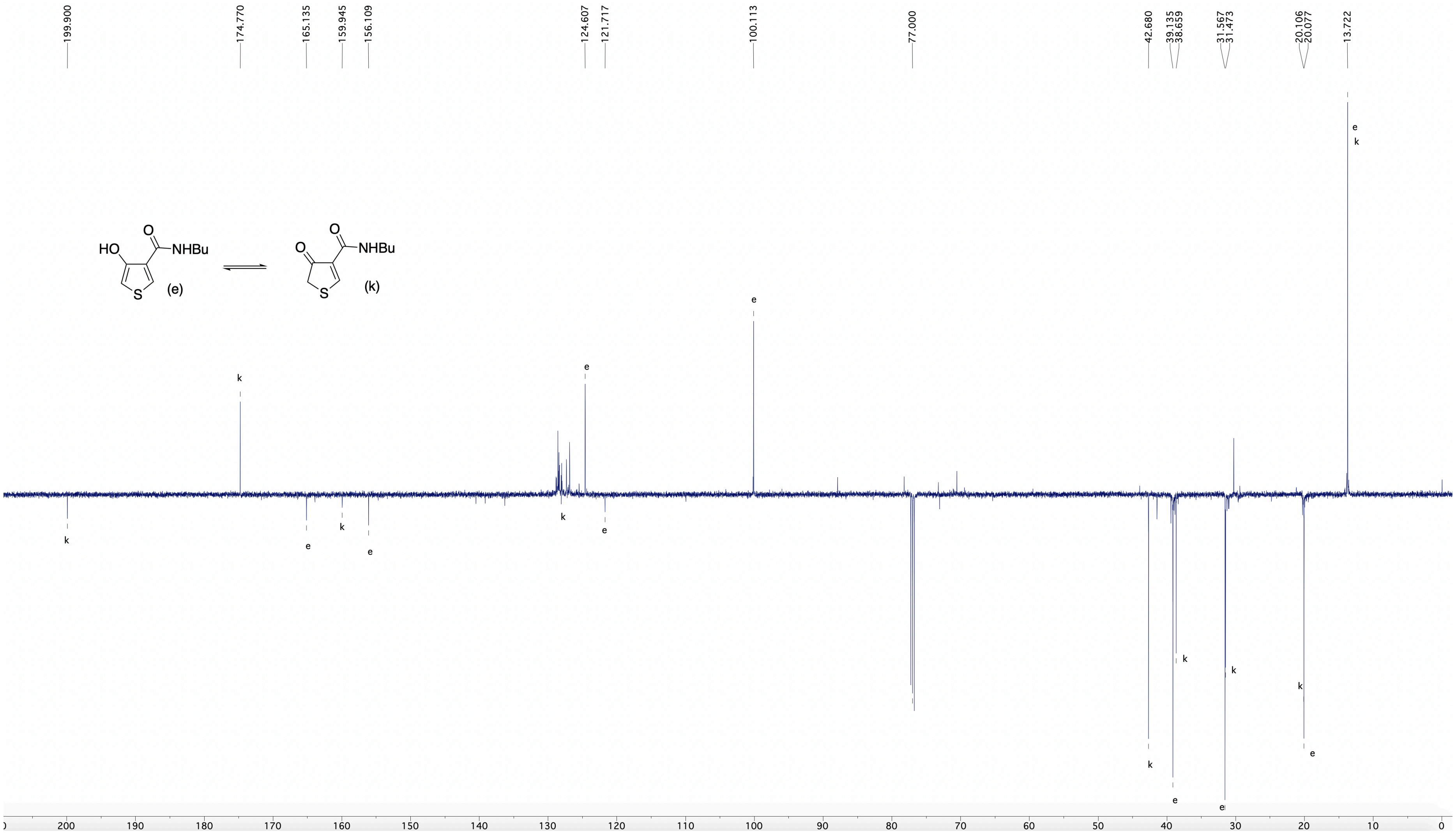

Supplement: Supplementary file 1 [file molecules-26-07690-s001.zip › 21Mol supp mater/21Molecules_AH_RAA_suppl rev.pdf]
