# Supplementary material for: Rationalisation of Patterns of Competing Reactivity by X-ray Structure Determination: Reaction of Isomeric (Benzyloxythienyl)oxazolines with a Base
Source: Molecules. 2021 Dec 20;26(24):7690. doi: 10.3390/molecules26247690 (PMC8705950; doi:10.3390/molecules26247690)

checkCIF/PLATON report

Structure factors have been supplied for datablock(s) 13

THIS REPORT IS FOR GUIDANCE ONLY. IF USED AS PART OF A REVIEW PROCEDURE FOR PUBLICATION, IT SHOULD NOT REPLACE THE EXPERTISE OF AN EXPERIENCED CRYSTALLOGRAPHIC REFEREE.

No syntax errors found. CIF dictionary Interpreting this report

Datablock: 13

Bond precision:	C-C = 0.0018 A		Wavelength=0.71075
Cell:	a=15.6319(4)	b=8.7152(3)	c=10.7572(3)
	alpha=90	beta=93.909(3)	gamma=90
Temperature:	296 K		

	Calculated	Reported
Volume	1462.10(8)	1462.10(7)
Space group	P 21/c	P 1 21/c 1
Hall group	-P 2ybc	-P 2ybc
Moiety formula	C16 H17 N O2 S	C16 H17 N O2 S
Sum formula	C16 H17 N O2 S	C16 H17 N O2 S
Mr	287.37	287.38
Dx,g cm-3	1.306	1.305
Z	4	4
Mu (mm-1)	0.222	0.222
F000	608.0	608.0
F000'	608.74	
h,k,lmax	20,11,14	20,11,14
Nref	3662	3217
Tmin,Tmax	0.957,0.998	0.731,0.998
Tmin'	0.957	

Correction method= # Reported T Limits: Tmin=0.731 Tmax=0.998
AbsCorr = MULTI-SCAN

Data completeness= 0.878 Theta(max)= 28.355

R(reflections)= 0.0333(2781) wR2(reflections)= 0.0917(3217)

S = 1.054 Npar= 181

```
The following ALERTS were generated. Each ALERT has the format
       test-name_ALERT_alert-type_alert-level.
Click on the hyperlinks for more details of the test.
```

Alert level G

```
PLAT398_ALERT_2_G Deviating  C-O-C    Angle From 120 for O1            104.3 Degree
PLAT882_ALERT_1_G No Datum for _diffrn_reflns_av_unetI/netI ......     Please Do !
PLAT910_ALERT_3_G Missing # of FCF Reflection(s) Below Theta(Min).         1 Note
PLAT912_ALERT_4_G Missing # of FCF Reflections Above STh/L=  0.600       446 Note
PLAT978_ALERT_2_G Number C-C Bonds with Positive Residual Density.        14 Info
```

```
   0 ALERT level A = Most likely a serious problem - resolve or explain
   0 ALERT level B = A potentially serious problem, consider carefully
   0 ALERT level C = Check. Ensure it is not caused by an omission or oversight
   5 ALERT level G = General information/check it is not something unexpected

   1 ALERT type 1 CIF construction/syntax error, inconsistent or missing data
   2 ALERT type 2 Indicator that the structure model may be wrong or deficient
   1 ALERT type 3 Indicator that the structure quality may be low
   1 ALERT type 4 Improvement, methodology, query or suggestion
   0 ALERT type 5 Informative message, check
```

It is advisable to attempt to resolve as many as possible of the alerts in all categories. Often the minor alerts point to easily fixed oversights, errors and omissions in your CIF or refinement strategy, so attention to these fine details can be worthwhile. In order to resolve some of the more serious problems it may be necessary to carry out additional measurements or structure refinements. However, the purpose of your study may justify the reported deviations and the more serious of these should normally be commented upon in the discussion or experimental section of a paper or in the "special_details" fields of the CIF. checkCIF was carefully designed to identify outliers and unusual parameters, but every test has its limitations and alerts that are not important in a particular case may appear. Conversely, the absence of alerts does not guarantee there are no aspects of the results needing attention. It is up to the individual to critically assess their own results and, if necessary, seek expert advice.

Publication of your CIF in IUCr journals

A basic structural check has been run on your CIF. These basic checks will be run on all CIFs submitted for publication in IUCr journals (*Acta Crystallographica*, *Journal of Applied Crystallography*, *Journal of Synchrotron Radiation*); however, if you intend to submit to *Acta Crystallographica Section C* or *E* or *IUCrData*, you should make sure that full publication checks are run on the final version of your CIF prior to submission.

Publication of your CIF in other journals

Please refer to the *Notes for Authors* of the relevant journal for any special instructions relating to CIF submission.

PLATON version of 13/07/2021; check.def file version of 13/07/2021

Datablock 13 - ellipsoid plot

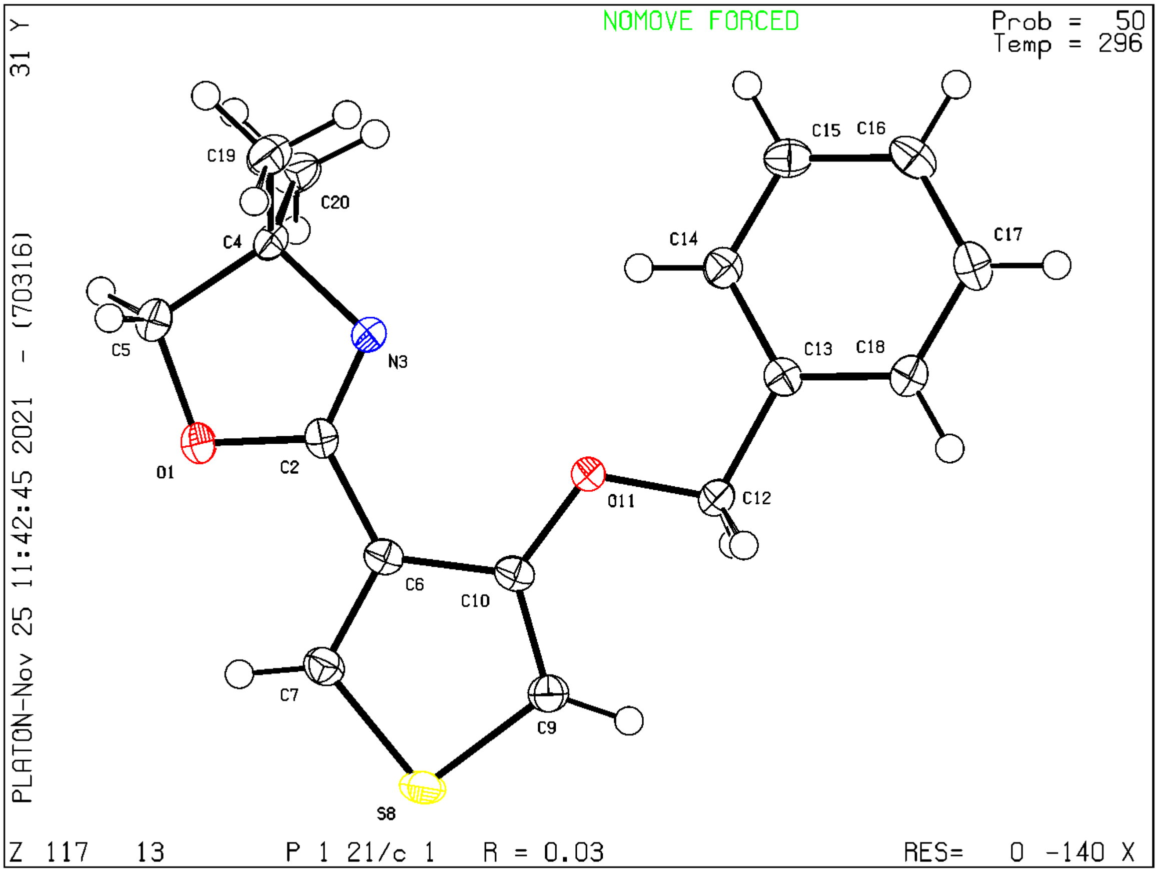

Supplement: Supplementary file 1 [file molecules-26-07690-s001.zip › 21Mol supp mater/checkcif 13.pdf]
