# Supplementary material for: Rationalisation of Patterns of Competing Reactivity by X-ray Structure Determination: Reaction of Isomeric (Benzyloxythienyl)oxazolines with a Base
Source: Molecules. 2021 Dec 20;26(24):7690. doi: 10.3390/molecules26247690 (PMC8705950; doi:10.3390/molecules26247690)
Supplement: Supplementary file 1 [file checkcif 20.pdf › 21Mol supp mater/checkcif 20.pdf]

checkCIF/PLATON report

Structure factors have been supplied for datablock(s) 20

THIS REPORT IS FOR GUIDANCE ONLY. IF USED AS PART OF A REVIEW PROCEDURE FOR PUBLICATION, IT SHOULD NOT REPLACE THE EXPERTISE OF AN EXPERIENCED CRYSTALLOGRAPHIC REFEREE.

No syntax errors found. CIF dictionary Interpreting this report

Datablock: 20

Bond precision: C-C = 0.0089 A   Wavelength=0.71075

Cell:	a=17.45200	b=9.98400	c=21.10500
	alpha=90	beta=92.3760	gamma=90
Temperature:	93 K		

	Calculated	Reported
Volume	3674.190	3674
Space group	P 21/n	P 1 21/n 1
Hall group	-P 2yn	-P 2yn
Moiety formula	2(C16 H17 N O3 S), C3 H6 O, C H2 Cl2	C36 H42 Cl2 N2 O7 S2
Sum formula	C36 H42 Cl2 N2 O7 S2	C36 H42 Cl2 N2 O7 S2
Mr	749.74	749.76
Dx,g cm-3	1.355	1.355
Z	4	4
Mu (mm-1)	0.340	0.340
F000	1576.0	1576.0
F000'	1578.83	
h,k,lmax	20,12,25	20,11,25
Nref	6680	6666
Tmin,Tmax	0.967,0.967	0.699,0.967
Tmin'	0.967	

Correction method= # Reported T Limits: Tmin=0.699 Tmax=0.967
AbsCorr = MULTI-SCAN

Data completeness= 0.998   Theta(max)= 25.312

R(reflections)= 0.0929(4473)   wR2(reflections)= 0.2732(6666)

S = 1.077   Npar= 458

The following ALERTS were generated. Each ALERT has the format
test-name_ALERT_alert-type_alert-level.
Click on the hyperlinks for more details of the test.

Alert level B

```
PLAT097_ALERT_2_B Large Reported Max.  (Positive) Residual Density       2.38 eA-3
```

Author Response: Disorder in chlorinated solvent.

Alert level C

```
DIFMX02_ALERT_1_C  The maximum difference density is > 0.1*ZMAX*0.75
            The relevant atom site should be identified.
PLAT084_ALERT_3_C High wR2 Value (i.e. > 0.25) ..................       0.27 Report
PLAT094_ALERT_2_C Ratio of Maximum / Minimum Residual Density ....       2.36 Report
PLAT141_ALERT_4_C s.u. on a - Axis Small or Missing .............    0.00000 Ang.
PLAT142_ALERT_4_C s.u. on b - Axis Small or Missing .............    0.00000 Ang.
PLAT143_ALERT_4_C s.u. on c - Axis Small or Missing .............    0.00000 Ang.
PLAT145_ALERT_4_C s.u. on beta     Small or Missing .............     0.0000 Degree
PLAT151_ALERT_1_C No s.u. (esd) Given on Volume .................     Please Do !
PLAT244_ALERT_4_C Low    'Solvent' Ueq as Compared to Neighbors of        C42 Check
PLAT340_ALERT_3_C Low Bond Precision on  C-C Bonds ..............    0.00894 Ang.
PLAT906_ALERT_3_C Large K Value in the Analysis of Variance ......      7.201 Check
PLAT910_ALERT_3_C Missing # of FCF Reflection(s) Below Theta(Min).          5 Note
PLAT971_ALERT_2_C Check Calcd Resid. Dens.  1.26A   From Cl1             2.30 eA-3
PLAT971_ALERT_2_C Check Calcd Resid. Dens.  1.28A   From C44             1.92 eA-3
```

Alert level G

```
PLAT002_ALERT_2_G Number of Distance or Angle Restraints on AtSite          8 Note
PLAT042_ALERT_1_G Calc. and Reported Moiety Formula Strings Differ     Please Check
PLAT072_ALERT_2_G SHELXL First  Parameter in WGHT  Unusually Large       0.13 Report
PLAT083_ALERT_2_G SHELXL Second Parameter in WGHT  Unusually Large      14.31 Why ?
PLAT172_ALERT_4_G The CIF-Embedded .res File Contains DFIX Records          1 Report
PLAT380_ALERT_4_G Incorrectly? Oriented X(sp2)-Methyl Moiety .....        C41 Check
PLAT380_ALERT_4_G Incorrectly? Oriented X(sp2)-Methyl Moiety .....        C43 Check
PLAT434_ALERT_2_G Short Inter HL..HL Contact Cl2      ..Cl2              3.36 Ang.
                                             1-x,1-y,1-z  =      3_666 Check
PLAT793_ALERT_4_G Model has Chirality at C7          (Centro SPGR)          R Verify
PLAT793_ALERT_4_G Model has Chirality at C27         (Centro SPGR)          S Verify
PLAT860_ALERT_3_G Number of Least-Squares Restraints ............           4 Note
PLAT882_ALERT_1_G No Datum for _diffrn_reflns_av_unetI/netI ......     Please Do !
PLAT909_ALERT_3_G Percentage of I>2sig(I) Data at Theta(Max) Still        39% Note
PLAT912_ALERT_4_G Missing # of FCF Reflections Above STh/L=  0.600          9 Note
PLAT978_ALERT_2_G Number C-C Bonds with Positive Residual Density.          2 Info
```

0 **ALERT level A** = Most likely a serious problem - resolve or explain
1 **ALERT level B** = A potentially serious problem, consider carefully
14 **ALERT level C** = Check. Ensure it is not caused by an omission or oversight
15 **ALERT level G** = General information/check it is not something unexpected

```
 4 ALERT type 1 CIF construction/syntax error, inconsistent or missing data
 9 ALERT type 2 Indicator that the structure model may be wrong or deficient
 6 ALERT type 3 Indicator that the structure quality may be low
11 ALERT type 4 Improvement, methodology, query or suggestion
 0 ALERT type 5 Informative message, check
```

It is advisable to attempt to resolve as many as possible of the alerts in all categories. Often the minor alerts point to easily fixed oversights, errors and omissions in your CIF or refinement strategy, so attention to these fine details can be worthwhile. In order to resolve some of the more serious problems it may be necessary to carry out additional measurements or structure refinements. However, the purpose of your study may justify the reported deviations and the more serious of these should normally be commented upon in the discussion or experimental section of a paper or in the "special_details" fields of the CIF. checkCIF was carefully designed to identify outliers and unusual parameters, but every test has its limitations and alerts that are not important in a particular case may appear. Conversely, the absence of alerts does not guarantee there are no aspects of the results needing attention. It is up to the individual to critically assess their own results and, if necessary, seek expert advice.

Publication of your CIF in IUCr journals

A basic structural check has been run on your CIF. These basic checks will be run on all CIFs submitted for publication in IUCr journals (*Acta Crystallographica*, *Journal of Applied Crystallography*, *Journal of Synchrotron Radiation*); however, if you intend to submit to *Acta Crystallographica Section C* or *E* or *IUCrData*, you should make sure that full publication checks are run on the final version of your CIF prior to submission.

Publication of your CIF in other journals

Please refer to the *Notes for Authors* of the relevant journal for any special instructions relating to CIF submission.

PLATON version of 13/07/2021; check.def file version of 13/07/2021
